# *TSC1* and *DEPDC5* regulate HIV-1 latency through the mTOR signaling pathway

**DOI:** 10.1038/s41426-018-0139-5

**Published:** 2018-08-08

**Authors:** Shan Jin, Qibin Liao, Jian Chen, Linxia Zhang, Qian He, Huanzhang Zhu, Xiaoyan Zhang, Jianqing Xu

**Affiliations:** 1 Shanghai Public Health Clinical Center and Institutes of Biomedical Sciences, Key Laboratory of Medical Molecular Virology, Shanghai Medical College, Fudan University, Shanghai, China; 2 State Key Laboratory of Genetic Engineering and Key Laboratory of Medical Molecular Virology of Ministry of Education/Health, School of Life Sciences, Fudan University, Shanghai, China; 3 State Key Laboratory for Infectious Disease Prevention and Control, China Centers for Disease Control and Prevention, Beijing, China

## Abstract

The latent reservoir of HIV-1 presents a major barrier to viral eradication. The mechanism of the establishment and maintenance of the latent viral reservoir is not yet fully understood, which hinders the development of effective curative strategies. In this study, we identified two inhibitory genes, *TSC1* and *DEPDC5*, that maintained HIV-1 latency by suppressing the mTORC1 pathway. We first adapted a genome-wide CRISPR screening approach to identify host factors required for HIV latency in a T-cell-based latency model and discovered two inhibitory genes, *TSC1* and *DEPDC5*, which are potentially involved in HIV-1 latency. Knockout of either *TSC1* or *DEPDC5* led to enhanced HIV-1 reactivation in both a T-cell line (C11) and a monocyte cell line (U1), and this enhancement could be antagonized by the mTORC1 inhibitor rapamycin. Further evaluation of the mechanism revealed that *TSC1* suppresses AKT-mTORC1-S6 via downregulation of Rheb, whereas *DEPDC5* inhibits AKT-mTORC1-S6 through RagA. Overall, both *TSC1* and *DEPDC5* negatively regulate the AKT-mTORC1 pathway, and thus their agonists could be used in the development of new therapeutic approaches for activating HIV-1 latency.

## Introduction

Highly active antiretroviral therapy (HAART), a potent, life-long and lasting treatment approach, is not a curative treatment for human immunodeficiency virus type 1 (HIV-1)-infected patients, as interruption of treatment results in a rapid rebound of viremia within 2–8 weeks^1,2^. The latent reservoir presents a major barrier to curing HIV-1 infection. However, the mechanism of the establishment and maintenance of the latent viral reservoir is not yet fully understood, which hinders the development of effective curative strategies.

There have been discrepancies in the differentiation and maintenance of HIV-1 latently infected cells. It was previously proposed that HIV-1 latency is established when activated CD4^+^ T cells become infected and then differentiate into resting memory cells that are non-permissive for viral gene expression^3^. The CD4^+^ T cells that undergo this activated-to-resting transition might be the major cellular target that supports the establishment of HIV-1 latent infection^4^. However, other studies have suggested that HIV latency can arise from the direct infection of both resting and activated CD4^+^ T cells^5,6^. In addition to memory T cells, the latent virus possibly resides in other cell subsets, including perivascular macrophages, microglia, astrocytes and dendritic cells (DCs), even in patients on HAART^7–9^. The persistence of latent HIV-1 could result from the long half-life of these cells but may also be due to homeostatic proliferation^2^.

Most studies have focused on the transcriptional and epigenetic regulation of HIV-1 latency to explore novel technologies for silencing HIV-1 expression or eradicating HIV-1 provirus from host genomes in infected individuals. It was previously reported that transcriptional interference antagonizes proviral gene expression to promote HIV latency and that chromatin reassembly factors have an important role in maintaining HIV latency^10,11^. The lack of host transcriptional activators, including nuclear factor-κB (NF-κB)^12,13^, NFAT^14^, Sp1^15^, and AP-1^16^, or the presence of host transcriptional repressors, including CBF-1^17^ and TCF-4^18^, can affect the transcriptional status of the latent virus. The inducible/silent phenotype appears to be associated with the integration site of the provirus. The provirus was more inducible when integrated in gene deserts, whereas gene expression was more difficult to initiate if the provirus was integrated in centromeric heterochromatin^19,20^. Serial analyses of gene expression demonstrated that more than 90% of the host genes harboring a latent integrated provirus were transcriptionally active, which suggested that disrupting the negative control of HIV-1 transcription by upstream host promoters could facilitate reactivation of latent HIV-1^21^. The histone acetyltransferase hGCN5 has also been reported to enhance Tat-dependent transcription of the HIV-1 long terminal repeat (LTR)^22^. Inhibition of transcription interference from host genes, such as blocking the access of factors to the downstream promoter or dislodging bound proteins, transcriptional train-wrecking, RNA interference, DNA methylation, induction of the interferon response, or generation of antisense RNA, provides a potential mechanism to disrupt latency.

The cell signaling pathway involved in the generation and maintenance of memory CD4^+^ T cells has also been suggested to regulate the induction of latency and the persistence of the HIV reservoir. Kulpa et al.^23^ showed that the canonical Wnt/b-catenin pathway is a critical component in self-renewal of CD4^+^ Tscm and Tcm populations and may function as a mechanism for maintaining cells containing the HIV latent reservoir. Several studies reported that high levels of Notch signaling induce quiescence, whereas low levels promote differentiation and proliferation of CD4^+^ T cells^24,25^. In addition, mechanistic target of rapamycin (mTOR) is an important regulator of glucose metabolism and connects cell growth, energy balance, and aging to metabolic processes^26^. HIV-associated fundamental changes to this metabolic machinery of the immune system can promote a state of “inflammaging,” a chronic, low-grade inflammation with specific immune changes, and can also contribute to the persistence of HIV in its reservoirs^27^. Inhibition of mTORC1 or PI3Kinase can successfully inhibit viral replication and viral reactivation as a result of a decrease in cellular biosynthesis^28^. Furthermore, Besnard et al.^29^ unveiled that knocking down mTOR results in the enhancement of HIV latency in a pooled short hairpin RNA (shRNA) screening assay. These findings suggest that multiple pathways may be engaged in the regulation of HIV latency and pose the great challenge of purging the HIV reservoir.

The “shock and kill” approach has been widely discussed in recent studies for eliminating the long-lived HIV-1 reservoir, which aims to purge the provirus from latency with anti-latency drugs while the patient continues antiretroviral therapy^30^. Latency reversal agents (LRAs), including histone deacetylase inhibitors^31^, protein kinase C activators^32^, and other small compounds with unclear mechanisms^33^, induce relaxation of the chromatin at the HIV-1 promoter, thus inducing viral gene expression and reversing the viral latency^30^. However, none of these viral inducers have shown a significant effect on the viral reservoir in clinical trials^34,35^.

In addition, despite many proposed mechanisms, we do not fully understand what controls latency. Therefore, further research on the basic mechanisms of HIV-1 latency remains a priority. Genetic screens are powerful tools for identification of novel genes and help us to understand host–virus interactions, as well as identify the pathways that can be targeted by preventive or therapeutic interventions. In this work, for the first time, we adapted a genome-wide CRISPR screening approach to identify host factors required for HIV latency. We discovered several inhibitory genes, including *DEPDC5* and *TSC1*, which maintain HIV-1 latency by acting as upstream regulators of mTORC1.

## Results

### Genome-wide CRISPR screening enriches host factors and pathways associated with HIV latency

**Fig. 1:**
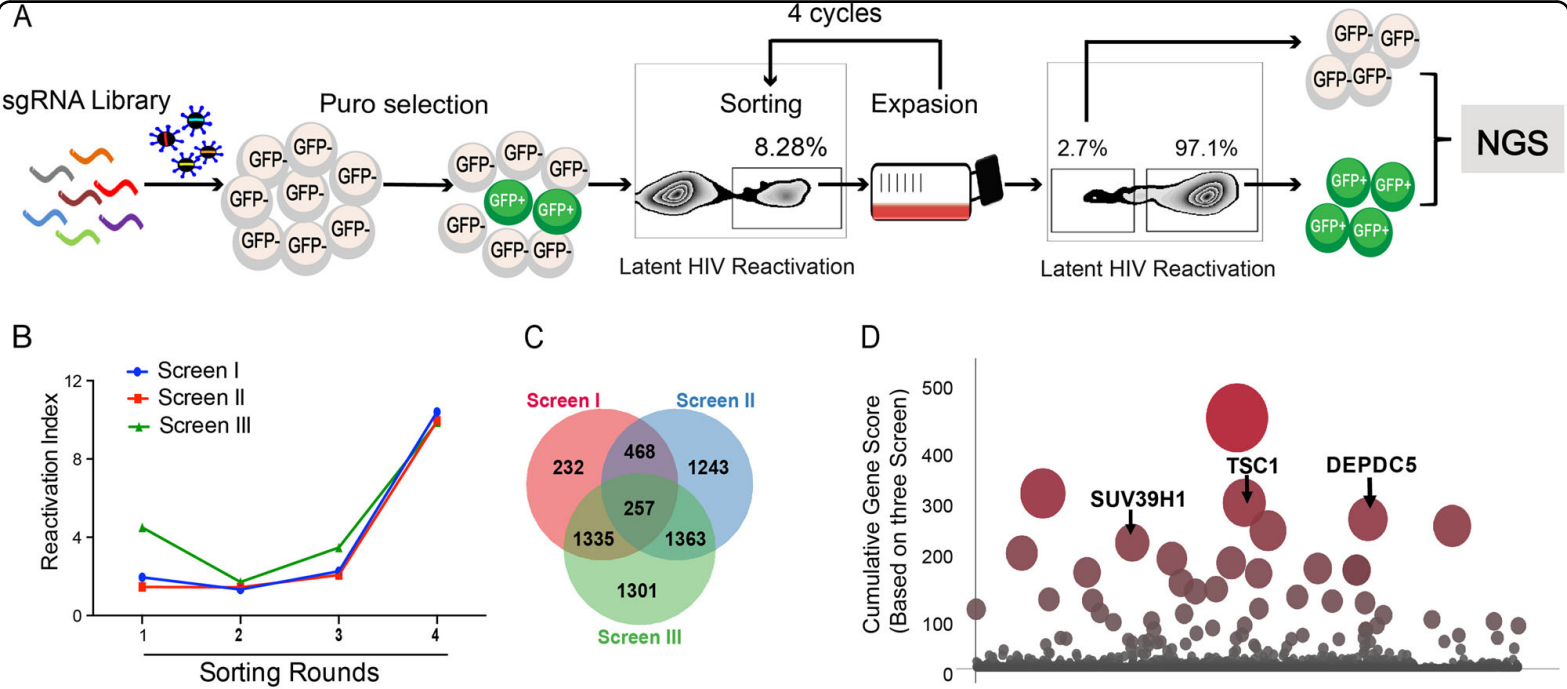
Genome-wide CRISPR screening enriches host factors. **a** Overview of the experimental design of the lentiCRISPRv2.0 library screen. Spontaneous latency reversal in C11 cells was quantified by gating GFP^+^ cells in a flow cytometry assay. Screening was enriched through four rounds, and GFP^+^ cells were harvested at each round. As a control, the GFP^−^cell population was also harvested at the last round. High-throughput sequencing was subsequently used to compare selected cells with controls. **b** Three independent biological replicates of this screen (named screens I, II, and III) are shown. The horizontal coordinates of the line graph represent the number of enrichment rounds, and the vertical coordinates represent the percentage of GFP expression in the experimental group / the percentage of GFP expression in the control group. **c** Venn diagram of the overlap of all detected sgRNAs from the three replicate screens. **d** Plot of the scores of the top hits in the screen. The height and size of each dot are proportional to the screening score

To identify host genes required for HIV latency, we utilized a genome-wide CRISPR library generated by Zhang’s laboratory^36^. This lentiCRISPRv2.0 library contains over 120,000 gRNAs for 19,050 human genes. This library is divided into two sub-libraries and each sub-library contains three unique single guide RNAs (sgRNAs) targeting one gene in the genome. An HIV-1 latent infection cell line (C11) based on T cells (Jurkat)^37,38^ that harbors an HIV-1 proviral DNA with a reporter gene encoding green fluorescent protein (GFP) was used in this study. Under basal conditions, the HIV genome in the C11 cell line is transcriptionally silent, and C11 intrinsically only produces < 2% GFP-positive cells in in vitro culture (data not shown). After infection with GECKO library lentiviruses and selection with puromycin, the GFP-positive C11 cells increased to 8.28% before the first round of sorting (Fig. 1a). If a gene knockout promotes activation of latent HIV, sgRNAs targeting this gene should be enriched in the GFP-positive cell population. Next, we isolated GFP-positive cells by four rounds of fluorescence-activated cell sorting and enriched the differentially expressed genes (Fig. 1a). Following genomic DNA (gDNA) extraction from cells and PCR amplification of each sgRNA sequence, we performed Illumina sequencing to generate read counts for each gene-targeting lentiCRISPRv2.0 construct. The gene of interest was compared to the distribution of the log2 enrichment values of the negative control sgRNAs and the initial control sgRNAs. We performed three independent biological replicates of this screening (named screens I, II, and III). Each independent repeat experiment was highly enriched relative to the control group in the four rounds of sorting (Fig. 1b). The three screenings had reasonably good overlap, and 257 overlapped genes were identified (Fig. 1c).

We next determined the gene subcellular localization and bio-function by employing Gene Ontology (GO) and Kyoto encyclopedia of genes and genomes (KEGG) analyses. As shown in Supplementary Fig. 1A, the 257 overlapped genes were mainly localized in the nucleus (17.51%) and cytoplasm (15.56%), accounting for up to 33.07% of the genes including *TSC1*, *DEPDC5*, SUV39H1, SPATA6L, and NFKB2. The left genes were mainly localized in the cytoskeleton (9.73%), membranes (7.78%), lysosomes (7.78%), cell junctions (7.78%), the perinuclear region of the cytoplasm (7.78%), and intracellular organelle parts (7%). The top 10 biological processes in our analyses unveiled that Histone H3-K9 demethylation, chromatin modification, transcription, DNA-dependent regulation of transcription from RNA polymerase II promoter, and negative regulation of NF-κB transcription factor activity, which usually participates in transcriptional regulation, were likely to be critical in the maintenance of latency and are worth further exploration (Supplementary Fig. 1B). Furthermore, these ten genes are involved in DNA binding, transcription regulator activity, receptor signaling complex scaffold activity, and G-protein-coupled receptor activity (Supplementary Fig. 1C). In addition, the top 10 KEGG pathways are shown in Supplementary Fig. 1D and include regulation of Ras homolog enriched in brain (Rheb) GTPase activity by the TRIF complex, activation of RIP1, TAK1, RhO GTPase signaling, p75 NTR, AMPK, PI3K/AKT activation, and inhibition of TSC complex formation by protein kinase B. HIV survival is dependent on its ability to exploit host cell machinery for replication and dissemination and to circumvent cellular processes that prevent its growth. Investigating the link between these biological activities and HIV-1 latency will provide insight into the interaction between cellular metabolism and HIV latency.

### *TSC1* and *DEPDC5* are likely to be involved in HIV-1 latency

To determine the most enriched genes in activated C11 cells, we calculated scores for all genes by using a previously reported scoring system^39^. The highest scoring gene was N6AMT2, as shown in Supplemental Table S1. The log2 value relative to the fold change of the negative control was calculated to obtain the ranked position of N6AMT2 and to calculate the Ss value. Because three sgRNAs target N6AMT2, Sg = SS1 + SS2 + SS3. N6AMT2 was highly enriched in three independent biological replicates of this screening. The cumulative score (Ts) for each gene was calculated as Ts = (SgI + SgII + SgIII)^3^. We then plotted the scoring data in Fig. 1d, and 25 gene targets showed high enrichment over the background. SUV39H1, a lysine methyl transferase, was identified as a regulator of HIV latency in our screening (Fig. 1d), which was consistent with a previous report^40^. It is known that SUV39H1 mediates the trimethylation of lysine 9 at histone H3 (H3K9me2/3) to stabilize constitutive heterochromatin structures by recruiting HP1 chromodomain-containing adaptor proteins^40^. In addition, two inhibitory molecules, *TSC1* and *DEPDC5*, were also identified (Fig. 1d). However, the mechanisms of how *TSC1* and *DEPDC5* regulate HIV-1 latency need to be thoroughly investigated.

### Knockdown/out of *TSC1* or *DEPDC5* results in HIV-1 reactivation in latently infected cells

**Fig. 2:**
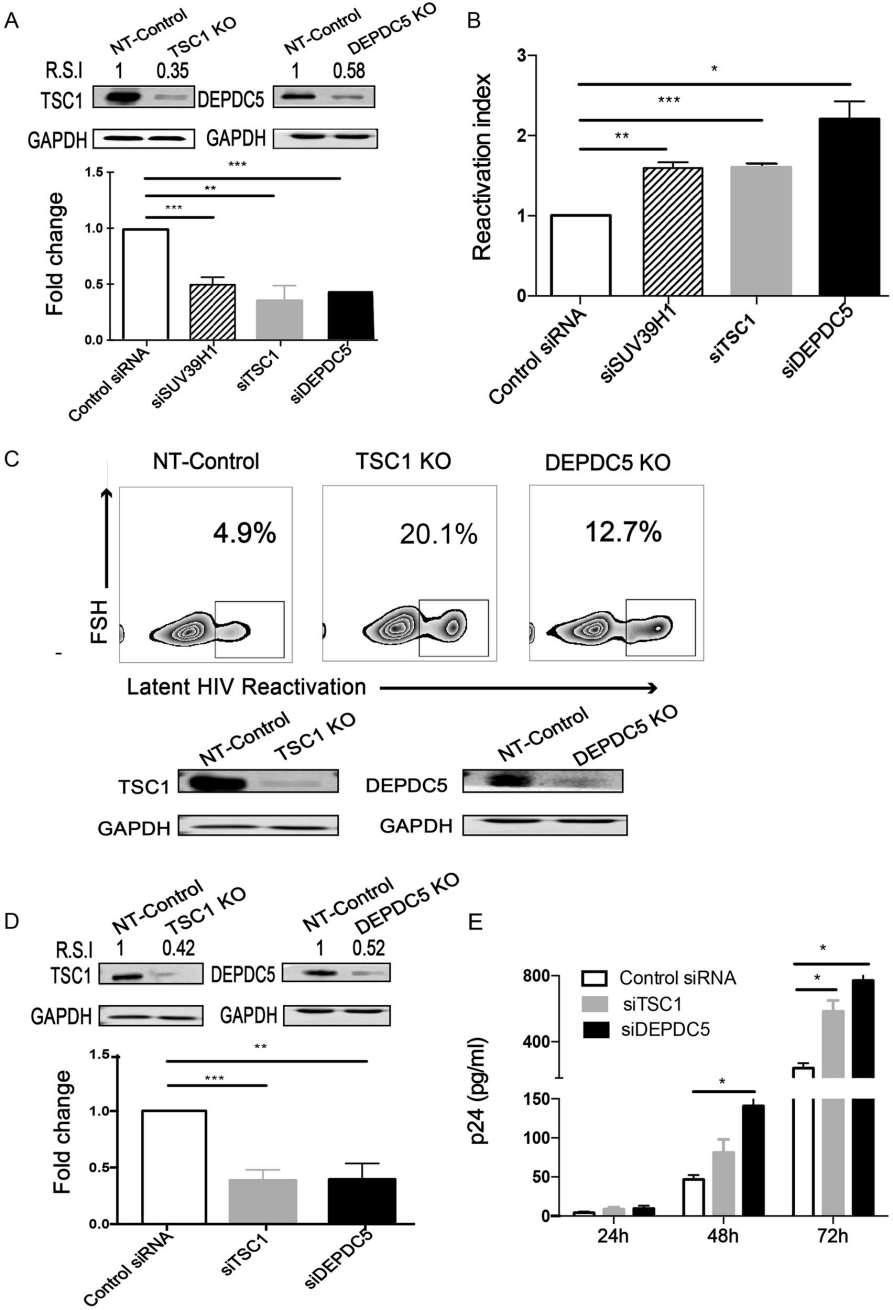
Knockdown/out of TSC1 or DEPDC5 results in HIV-1 reactivation in latently infected cells. **a** Knockdown efficacy of TSC1 (left) and DEPDC5 (right) in C11 cells was determined by WB. The representative pattern is shown in upper panel. The siRNA knockdown (%KD) was also detected by qPCR (bottom panel). **b** Fold activation of DEPDC5, TSC1, and SUV39H1 knocked down in C11 cells without co-stimulation. Analysis was conducted on 5 × 10^5^ live cells per condition, and all experiments were independently repeated at least three times. **c** Constructed stable knockout of either TSC1 or DEPDC5 in C11 cell lines. NT-control indicates non-targeting sgRNA knockout in C11 cell lines. TSC1 or DEPDC5 knockout led to spontaneous latency reversal compared with the NT-control (upper panel). Knockout efficiency of TSC1 (left) and DEPDC5 (right) in C11 cells was detected by WB. The representative pattern is shown (bottom panel). **d** Knockdown efficacy of TSC1 (left) and DEPDC5 (right) in U1 cells was determined by WB. The representative pattern is shown in the upper panel. Knockdown efficiency of siRNA in the U1 cell line was also detected by qPCR (bottom panel). **e** Transient knockdown of TSC1/DEPDC5 in U1 cells resulted in increased p24. The cell supernatants were collected at three time points (24, 48, and 72 h). Data represent the mean ± SD of three independent experiments. The significance of differences between groups was determined using the two-tailed Student’s *t*-test (***P < 0.005; **P < 0.01, *P < 0.05)

To confirm the effect of *TSC1* and *DEPDC5* on HIV-1 latency, we transiently knocked down *TSC1* or *DEPDC5* by small interfering RNA (siRNA) (Fig. 2a) and observed a significant HIV-1 latency reactivation indicated by increased GFP^+^ cells in the C11 cell line (Fig. 2b). We then further corroborated the above observation with a knockout model and established cell lines with a stable knockout of either *TSC1* or *DEPDC5* in the C11 cell line (Fig. 2c, bottom panel), using a non-targeting (NT) sgRNA knockout cell line as the control. We found that *TSC1* and *DEPDC5* knockout led to the reactivation of HIV-1 latency compared with the NT-control (Fig. 2c, upper panel). To further confirm the effect of *TSC1* or *DEPDC5* on HIV-1 latency, we performed knockdown experiments in the U1 cell line (Fig. 2d), a monocyte cell line containing a single copy of latent HIV-1 provirus^41^. We found that *TSC1*/*DEPDC5* knockdown dramatically activated HIV-1 expression, as indicated by the expression of p24 in the culture supernatant, and p24 expression peaked (864 pg/ml) at 72 h post knockdown (Fig. 2e). Taken together, we demonstrated that *TSC1* and *DEPDC5* are likely factors that maintain HIV-1 latency.

### The mTOR pathway is associated with HIV-1 latency

**Fig. 3:**
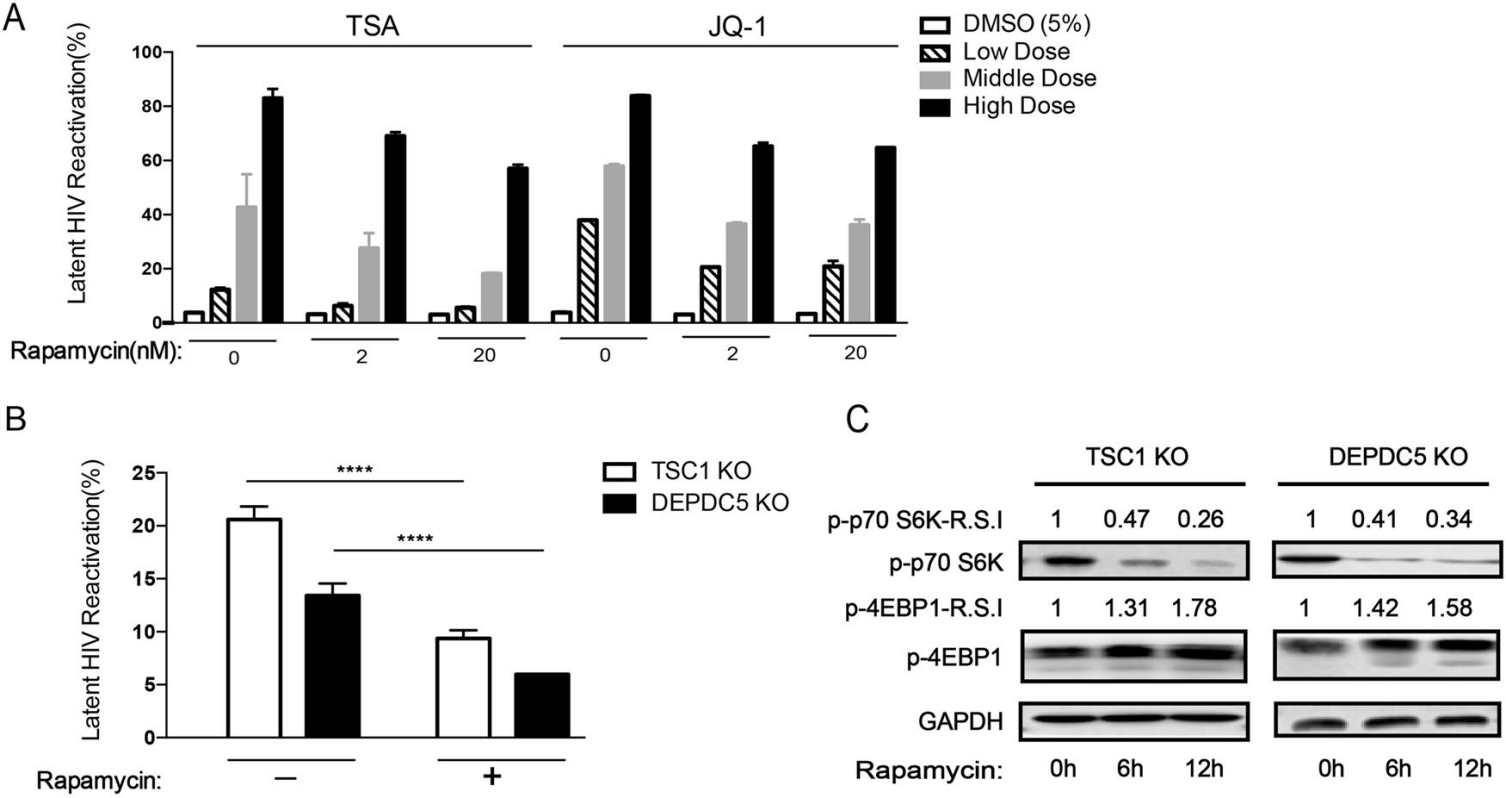
The mTOR pathway is associated with HIV latency. **a** C11 cell pretreatment with rapamycin (2/20 nM) for 6 h to reactivate HIV in the presence of increasing concentrations of TSA/JQ1. Reactivation of HIV was assessed by measuring GFP by flow cytometry. Data represent the mean ± SD. **b** The percentage of GFP^+^ cells upon rapamycin (2 nM) treatment in TSC1/DEPDC5 knockout C11 cells. Data represent the mean ± SD of triplicate values, representative of two independent experiments. The significance of differences between groups was determined using the two-tailed Student’s *t*-test (****P < 0.001). **c** Rapamycin stimulates phosphorylation of S6 and 4EBP1 in TSC1/DEPDC5 knockout C11 cells. Cells were untreated or pre-treated with rapamycin (2 nM) for 6 h/12 h. Images shown are representative of three independent experiments

Both *TSC1* and *DEPDC5* are suppressors of mTORC1 and may maintain HIV-1 latency by downregulating mTORC1 in C11 cells. To verify the role of the mTOR pathway in HIV-1 latency, we established an HIV-1 latency reactivation–inhibition model. We first treated C11 cells with LRAs (Trichostatin A (TSA) and JQ1) to cause reactivation of the latent HIV-1 and then administered the mTOR inhibitor rapamycin to examine whether reactivation could be inhibited. TSA is a known histone deacetylase (HDAC) inhibitor and has been proposed as an agent that can enhance reactivation^42^. In addition, JQ1 is a small molecule inhibitor of the BET family bromodomains that regulates HIV transcription by inhibiting the interaction with P-TEFb^43^. Interestingly, reactivation of the HIV-1 latent virus in C11 cells by LRAs was suppressed by rapamycin in a dose-dependent manner (Fig. 3a), suggesting that downregulating mTORC1 promotes HIV-1 latency. In addition, the increased reactivation of HIV-1 in *TSC1*/*DEPDC5* knockout C11 cells was largely blocked by rapamycin treatment (Fig. 3b) by attenuating S6 but enhancing downstream 4EBP1 pathways in a time-dependent manner (Fig. 3c). These results indicate that *TSC1* and *DEPDC5* do indeed regulate HIV latency through regulation of the mTORC1 pathway.

### *TSC1* and *DEPDC5* negatively regulate the AKT-mTOR pathway

**Fig. 4:**
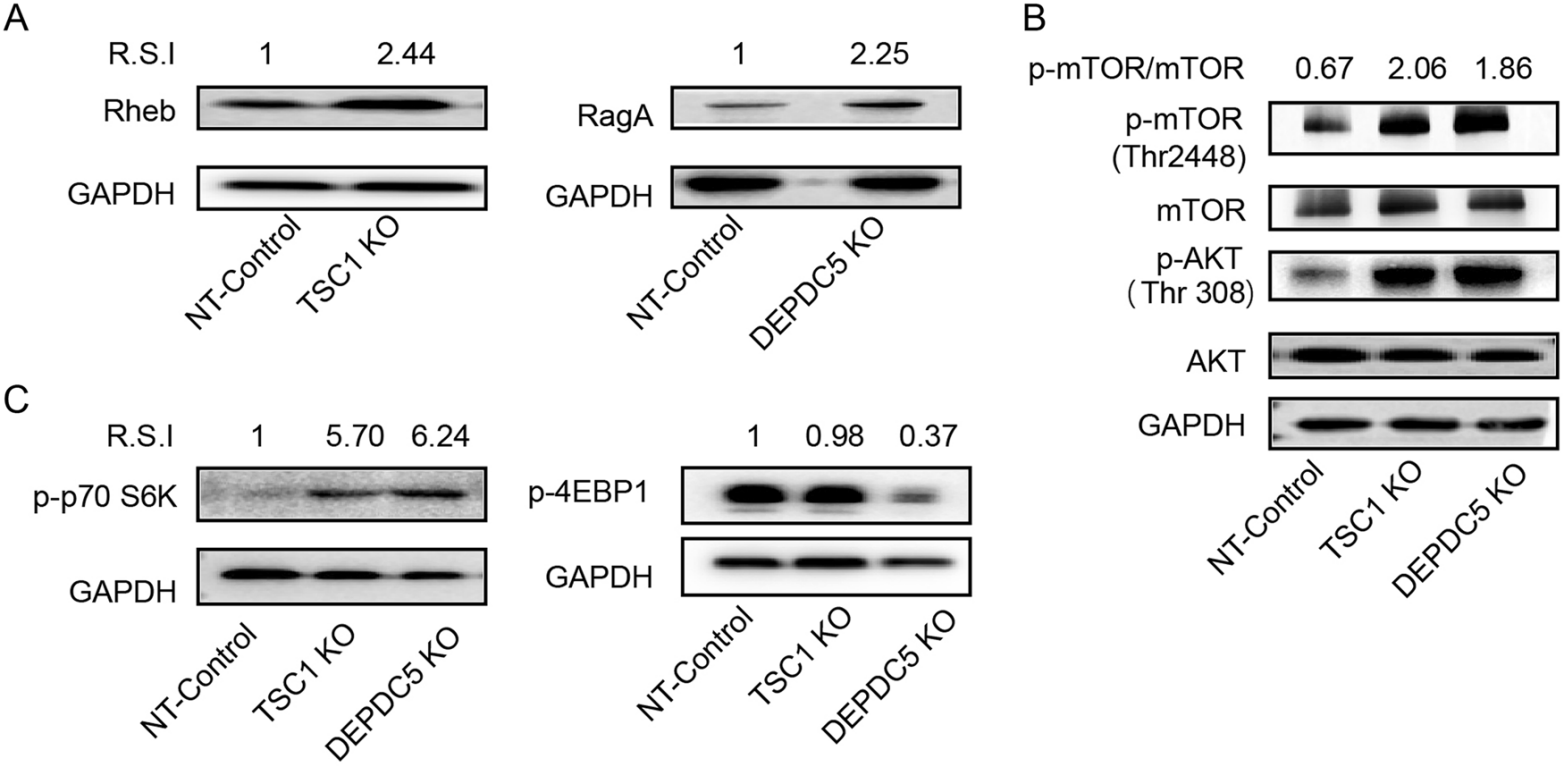
TSC1 and DEPDC5 negatively regulate the AKT-mTOR pathway. **a** Left: WB analyses of Rheb expression in the TSC1 knockout cell line. Right: WB analyses of RagA expression in the DEPDC5 knockout cell line. **b** The effects of DEPDC5 and TSC1 on upstream AKT and downstream mTOR. Phosphorylation of AKT was monitored by the anti-phospho-AKT (S308) antibody. Phosphorylation of mTOR was detected by the anti-phospho-mTOR (S2448) antibody. **c** Graph showing the effect of DEPDC5 or TSC1 knockout on S6K (left) and 4EBP1 (right) phosphorylation. Images shown are representative of three independent experiments

As *TSC1* functions as a GTPase-activating protein (GAP) for the Rheb GTPase^44^ and *DEPDC5* acts as a GAP for RagA/B, a component of the GATOR1 complex that promotes mTORC1^45^, we determined whether *TSC1* and *DEPDC5* knockout could trigger a downstream cascade in C11 cells. Indeed, *TSC1* knockout resulted in a 2.44-fold increase in Rheb, while *DEPDC5* knockout led to a 2.25-fold increase in RagA (Fig. 4a). Importantly, the absence of both *TSC1* and *DEPDC5* caused enhanced phosphorylation of mTORC1 (phosphor-mTOR-Thr 2448) and resulted in a 2–3-fold increase in the p-mTOR/mTOR ratio relative to the control (Fig. 4b). Both *TSC1* and *DEPDC5* knockout resulted in a five- to sixfold increase in S6 phosphorylation (Fig. 4c, left panel). However, while *DEPDC5* knockout resulted in a threefold reduction of 4EBP1, *TSC1* knockout induced little to no downregulation of 4EBP1 (Fig. 4c, right panel). Unexpectedly, phosphorylation of the upstream gene AKT (phosphor-AKT-Thr308) was also upregulated in *TSC1*/*DEPDC5* knockout cells (Fig. 4b), which may result from feedback causing upregulation of mTORC1 in the absence of *TSC1* or *DEPDC5*. Overall, both *TSC1* and *DEPDC5* negatively regulate the mTORC1 pathway, and removal of either of these two genes triggers the activation cascade of the mTORC1 pathway.

## Discussion

Current antiretroviral therapy for HIV-1 infection effectively suppresses but does not eradicate HIV-1^1^. Achieving viral eradication has proven difficult due to both the intrinsic biological properties of latent proviruses and the nature of their cellular hosts^46^. In an effort to discover the host factors required for HIV latency, including the establishment and maintenance of HIV latency, genome-wide genetic and functional studies are crucial. However, the use of large-scale loss-of-function screening to identify functionally relevant genes has been hampered by technical limitations, including the inability of shRNAs to effectively block gene biogenesis and function. Here, we are the first to utilize a genome-wide CRISPR screen containing 120,000 gRNAs targeting more than 19,000 human genes to identify host factors associated with HIV latency. We identified 257 enriched genes, relevant to the reactivation of latent HIV-1, which are involved in chromatin organization/modification and transcriptional regulation. The relevancy of this approach was further evidenced by the inclusion of genes previously reported as HIV-1 latency-promoting genes, such as SUV39H1^40^.

New approaches aimed to reverse HIV-1 latency include interfering with the development, homeostasis, activation, and differentiation of T cells^47,48^. It was reported that HIV-1 infection of activated T cells at stages after the reverse transcription phase was suppressed through blocking the cellular mTOR^49,50^. In addition, mTOR inhibitors abrogated Tat-independent and Tat-dependent transactivation of the HIV-1 promoter and reduced global CDK9 phosphorylation in CD3/CD28-stimulated CD4 T cells from HIV-1 uninfected donors^29^. Rapamycin represses basal transcription of the HIV-1 LTR without significantly affecting Tat-mediated transactivation^51^. Furthermore, lower frequencies of lymphocytes containing HIV DNA were observed in rapamycin-treated HIV-infected kidney transplant recipients than in patients treated with other immunosuppressive drugs^52^. Altogether, suppression of the AKT-mTORC1 pathway may represent an effective approach to promote HIV-1 latency. According to our genome-wide CRISPR screening, two mTORC1 natural inhibitors, *TSC1* and *DEPDC5*, scored high as host factors for promoting HIV-1 latency.

As previously reported, activation of AKT leads to *TSC1* inhibition and Rheb conversion to an active GTP-bound state to activate mTORC1^53^. Activation of Rheb is essential for mTORC1 signaling, which requires the presence of an amino acid branch^54^. *DEPDC5*, as a GAP for the Rags 1 (GATOR1) complex, functions as an inhibitor of the amino acid-sensing branch of the mTORC1 pathway^55^. Activated mTORC1 phosphorylates S6K1 and inhibits phosphorylation of 4EBP1^53^ to promote translation of mRNA^26,56^. Therefore, *TSC1* and *DEPDC5* may maintain HIV-1 latency by suppressing the AKT-mTORC1 pathway. Two lines of evidence support this hypothesis. First, *TSC1* or *DEPDC5* knockout triggers the AKT-mTORC1 pathway cascade and results in enhanced HIV-1 reactivation. Second, the enhanced reactivation of latent HIV-1 is largely blocked by the mTORC1 inhibitor rapamycin in a dose-dependent manner.

The cellular nucleus, cytoplasm and lysosome are important sites for maintaining HIV latency. Most mechanisms that lead to induction and maintenance of HIV latency operate in the nucleus by either chromatin remodeling or epigenetic and transcriptional regulation^2,11,57–59^. The cytoplasmic site is likely to engage in signaling transduction from the outside of cells into the nucleus to maintain the quiescence of cells^1,60,61^. Meanwhile, post-transcriptional regulatory mechanisms all occur in the cytoplasm^62^. For example, five cellular microRNAs (miR-28, miR-125b, miR-150, miR-223, and miR-382) bind to the 3′-untranslated region of HIV-1 mRNAs and can inhibit the translation of almost all HIV-1-encoded proteins, including Tat and Rev, the key regulators of viral gene transcription and viral RNA translocation^63^. Furthermore, the lysosome may also affect HIV latency by controlling autophagy. The autophagosomes fuse with lysosomes to form autolysosomes in which the sequestered material is degraded and then recycled^64^. Theoretically, autophagy restricts HIV-1 reactivation by selectively degrading the viral transactivator Tat, a viral protein that could be antagonized by Vif in the early stage of autophagy^65,66^. Accordingly, more than 30% of our CRISPR/Cas9 enriched genes are localized in the nucleus, and the main biological functions of these include histone H3-K9 demethylation, NF-κB transcription factor activity, and regulation of transcription from RNA polymerase II promoter, suggesting that chromatin remodeling and epigenetic and transcriptional regulation are likely involved in the maintenance of latency. In our study, we identified 257 enriched genes relevant to the reactivation of latent HIV-1 and observed almost 100% activation of latency in those experiments (4th sorting round). Meanwhile, only 20.1% and 12.7% activation was observed when *TSC1* and *DEPDC5* were knocked out, respectively, indicating that it is likely that *TSC1*, *DEPDC5*, and SUV39H1 only have partial roles in the activation of latently infected HIV-1 and that targeting similar genes will lead to more efficient activation of latency. GO analyses showed that multiple pathways other than the mTOR pathway are associated with HIV latency reactivation, including some epigenetic factors. We rationalized that targeting more genes to affect other pathways, such as epigenetic regulation pathways, may better control HIV-1 latency. The mechanism of other differential genes affecting HIV-1 latency needs to be studied further.

In addition, we showed that *TSC1* and *DEPDC5* maintain HIV-1 latency by negatively regulating the AKT-mTORC1 pathway in cell lines and that this working mechanism, similar to rapamycin, does not rely on the integration sites. However, it remains unknown whether the downstream regulation mechanism of the AKT-mTORC1 pathway on latency depends on integration sites. Although AKT-mTORC1 regulates metabolism, the cell lines/effective T cells are hyper glycolytic, memory/resting T cells harboring the HIV genome rely on fatty acid oxidation to produce energy^67,68^, and it is therefore possible that *TSC1* and *DEPDC5* are unable to have a similar role in primary T cells in vivo. In addition, our experiments in vitro showed that the removal of suppression of *TSC1* and *DEPDC5* only resulted in ~20% latency reactivation though their integration site and metabolism status are identical, suggesting that regulatory factors other than integration site and metabolism status are also involved in the latency reactivation. Overall, we speculate that the latency reactivation in vivo is likely to be more complicated by heterologous integration sites, engaging multiple organs and cell subsets in various differentiation stages and metabolism status, and a variety of epigenetic regulatory mechanisms.

Overall, our data validate the hypothesis that *TSC1* and *DEPDC5* maintain HIV-1 latency by suppressing the AKT-mTORC1 pathway activity and, thereby, hinder the initiation of transcriptional translation to maintain the latency status of HIV-1. It remains to be determined how these factors coordinate with other factors in regulating HIV-1 latency and whether they represent the universal mechanism for HIV-1 latency. It will be interesting to test whether *DEPDC5* or *TSC1* agonists could be used as synergistic agents with current “shock” components to further improve the reactivation of latent HIV-1. On the other hand, the ‘permanent silencing’ approach could be considered as a suitable and practical cure strategy^69^, and our data also suggest that *TSC1* and *DEPDC5* might be used in metabolic inhibition to “block and lock” the latent reservoir. The continued search for additional regulators of HIV-1 latency remains a key challenging task.

## Materials and methods

### HIV-1 latent cell lines and reagents

The HIV-1 latent infection model C11 cell line was developed in-house and contains a single integrated latent HIV-GFP reporter genome^37,38^. U1 cells (a monocytic cell line) were chronically infected with the Tat-deficient HIV virus obtained from the AIDS Research and Reference Reagent Program. C11 or U1 cells were propagated in RPMI 1640 medium, supplemented with 10% fetal bovine serum (FBS) (HyClone Laboratories, Logan, UT), penicillin (100 IU/ml), and streptomycin (100 g/ml) (Life Technologies/BRL, Carlsbad, CA) at 37 °C with 5% CO_2_. HEK293T cells were cultured in Dulbecco’s modified Eagle’s medium (HyClone Laboratories) supplemented with 10% FBS, penicillin (100 IU/ml), and streptomycin (100 g/ml) at 37 °C with 5% CO_2_. Phospho-mTOR (Ser2481) monoclonal antibody (mAb), mTOR (7C10) rabbit mAb, phospho-AKT (Thr308) mAb, AKT mouse mAb, RagA (D8B5) rabbit mAb, Rheb (E1G1R) rabbit mAb, *TSC1* rabbit mAb, phospho-p70 S6 kinase (Thr389) (108D2) rabbit mAb, and phospho-4E-BP1 (Thr37/46) (236B4) rabbit mAb were from Cell Signaling Technology (Danvers, MA). DEPD5 antibody (C-term) was from Abgent (Suzhou, China). Glyceraldehyde 3-phosphate dehydrogenase (GAPDH) loading control mAb was from Thermo Fisher (Waltham, MA). Puromycin was from InvivoGen (San Diego, CA).

### Generation of genome-wide mutant libraries and screening

The Genome-Wide CRISPR Knockout (Gecko) v2.0 library containing 120,000 gRNA sequences was used. Lentiviruses were produced by co-transfecting 293T cells and then concentrating 1000 × to increase the viral titer. A total of 1 × 10^8^ C11 cells were infected at a low multiplicity of infection (MOI) (0.1~0.3), to ensure that most cells received only one viral construct with a high probability. Twenty-four hours after transduction, cells were selected with 2 μg/ml puromycin for at least 2 weeks and harvested. The proportion of GFP-positive C11 cells increased after infection with CRISPRV2.0. GFP-positive cells were sorted for 4 rounds and enriched genes. gDNA was extracted using the Zymo Quick-gDNA Miniprep kit (Zymo Research, Orange, CA).

PCR was performed with the following primers: 5′-TCTTTCCCTACACGACGCTCTTCCGATCTTAACTTGAAAGTATTCTTGGCTTTATATATCTTGTGGAAAGGACGAAACACCG -3′ and 5′-CTGGAGTTCCTTGGCACCCGAGAATTCCAATTTTTCAAGTTGATAACGGACTAGCCTTATTTTAACTTGCTATTTCTAGCTCTAAAAC-3′ annealing up- and downstream of the gRNA sequence.

### Analysis of genome-wide screening results

The PCR products containing gRNA sequences were integrated into the genomes of the cells and subjected to parallel sequencing using a HiSeq 3000 (Illumina). Reads for each gRNA were normalized to counts per million total reads, and all counts below 5 were set to 5 per million reads to remove low read noise. The enrichment of each individual gRNA was calculated compared with the distribution of the log2 enrichment values of the negative control gRNA. The previously reported scoring system was applied^39^. Specifically, individual sgRNAs with more than 0.0005% abundance were given a score (Ss) for a given screen replicate, where Ss = log10(10,000/Ranked position based on abundance). Each gene was given a score for a screen (Sg) based on the sum of the scores of each of the six sgRNAs targeting that gene if any sgRNAs for that gene met the above criteria (Sg = SS1 + SS2 + SS3 + SS4 + SS5 + SS6). The cumulative score Ts for each gene was calculated as Ts = (SgI + SgII + SgIII) ^ (Number of screens with an Sg score).

### Flow cytometry-monitored GFP expression

C11 cells were centrifuged and resuspended in 1E7/100 μl RPMI + 1% FBS. Stained cells were sorted using BD FACSAria and analyzed using BD LSRFortessa. FlowJo software (FlowJo LLC, Ashland, OR) was used to perform the flow cytometry analysis.

### RNA isolation and RT-PCR for knockdown efficiency

Total RNA was isolated from CRISPR-targeted cells using a Direct-zol RNA Miniprep kit (Zymo Research), and 1 μg of total RNA was reverse transcribed using a reverse transcription system product with oligo(dT)15 primer (Promega, Fitchburg, WI). RReverse transcription-quantitative PCR (RT-qPCR) was performed using SYBR Green PCR Master Mix (Thermo Fisher Scientific, Waltham, MA).

*DEPDC5* primers: 5′-GCTGTGAATGGTTTCCTTGCT-3′ and 5′-CTGTCGAATTGAGGCTCGGT-3′. *TSC1* primers: 5′-ACCAGCCCTTATGCTGACAC-3′ and 5′-TTATCAGCCGTGTCGATGGG-3′. GAPDH primers: 5′-ACGGATTTGGTCGTATTGGG-3′ and 5′-ATCTCGCTCCTGGAAGATGG-3′. The PCR procedures were as follows: 95 °C for 5 min, followed by 40 cycles at 95 °C for 15 s and 60 °C for 30 s. Quantified mRNA was normalized to GAPDH as a control. The relative expression of mRNA was determined by the 2^ΔΔCT^ method according to the manufacturer’s instructions (Eppendorf, Hamburg, Germany).

### Validation of sgRNA screened genes

Scrambled siRNA (control siRNA) and specific siRNA (*TSC1*/*DEPDC5*/SUV39H1) were used for transient silencing of the expression of genes. All oligonucleotides were synthesized by RiboBio (RiboBio Co. Ltd. Guangzhou, China) and delivered to 1 × 10^6^–3 × 10^6^ C11 cells or U1 cells at a final concentration of 1 × 10^6^/200 pmol oligonucleotides. The cells were cultured in a 24-well plate for 48 h or 72 h with 2 ml of fresh culture medium (RPMI 1640 medium supplemented with 10% FBS) per well and harvested, and the transfection efficiency was confirmed by qPCR.

### CRISPR/Cas9-mediated *TSC1*/*DEPDC5* knockout

*TSC1*/*DEPDC5*-targeting sgRNAs were cloned into the LentiCRISPR v2.0 vector (Addgene, 52961). C11 cells were infected with lentivirus at an MOI of 0.3 and then selected using 2 μg/ml puromycin for 14 days. The resulting knockout efficiency was analyzed using Sanger DNA sequencing. Single knockout cells were sorted by flow cytometry, and knockout efficiency was detected by western blot (WB) analysis.

### Reversal of HIV latency with drugs

Latent C11 cells were pre-treated with 2 or 20 nM of the mTOR inhibitor rapamycin for 6 h and then plated at 100,000 cells/well in round-bottom 96-well plates in the presence of reactivation drugs (TSA or JQ1) or dimethyl sulfoxide vehicle. After 21~24 h of drug treatment, cells were fixed in a final concentration of 2% paraformaldehyde. Flow cytometry analysis was performed using a BD FACSAria system. *TSC1* knockout cells and *DEPDC5* knockout cells were treated with rapamycin (2 nM), and GFP was measured by flow cytometry. Cells were collected at 6 h and 12 h, and WB analysis was used to detect the phosphorylation of S6 and 4EBP1.

### AKT-mTOR pathway activities assessed by WB analysis

A total of 1 × 10^6^ cells were washed, collected in 25 μl of phosphate-buffered saline, and lysed in 75 μl of loading buffer (TransGen Biotech, Beijing, China). Equal amounts of transfected cell lysates were loaded onto an 8%~15% SDS-PApolyacrylamide gel electrophoresis gel, separated at a voltage of 70 V/110 V, and electrophoretically transferred to polyvinylidene fluoride (PVDF) membranes at a constant current. PVDF membranes were blocked with 5% bovine serum albumin in Tris-buffered Saline with Tween 20 (TBST), incubated with primary antibodies (Abs), and further incubated with horseradish peroxidase-conjugated secondary Abs.

### Statistical analyses

The data obtained from the different treatments in the same sample were evaluated using the two-tailed Student’s *t*-test. RT-qPCR data that were ten-fold higher or lower than the mean values were identified as outliers and excluded from the analyses. All data were processed using GraphPad Prism, version 5.0 (GraphPad Software, San Diego, CA). A *P*-value of 0.01 was defined as statistically significant.

### Data availability

The raw sequencing data were deposited in the NCBI GEO database (https://www.ncbi.nlm.nih.gov/geo) under the accession code GSE109808.

## Electronic supplementary material

Supplementary Figure S1

Supplementary Figure S2

Supplementary table S1
