# Supplementary figures and images for: TSC1 and DEPDC5 regulate HIV-1 latency through the mTOR signaling pathway
Source: Emerg Microbes Infect. 2018 Aug 8;7:138. doi: 10.1038/s41426-018-0139-5 (PMC6081400; doi:10.1038/s41426-018-0139-5)

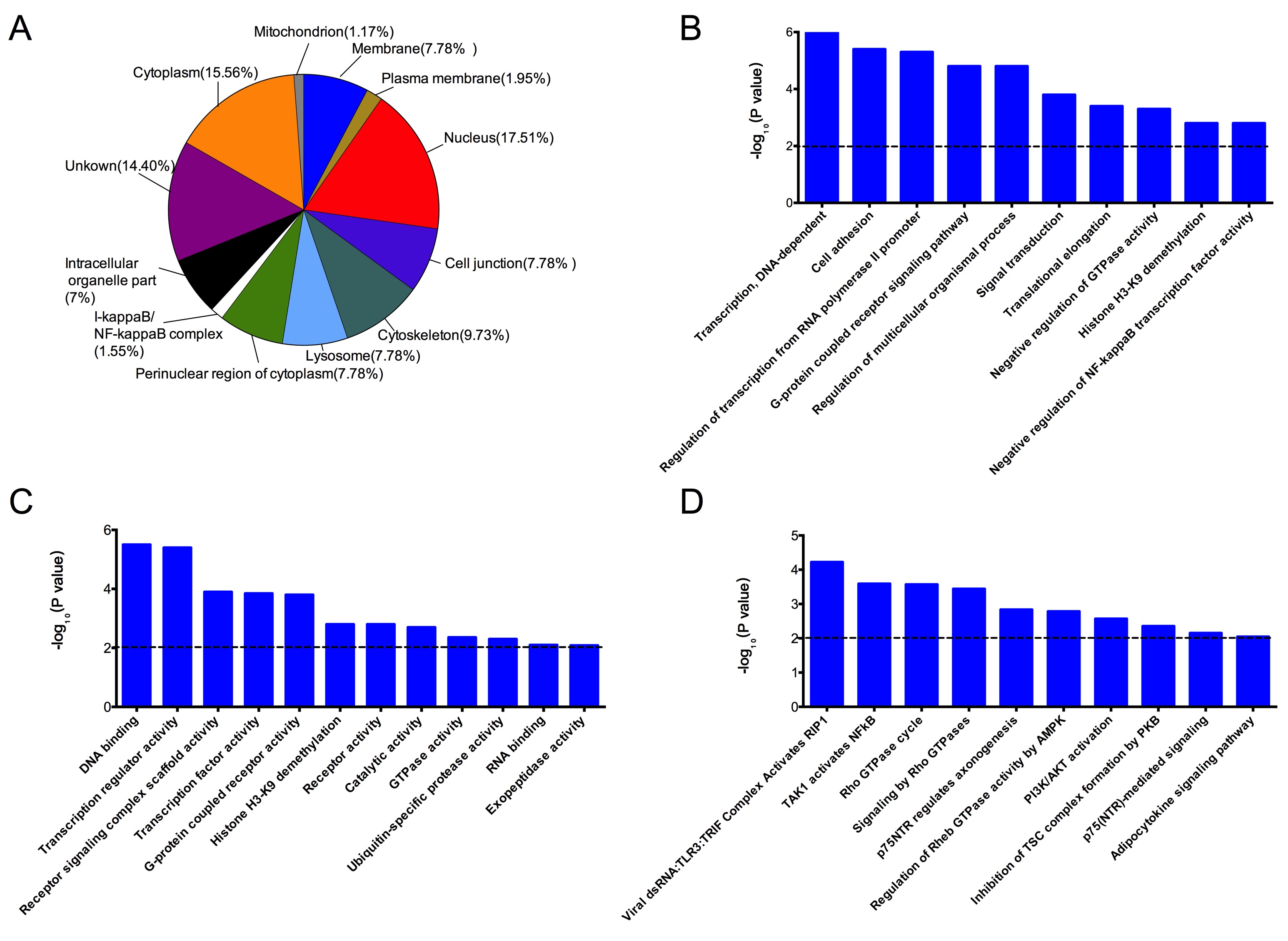

Supplement: Supplementary file 1 — Supplementary Figure S1 [file 41426_2018_139_MOESM1_ESM.jpg]

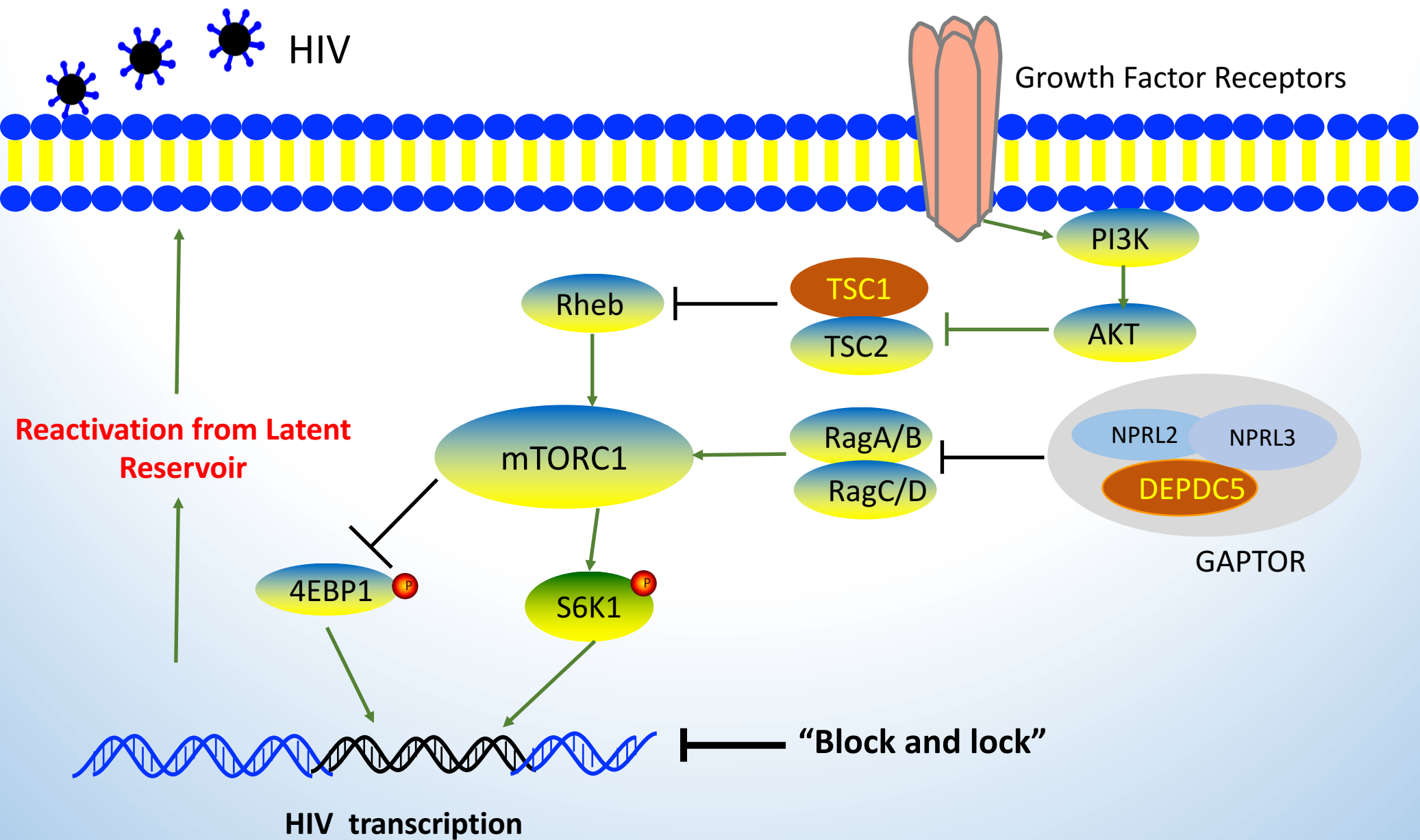

Supplement: Supplementary file 2 — Supplementary Figure S2 [file 41426_2018_139_MOESM2_ESM.pdf]
