# Supplementary material for: TSC1 and DEPDC5 regulate HIV-1 latency through the mTOR signaling pathway
Source: Emerg Microbes Infect. 2018 Aug 8;7:138. doi: 10.1038/s41426-018-0139-5 (PMC6081400; doi:10.1038/s41426-018-0139-5)
Supplement: Supplementary file 3 — Supplementary table S1 [file 41426_2018_139_MOESM3_ESM.pdf]

Refseq ID	Gene Name	Ts
HGLibA_30968	N6AMT2	453.3003446
HGLibB_07970	CCL16	316.8411609
HGLibA_51810	TSC1	300
HGLibB_12982	DEPDC5	270.2891973
HGLibB_46468	SPATA6L	258.0393579
HGLibB_34629	OSCP1	249.5221073
HGLibB_18545	GALNT7	227.8862701
HGLibB_47802	SUV39H1	209.0550876
HGLibA_31818	NFKB2	198.8275739
HGLibB_30264	MT3	191.376877
HGLibA_40524	RBM27	181.2209337
HGLibB_45096	SLC43A1	178.28905
HGLibA_13200	DHX40	174.1471049
HGLibA_33516	OR10H4	172.4815195
HGLibA_24337	KCNK9	155.2284141
HGLibB_28478	MBOAT2	143.9702665
HGLibA_26042	LARP7	139.8700592
HGLibB_38094	PPP2R1B	129.9987169
HGLibA_08691	CDH8	125.4505066
HGLibB_13859	DPP8	123.3724419
HGLibA_42239	RRAS2	122.2487527
HGLibB_46122	SORBS2	119.0826093
HGLibA_33557	OR10X1	108.9289581
HGLibB_00076	AASDH	107.5535144
HGLibA_14722	EIF2AK1	101.0683773
HGLibB_24700	KIAA1279	99.57680067
HGLibA_48296	TANGO2	97.20923461
HGLibA_54058	WDR27	89.09871466
HGLibA_20840	HAPLN1	87.25964819
HGLibA_61385	hsa-mir-496	85.6793298
HGLibB_16326	FAM188B	84.26749107
HGLibA_31871	NGF	78.53958166
HGLibB_47525	STRBP	78.17843282
HGLibA_64282	hsa-mir-922	77.80134826
HGLibA_28073	MAN2A2	73.67389173
HGLibA_21115	HELLS	71.11343182
HGLibA_46264	SOX4	70.57439519
HGLibB_46177	SOX12	70.50646157
HGLibA_26146	LCE4A	64
HGLibA_31555	NDUFS4	61.50617233
HGLibB_38732	PRR14L	60.65051375
HGLibA_57225	hsa-mir-1183	60.61494794
HGLibA_61801	hsa-mir-527	55.94533673
HGLibA_46002	SNTB2	55.82752158

HGLibB_10067	CLN3	55.72551683
HGLibA_30854	MYO5B	54.72951637
HGLibB_10719	COPS7B	54.35210961
HGLibB_37420	POFUT1	53.81883868
HGLibB_43019	SCUBE2	53.37945816
HGLibB_18091	FTH1	52.72324244
HGLibB_20495	GTF2A1L	51.99070579
HGLibA_19880	GPR125	49.64269862
HGLibA_55512	ZNF160	49.27712465
HGLibA_46701	SPINK6	49.03766457
HGLibA_21913	HOXA5	48.82370566
HGLibB_18545	GALNT7	48.28847878
HGLibB_21508	HIST2H2BF	46.62121265
HGLibA_24881	KIF19	45.03916952
HGLibA_08310	CD247	43.86105761
HGLibA_32978	NUDT4	43.58719613
HGLibA_46110	SNX9	41.52123746
HGLibB_20440	GSTK1	40.69585067
HGLibB_36851	PLA2G3	40.02864758
HGLibB_33164	OASL	39.81362551
HGLibB_18373	GABRP	37.28030769
HGLibA_63787	hsa-mir-7107	37.10655477
HGLibA_41179	RHOBTB3	36.67726271
HGLibB_23243	INCA1	36
HGLibA_57440	hsa-mir-1258	35.01342897
HGLibB_03327	ASMTL_Y	34.12472877
HGLibA_47611	STUB1	33.31729189
HGLibB_30845	MYOG	33.19581135
HGLibB_22698	IFNL4	32.57834075
HGLibB_46062	SOAT2	32.01586896
HGLibB_34631	OSCP1	31.26778029
HGLibA_19280	GLMN	30.76469429
HGLibB_08642	CDH17	30.13411608
HGLibA_30546	MUC4	29.62072786
HGLibA_45936	SNPH	29.13775634
HGLibA_33514	OR10H4	28.68203802
HGLibB_20252	GRIK1	28.30527991
HGLibA_36575	PIGP	28.25405918
HGLibB_48985	TET3	27.88780696
HGLibA_08820	CDKN1A	27.84192004
HGLibA_19767	GPC3	27.45317255
HGLibB_47106	SSR4	26.72945117
HGLibB_11949	CXorf66	26.62194751
HGLibB_50551	TMPRSS11A	25.75769568
HGLibA_35294	PAX1	25.54783986

HGLibB_03209	ASB12	25.00186158
HGLibB_51090	TPMT	24.89710652
HGLibB_18503	GALNT1	24.63109227
HGLibA_40993	RGAG1	24.37438535
HGLibB_01621	ALG1L	24.26371009
HGLibA_04891	BTAF1	23.65457142
HGLibA_36419	PHLDA2	23.42978976
HGLibA_42239	RRAS2	23.21187891
HGLibB_22949	IL15	23.00046514
HGLibA_17624	FLJ25363	22.79520366
HGLibB_25582	KRTAP10-7	22.59577574
HGLibB_34103	OR52B4	22.21326115
HGLibA_12035	CYFIP2	22.02964602
HGLibA_41562	RNF167	21.85080381
HGLibB_02046	ANKRD26	21.67651353
HGLibB_16044	FAM122A	21.5065688
HGLibB_23436	IPO5	21.34077659
HGLibA_16904	FASTKD2	21.17895616
HGLibB_27577	LTV1	21.02093799
HGLibB_45266	SLC6A2	20.86656291
HGLibB_19964	GPR19	20.71568127
HGLibA_28459	MB21D2	20.56815224
HGLibA_05115	C11orf1	20.28262865
HGLibA_42121	RPS27	20.14439065
HGLibB_18600	GAREM	20.00901736
HGLibB_02000	ANKRD13B	19.87640302
HGLibB_22490	ICT1	19.7742976
HGLibB_27894	MAGEB16	19.49413781
HGLibB_06252	C3orf62	19.37160807
HGLibA_47595	STRN	19.25138523
HGLibB_44690	SLC25A43	18.90380305
HGLibA_22636	IFIT5	18.79207045
HGLibA_18669	GATA2	18.68229301
HGLibB_31847	NHLH1	18.57440983
HGLibA_30338	MTERF	18.46836274
HGLibB_37639	POP1	18.42399161
HGLibB_15980	FAM107B	18.36409612
HGLibA_43374	SEMA6D	18.2615568
HGLibA_44161	SIK1	18.25342673
HGLibA_33021	NUP133	18.16069386
HGLibA_46327	SPACA3	17.96380412
HGLibB_41066	RGSL1	17.86768582
HGLibB_39436	PTPRB	17.77306066
HGLibA_57790	hsa-mir-1343	17.67988739
HGLibB_13404	DMRT3	17.60751851

HGLibB_40124	RALY	17.58812641
HGLibA_48511	TBC1D24	17.49773967
HGLibB_48340	TAS2R4	17.40869057
HGLibA_63666	hsa-mir-6870	17.32094394
HGLibB_21346	HIST1H2AA	17.23446593
HGLibB_17032	FBXO25	17.14922394
HGLibB_06696	C9orf163	17.06518659
HGLibB_46672	SPN	16.98232364
HGLibB_15222	EPB41L2	16.90060594
HGLibA_46931	SRCAP	16.82000538
HGLibA_30967	N6AMT2	16.74049484
HGLibB_48062	T	16.66204815
HGLibA_21946	HOXB7	16.64450282
HGLibA_49804	TMED2	16.58464005
HGLibB_04118	BAI3	16.35840727
HGLibA_43852	SGPP2	16.28491751
HGLibA_39796	RAB11FIP2	16.2123522
HGLibB_10013	CLIC1	16.14069072
HGLibA_21728	HMGN5	16.06991309
HGLibA_38704	PROX2	16
HGLibB_44604	SLC25A17	15.93093272
HGLibB_08484	CD99_Y	15.8626931
HGLibB_07993	CCL23	15.79526358
HGLibA_47705	SUCNR1	15.72862711
HGLibB_34618	OSBPL7	15.5976677
HGLibA_49485	TIGIT	15.54548393
HGLibA_28897	MEP1B	15.53331318
HGLibB_41792	RPH3A	15.406779
HGLibB_13859	DPP8	15.34457046
HGLibB_34267	OR5K2	15.28304903
HGLibB_42639	SAMD7	15.22220129
HGLibA_44283	SKIL	15.10247503
HGLibB_32043	NLN	14.9852915
HGLibB_27574	LTN1	14.87055597
HGLibA_29795	MPZL1	14.81407799
HGLibA_23720	ITGA11	14.75817874
HGLibB_29624	MORN1	14.70284775
HGLibA_37292	PMAIP1	14.50070081
HGLibA_28650	MCRS1	14.48700599
HGLibB_06240	C3orf43	14.38224131
HGLibB_22173	HSD17B8	14.3306164
HGLibB_23324	INPP5F	14.27948497
HGLibA_39816	RAB15	14.1786695
HGLibA_36887	PLA2G2D	14.12896939
HGLibB_37251	PMEPA1	14.07973066

HGLibA_42744	SARDH	14.03094575
HGLibB_15782	EXT2	13.98260727
HGLibA_26113	LCE1E	13.88724098
HGLibB_25396	KRT16	13.74736504
HGLibA_00076	AASDH	13.70155957
HGLibA_51445	TRIM26	13.65615345
HGLibA_15707	EVX1	13.61114054
HGLibA_04677	BOK	13.56651484
HGLibA_42437	RUFY1	13.52227049
HGLibA_22263	HSPA12B	13.47840175
HGLibB_32631	NRG1	13.43490301
HGLibB_15052	EMP1	13.39176876
HGLibB_31561	NEDD4	13.34899364
HGLibA_27310	LRRC31	13.30657238
HGLibB_02248	ANXA7	13.09959745
HGLibA_62918	hsa-mir-6511b-2	13.05919487
HGLibA_51472	TRIM35	13.01911226
HGLibA_43860	SGSM1	12.97934516
HGLibA_20863	HAS1	12.93988922
HGLibB_20922	HCAR2	12.90074015
HGLibB_33821	OR2T27	12.86189376
HGLibA_48082	SYT5	12.82334593
HGLibA_60023	hsa-mir-4325	12.78509265
HGLibA_49612	TLE3	12.74712995
HGLibA_28519	MBOAT2	12.70945396
HGLibA_15172	ENPP3	12.67206087
HGLibB_23044	IL20RA	12.63494697
HGLibA_51441	TRIM25	12.59810858
HGLibB_36929	PLBD2	12.56154213
HGLibA_23621	IRS2	12.52524408
HGLibA_47139	SSH3	12.48921098
HGLibA_50664	TMSB15B	12.45343945
HGLibA_15437	ERCC3	12.41792614
HGLibA_56511	ZNF675	12.38266779
HGLibA_29186	MIA2	12.34766119
HGLibB_20799	HAND1	12.3129032
HGLibA_21128	HEMGN	12.2783907
HGLibB_40576	RBMY1J	12.17629613
HGLibB_40054	RAD54L2	12.14273579
HGLibA_26982	LOC649330	12.1094063
HGLibB_08202	CCT6B	12.07630486
HGLibB_15170	ENTHD1	12.04342875
HGLibB_11167	CRK	11.9783418
HGLibB_24343	KCNQ5	11.94612573
HGLibA_30164	MS4A7	11.91412452

HGLibB_20934	HCFC1R1	11.88233565
HGLibA_32309	NOP2	11.85075666
HGLibA_27000	LOC729059	11.81938514
HGLibB_11138	CRIP1	11.75725496
HGLibA_05345	C14orf178	11.72649166
HGLibB_49167	TGFBR2	11.69592652
HGLibB_32728	NSMCE4A	11.6655573
HGLibB_34348	OR6C6	11.63538181
HGLibA_61245	hsa-mir-4784	11.6053979
HGLibA_41825	RPE65	11.57560342
HGLibB_15524	ERVV-1	11.5459963
HGLibA_25265	KLK6	11.51657448
HGLibA_00344	ACAA2	11.48733592
HGLibA_61383	hsa-mir-496	11.45827865
HGLibB_35608	PCOLCE2	11.42940068
HGLibB_12912	DENND1A	11.4007001
HGLibA_05384	C15orf32	11.34382351
HGLibA_15525	ERP27	11.31564378
HGLibB_16256	FAM173B	11.287634
HGLibB_44303	SLC12A1	11.25979238
HGLibA_18591	GALR2	11.23211716
HGLibA_27237	LRPAP1	11.2046066
HGLibA_07241	CARD9	11.177259
HGLibB_23749	ITGB3	11.12304596
HGLibA_44447	SLC16A6	11.04290729
HGLibB_13195	DIABLO	11.01650294
HGLibB_18504	GALNT10	10.99025028
HGLibA_13371	DLX1	10.96414777
HGLibA_60796	hsa-mir-4670	10.93819394
HGLibA_26130	LCE3A	10.8867264
HGLibA_47585	STRC	10.86120981
HGLibA_37289	PM20D2	10.81060393
HGLibA_06843	CABLES1	10.78551187
HGLibA_46264	SOX4	10.76055858
HGLibB_04624	BMPER	10.73574273
HGLibB_50924	TOP1	10.71106301
HGLibB_34946	PAFAH1B2	10.66210675
HGLibB_23219	IMPACT	10.63782768
HGLibB_01413	AKAP13	10.56577176
HGLibB_08680	CDH6	10.54200951
HGLibB_29454	MMP15	10.51837347
HGLibA_32910	NUCB1	10.49486249
HGLibB_33009	NUP43	10.4714754
HGLibB_24882	KIF26A	10.44821108
HGLibA_08202	CCT4	10.42506842

HGLibA_09759	CKAP4	10.40204629
HGLibB_28404	MAVS	10.37914362
HGLibA_61890	hsa-mir-548aj-2	10.35635932
HGLibA_07733	CCDC23	10.31114162
HGLibA_59895	hsa-mir-4296	10.26638486
HGLibB_15064	EMR2	10.24417679
HGLibB_38081	PPP1R9B	10.22208092
HGLibB_07905	CCDC88C	10.15645679
HGLibA_19706	GP2	10.13480003
HGLibA_28364	MARK1	10.07047055
HGLibB_19682	GP6	10.04923795
HGLibB_35400	PCDHA5	10.02810913
HGLibA_22343	HSPH1	9.986159347
HGLibA_60708	hsa-mir-4650-2	9.965336655
HGLibB_42987	SCPEP1	9.944614296
HGLibA_61615	hsa-mir-516b-1	9.923991434
HGLibB_42404	RUNX1T1	9.903467242
HGLibB_18567	GALR3	9.862711609
HGLibA_28775	MED21	9.842478562
HGLibA_45506	SLIT1	9.822340973
HGLibB_14070	DTX4	9.802298065
HGLibA_43806	SGCE	9.762493216
HGLibB_37420	POFUT1	9.723057962
HGLibA_12461	DCAF8L1	9.703477079
HGLibB_33952	OR4F21	9.683986388
HGLibB_23367	INSR	9.664585172
HGLibB_30917	N4BP2L2	9.645272718
HGLibA_34694	OSM	9.626048328
HGLibA_50734	TNFRSF10A	9.606911305
HGLibB_34260	OR5I1	9.587860965
HGLibA_62746	hsa-mir-627	9.568896629
HGLibB_19534	GNPAT	9.550017627
HGLibB_01488	AKR1C4	9.531223295
HGLibB_05917	C1QC	9.493886027
HGLibA_07588	CCDC13	9.475341802
HGLibA_43966	SH3PXD2B	9.456879667
HGLibB_28450	MBD5	9.438498997
HGLibA_12042	CYLC1	9.420199171
HGLibA_42845	SCAMP4	9.401979574
HGLibA_64073	hsa-mir-8059	9.365778651
HGLibA_58554	hsa-mir-3125	9.347796131
HGLibA_35363	PCBD2	9.294313304
HGLibA_57430	hsa-mir-1256	9.276638691
HGLibA_15682	EVC2	9.259039633
HGLibA_38084	PPP1R27	9.241515574

HGLibA_51683	TRMT2A	9.224065962
HGLibB_10411	CNTNAP2	9.206690253
HGLibA_23402	INSRR	9.189387908
HGLibB_37972	PPP1R12A	9.15500118
HGLibB_14599	EGF	9.137915746
HGLibB_42116	RPS6KA5	9.120901575
HGLibB_22794	IGFBP4	9.08708498
HGLibA_23210	ILDR2	9.070281547
HGLibB_39385	PTPN11	9.053547361
HGLibB_38166	PPP5D1	9.020284771
HGLibB_01561	ALDH3A1	9.0037554
HGLibA_34659	OSBPL5	8.95456928
HGLibB_07710	CCDC178	8.938306349
HGLibB_01330	AIF1	8.922108873
HGLibB_17107	FBXO8	8.905976399
HGLibA_51552	TRIM58	8.889908478
HGLibB_03965	B3GALT6	8.873904666
HGLibB_10073	CLN6	8.842087619
HGLibB_03927	AZGP1	8.810521791
HGLibB_24122	KCNB1	8.79483202
HGLibA_29692	MOV10	8.779203785
HGLibA_30852	MYO5B	8.763636672
HGLibA_08500	CDADC1	8.74813027
HGLibB_09676	CIB2	8.701971285
HGLibA_18747	GBP7	8.686703701
HGLibB_19912	GPR153	8.671494835
HGLibA_08272	CD1D	8.656344299
HGLibA_37832	PPAPDC2	8.641251709
HGLibA_13856	DPM1	8.626216684
HGLibB_34727	OTUD6B	8.611238849
HGLibA_39645	PXDC1	8.596317831
HGLibA_48674	TBX5	8.581453259
HGLibB_04242	BCAR1	8.551891995
HGLibB_29968	MRPL50	8.53719458
HGLibA_43022	SCNN1G	8.522552169
HGLibB_02529	APOL2	8.507964407
HGLibB_46110	SOHLH2	8.493430945
HGLibA_01468	AKR1A1	8.464525542
HGLibB_24763	KIAA1644	8.450152916
HGLibB_28937	METTL21B	8.421566134
HGLibB_49901	TMEM140	8.407351313
HGLibA_07363	CATSPER4	8.393188433
HGLibA_34851	P2RX3	8.379077171
HGLibA_39511	PTPRJ	8.365017203
HGLibA_43532	SERPINA9	8.35100821

HGLibB_25033	KLF3	8.337049877
HGLibB_36845	PLA2G2E	8.32314189
HGLibB_22106	HS3ST5	8.309283939
HGLibA_24867	KIF16B	8.295475714
HGLibA_05091	C10orf71	8.281716913
HGLibB_20322	GRM4	8.254346368
HGLibB_16828	FANCG	8.240734028
HGLibB_08532	CDC27	8.227169917
HGLibB_12545	DCTN6	8.200185212
HGLibA_41873	RPL14	8.186764042
HGLibA_60488	hsa-mir-4511	8.173389947
HGLibA_41142	RHBDL3	8.160062645
HGLibB_09829	CLCN7	8.146781855
HGLibB_36960	PLCH1	8.133547302
HGLibA_45220	SLC4A4	8.107215805
HGLibB_39099	PSMD1	8.094118318
HGLibB_50875	TOB2	8.081065982
HGLibB_01151	AGAP6	8.06805853
HGLibB_17145	FCER1A	8.055095699
HGLibA_57313	hsa-mir-1229	8.042177227
HGLibA_40791	REEP6	8.003685391
HGLibA_37855	PPBP	7.990941789
HGLibA_43190	SEC14L1	7.965583595
HGLibB_23776	ITIH3	7.952968507
HGLibA_23019	IL17REL	7.940395766
HGLibA_24199	KCNG1	7.90292921
HGLibA_62778	hsa-mir-635	7.890523452
HGLibB_28282	MAPT	7.878158848
HGLibA_46299	SP3	7.865835166
HGLibA_21143	HEPH	7.841309647
HGLibB_42244	RSAD1	7.792739644
HGLibA_48715	TCEAL5	7.780696057
HGLibB_46369	SPATA12	7.768691598
HGLibA_23975	JOSD2	7.732910827
HGLibB_40826	RETNLB	7.72106073
HGLibB_28814	MEGF8	7.709248694
HGLibA_13859	DPM2	7.697474513
HGLibA_23809	ITIH2	7.685737979
HGLibA_23358	INPP5J	7.674038889
HGLibB_00555	ACSL3	7.662377038
HGLibB_18975	GGN	7.639164252
HGLibA_18397	GABRP	7.604619384
HGLibB_12229	CYTH3	7.593176794
HGLibA_36482	PI15	7.570399007
HGLibB_45696	SMIM22	7.547763145

HGLibA_09842	CLCNKA	7.525267709
HGLibA_21694	HMGB2	7.514072188
HGLibB_29546	MOB1B	7.502911222
HGLibB_29593	MOGS	7.491784629
HGLibA_21485	HIST1H4A	7.469633848
HGLibA_26795	LOC100130705	7.458609303
HGLibA_62934	hsa-mir-6515	7.447618421
HGLibB_25113	KLHL2	7.436661027
HGLibA_22867	IGLL1	7.425736949
HGLibB_51977	TTBK2	7.414846014
HGLibB_21284	HIGD1C	7.393162893
HGLibA_23183	IL6R	7.38237037
HGLibB_24351	KCNS1	7.360882564
HGLibB_44030	SHQ1	7.339523314
HGLibB_50537	TMOD2	7.32889149
HGLibB_24700	KIAA1279	7.318291318
HGLibA_40134	RAG2	7.30772264
HGLibA_38610	PRM2	7.297185296
HGLibA_19814	GPKOW	7.286679128
HGLibA_02706	ARHGAP11B	7.276203982
HGLibA_61932	hsa-mir-548av	7.265759701
HGLibB_22497	ID2	7.255346132
HGLibA_61253	hsa-mir-4786	7.244963122
HGLibA_19278	GLIS3	7.234610518
HGLibA_57949	hsa-mir-16-2	7.213995929
HGLibA_10456	COA6	7.203733646
HGLibB_04813	BRINP2	7.193501173
HGLibB_15362	EPS8L3	7.183298363
HGLibB_48883	TECPR1	7.173125072
HGLibB_07481	CCAR1	7.162981154
HGLibA_09254	CFD	7.152866467
HGLibA_47384	STAT2	7.132724214
HGLibA_31306	NCK1	7.122696366
HGLibB_36056	PEX16	7.112697185
HGLibA_47966	SYNCRIP	7.102726531
HGLibA_19268	GLIPR2	7.092784267
HGLibB_36979	PLCXD2	7.082870257
HGLibA_47105	SS18L1	7.072984365
HGLibB_15405	ERC2	7.063126456
HGLibB_00789	ADAMTS1	7.053296396
HGLibB_27699	LYPLA2	7.043494052
HGLibB_27172	LRP2	7.023971985
HGLibA_09445	CHIC1	7.014252002
HGLibA_42303	RSBN1	7.004559211
HGLibA_48155	TAC4	6.994893486

HGLibB_11649	CTNND2	6.985254698
HGLibB_24323	KCNN2	6.975642721
HGLibA_26469	LHX9	6.956498696
HGLibA_44151	SIGLEC8	6.927980619
HGLibA_00430	ACER2	6.918526891
HGLibB_18678	GATSL2	6.899697157
HGLibA_48883	TDGF1	6.880970253
HGLibB_00727	ADAM10	6.871645067
HGLibB_10081	CLNS1A	6.862345235
HGLibA_46798	SPRN	6.853070641
HGLibB_43019	SCUBE2	6.84382117
HGLibA_58032	hsa-mir-1909	6.816222347
HGLibB_40117	RALGPS1	6.807072225
HGLibA_59542	hsa-mir-3916	6.797946661
HGLibA_21855	HNRPDL	6.788845541
HGLibA_58810	hsa-mir-3180-3	6.779768757
HGLibB_18468	GAL	6.770716198
HGLibB_30396	MTMR8	6.761687756
HGLibA_40224	RAP1B	6.752683323
HGLibB_46013	SNX24	6.74370279
HGLibB_33826	OR2T3	6.734746051
HGLibB_37214	PLXNA3	6.725813
HGLibB_39486	PTPRS	6.716903532
HGLibB_39587	PWP2	6.708017542
HGLibA_10777	CORO1B	6.699154925
HGLibA_62333	hsa-mir-570	6.690315578
HGLibA_26916	LOC284385	6.681499399
HGLibB_22654	IFNA6	6.672706285
HGLibA_47730	SULT1A1	6.663936135
HGLibA_61130	hsa-mir-4758	6.655188847
HGLibA_03845	ATXN3L	6.646464322
HGLibA_51357	TREM2	6.637762461
HGLibB_35175	PARP12	6.629083163
HGLibB_47289	STAP2	6.611791867
HGLibB_37500	POLR1A	6.603179673
HGLibB_25353	KRBA2	6.586021713
HGLibA_28375	MARS	6.577475755
HGLibB_19069	GIMD1	6.568951686
HGLibB_18841	GDF3	6.56044941
HGLibB_21195	HEYL	6.543509867
HGLibA_39203	PSMF1	6.535072414
HGLibA_11109	CREBBP	6.493204675
HGLibA_35325	PAXIP1	6.484894401
HGLibA_61080	hsa-mir-4746	6.47660501
HGLibB_18621	GAS2L2	6.468336415

HGLibB_43306	SEMA5A	6.460088527
HGLibA_34026	OR4K15	6.45186126
HGLibB_41107	RHEBL1	6.443654527
HGLibB_14603	EGFL6	6.435468242
HGLibB_19014	GHR	6.427302321
HGLibB_13458	DNAH12	6.419156677
HGLibA_10494	COIL	6.411031226
HGLibB_01368	AJAP1	6.402925885
HGLibA_59691	hsa-mir-421	6.39484057
HGLibA_21377	HIST1H2AB	6.386775198
HGLibB_40277	RASGEF1A	6.370703954
HGLibA_32448	NPHP3	6.362697919
HGLibA_45429	SLC9A9	6.3547115
HGLibB_50547	TMPO	6.346744616
HGLibA_35441	PCDHA3	6.338797188
HGLibB_05859	C1orf51	6.330869137
HGLibA_47743	SULT1B1	6.322960382
HGLibA_31122	NAP1L5	6.307200449
HGLibA_44365	SLC12A4	6.299349115
HGLibB_49807	TMEM109	6.291516766
HGLibA_33557	OR10X1	6.283703325
HGLibA_57522	hsa-mir-1271	6.268132863
HGLibA_42701	SAMD9L	6.26037569
HGLibB_38343	PRDM5	6.252637122
HGLibA_05639	C19orf24	6.244917085
HGLibA_12109	CYP2A7	6.237215505
HGLibA_12684	DDX26B	6.229532306
HGLibB_10468	COG4	6.221867417
HGLibB_14880	ELF5	6.214220764
HGLibB_19768	GPER1	6.206592275
HGLibB_05473	C16orf72	6.198981877
HGLibB_50503	TMEM95	6.191389499
HGLibB_38205	PQLC2	6.176258517
HGLibB_23884	JAK3	6.168719772
HGLibB_39567	PVR	6.161198763
HGLibB_49694	TMC3	6.153695422
HGLibB_04863	BSG	6.146209678
HGLibA_49628	TLL1	6.131290709
HGLibB_21971	HOXD4	6.116441308
HGLibA_44455	SLC16A9	6.109042527
HGLibA_63139	hsa-mir-6746	6.101660935
HGLibB_49653	TM6SF2	6.094296467
HGLibA_58574	hsa-mir-3130-2	6.086949055
HGLibA_08232	CD109	6.079618634
HGLibA_10665	COMMD4	6.065008503

HGLibA_13144	DHRS7	6.057728662
HGLibA_22416	HTRA4	6.050465552
HGLibA_44227	SIRT5	6.043219109
HGLibA_57982	hsa-mir-182	6.035989269
HGLibB_07005	CALD1	6.028775967
HGLibB_14063	DTX3	6.021579142
HGLibB_35998	PEMT	6.01439873
HGLibA_23262	IMPG1	6.007234669
HGLibB_16163	FAM156A	5.978740698
HGLibB_09018	CELSR2	5.971657468
HGLibA_08407	CD53	5.964590221
HGLibB_07698	CCDC174	5.957538897
HGLibA_00869	ADAR	5.950503436
HGLibA_41021	RGP1	5.936479869
HGLibA_08877	CDRT15L2	5.929491644
HGLibB_09617	CHST14	5.922519046
HGLibA_21881	HOOK3	5.908620501
HGLibB_32688	NRXN1	5.894783773
HGLibA_14002	DSCAML1	5.887888447
HGLibA_41558	RNF166	5.881008403
HGLibB_00664	ACTR3C	5.874143587
HGLibB_23118	IL36B	5.867293941
HGLibA_33140	NXN	5.846835469
HGLibA_23592	IRF5	5.840045951
HGLibB_49241	THAP4	5.833271326
HGLibB_50077	TMEM19	5.826511542
HGLibB_24832	KIF15	5.819766544
HGLibB_14417	EDARADD	5.813036279
HGLibA_26908	LOC283403	5.806320692
HGLibA_07395	CBL	5.799619731
HGLibA_07682	CCDC169	5.792933342
HGLibA_06341	C4orf48	5.786261473
HGLibA_09066	CENPK	5.779604072
HGLibA_40524	RBM27	5.772961087
HGLibA_64282	hsa-mir-922	5.766332465
HGLibB_47452	STK4	5.753118105
HGLibB_03228	ASB18	5.746532265
HGLibA_25042	KLF10	5.739960584
HGLibB_38935	PRTN3	5.733403011
HGLibB_22573	IFI30	5.726859496
HGLibB_16261	FAM174A	5.720329989
HGLibB_15564	ESRP1	5.71381444
HGLibB_49665	TM9SF2	5.707312799
HGLibA_46843	SPRY3_Y	5.700825018
HGLibB_23381	INTS12	5.694351047

HGLibB_07478	CC2D2B	5.687890837
HGLibB_08888	CDX1	5.68144434
HGLibB_44311	SLC12A3	5.668592292
HGLibA_30643	MYBL1	5.662186645
HGLibA_28423	MATK	5.649415867
HGLibB_28061	MANSC4	5.643050641
HGLibB_38099	PPP2R2B	5.636698795
HGLibA_22427	HUS1B	5.630360283
HGLibB_13492	DNAJA1	5.624035057
HGLibA_39820	RAB17	5.617723072
HGLibB_32039	NLK	5.611424281
HGLibA_10696	COPB1	5.605138639
HGLibB_53993	WDR27	5.598866101
HGLibA_60835	hsa-mir-4681	5.592606621
HGLibB_34506	OR8K5	5.586360154
HGLibA_39010	PSCA	5.580126655
HGLibB_41290	RLBP1	5.57390608
HGLibB_22064	HRH1	5.561503524
HGLibB_26140	LCN2	5.555321455
HGLibA_51913	TSPAN17	5.549152133
HGLibB_11832	CXCL10	5.542995515
HGLibB_18751	GCG	5.536851558
HGLibA_35298	PAX2	5.530720218
HGLibB_22463	ICAM1	5.524601453
HGLibB_37900	PPIL4	5.518495219
HGLibA_42819	SC5D	5.512401475
HGLibA_25489	KRT35	5.506320179
HGLibA_59674	hsa-mir-409	5.500251287
HGLibA_29548	MMRN2	5.494194759
HGLibA_41262	RIMBP3	5.488150552
HGLibA_15159	ENOX1	5.482118625
HGLibA_17769	FNIP2	5.476098937
HGLibA_23958	JMJD6	5.464096114
HGLibA_02860	ARHGEF39	5.458112897
HGLibA_34377	OR6B2	5.452141756
HGLibA_32159	NME3	5.44618265
HGLibB_07108	CAMSAP3	5.440235539
HGLibB_26622	LMAN1L	5.434300384
HGLibA_61741	hsa-mir-520c	5.428377145
HGLibB_46598	SPIB	5.422465781
HGLibA_63985	hsa-mir-7849	5.416566255
HGLibB_16016	FAM115C	5.410678526
HGLibA_41005	RGL2	5.404802556
HGLibB_11572	CTBP1	5.398938305
HGLibA_21352	HIRA	5.38724481

HGLibB_21172	HEXA	5.381415488
HGLibB_21183	HEXIM1	5.375597733
HGLibB_14847	ELAC1	5.369791507
HGLibA_31871	NGF	5.363996771
HGLibB_05816	C1orf198	5.352441623
HGLibB_01646	ALKBH2	5.346681135
HGLibA_57472	hsa-mir-1262	5.340931989
HGLibA_24477	KDELC2	5.335194149
HGLibA_00847	ADAMTSL1	5.329467576
HGLibA_09856	CLDN12	5.323752235
HGLibA_30554	MUC6	5.31804809
HGLibA_38535	PRKCB	5.312355104
HGLibB_29730	MPPED1	5.306673241
HGLibB_12946	DENND6A	5.301002465
HGLibA_61360	hsa-mir-490	5.289694034
HGLibA_63022	hsa-mir-6715b	5.278429526
HGLibB_01937	ANK3	5.261614506
HGLibB_34665	OSTF1	5.256031159
HGLibA_50879	TNNI3K	5.250458583
HGLibB_05602	C18orf21	5.244896746
HGLibA_34824	OXLD1	5.239345612
HGLibA_61646	hsa-mir-5189	5.22827532
HGLibB_15399	ERBB4	5.217247438
HGLibB_30179	MSLN	5.211749317
HGLibB_38803	PRR7	5.206261699
HGLibB_45854	SNCAIP	5.20078455
HGLibB_25352	KRBA1	5.195317837
HGLibB_45788	SMUG1	5.184415591
HGLibB_46639	SPINK5	5.178979992
HGLibB_20230	GRHPR	5.173554699
HGLibB_47890	SYK	5.168139681
HGLibA_40217	RANGRF	5.162734906
HGLibA_50437	TMEM53	5.15734034
HGLibB_43790	SGOL2	5.151955954
HGLibB_09892	CLDN5	5.146581714
HGLibB_21667	HMGB3	5.130519564
HGLibA_18285	FZD8	5.1251856
HGLibB_31695	NEUROD2	5.119861627
HGLibA_51248	TRAF3IP1	5.114547615
HGLibB_04223	BBS7	5.109243532
HGLibA_03364	ASPSCR1	5.103949349
HGLibB_09683	CIB4	5.098665035
HGLibA_58245	hsa-mir-212	5.093390559
HGLibB_47763	SUPT5H	5.088125892
HGLibA_46852	SPRYD4	5.082871004

HGLibB_19782	GPIHBP1	5.077625865
HGLibB_29372	MLLT11	5.061948644
HGLibA_40459	RBFOX2	5.056742205
HGLibA_43866	SGSM3	5.046358102
HGLibB_07382	CBFA2T3	5.036012173
HGLibA_19376	GLYATL2	5.030853453
HGLibB_46122	SORBS2	5.025704189
HGLibA_48693	TCAP	5.020564355
HGLibB_50715	TNFRSF21	5.015433922
HGLibA_29168	MGP	5.010312861
HGLibA_53226	USP17L15	5.005201144
HGLibB_28031	MAN2A2	5.000098744
HGLibA_03436	ATF3	4.995005633
HGLibA_04090	BAG6	4.989921783
HGLibA_29865	MRO	4.984847165
HGLibA_59178	hsa-mir-3658	4.979781754
HGLibB_29359	MLL3	4.974725521
HGLibA_38111	PPP1R3E	4.959611621
HGLibB_17092	FBXO47	4.95459183
HGLibB_30490	MUC17	4.949581082
HGLibA_33288	ODF3B	4.93958661
HGLibA_60103	hsa-mir-4429	4.934602832
HGLibB_43403	9-Sep	4.929627992
HGLibB_21508	HIST2H2BF	4.919705017
HGLibA_23578	IRF2BP1	4.909817478
HGLibA_12879	DEFB125	4.895052156
HGLibB_44227	SKIDA1	4.890147877
HGLibA_29673	MORN3	4.885252302
HGLibB_11893	CXorf22	4.880365407
HGLibA_33745	OR2AE1	4.875487166
HGLibA_39242	PSTPIP2	4.865756547
HGLibB_47623	STXBP6	4.860904119
HGLibB_48238	TANGO2	4.856060246
HGLibA_61247	hsa-mir-4785	4.851224903
HGLibA_49707	TM4SF5	4.846398065
HGLibB_17378	FGF23	4.841579708
HGLibB_23102	IL31RA	4.836769807
HGLibB_36519	PIGM	4.831968339
HGLibB_39291	PTGFR	4.817614286
HGLibB_08578	CDC42SE2	4.812846307
HGLibA_50466	TMEM60	4.808086639
HGLibA_20967	HCK	4.798592147
HGLibB_09543	CHRM3	4.793857275
HGLibB_30428	MTRF1L	4.789130622
HGLibB_33833	OR2T34	4.784412163

HGLibA_12712	DDX41	4.779701876
HGLibA_55512	ZNF160	4.774999738
HGLibB_03022	ARMC5	4.770305726
HGLibB_26218	LDOC1	4.765619816
HGLibA_37135	PLEKHJ1	4.760941986
HGLibA_24350	KCNMB4	4.751610475
HGLibA_39065	PSKH1	4.746956749
HGLibB_49031	TEX29	4.742311012
HGLibA_43129	SDF4	4.737673242
HGLibB_35630	PCSK4	4.733043417
HGLibA_61918	hsa-mir-548ar	4.728421515
HGLibA_64199	hsa-mir-8089	4.723807513
HGLibB_40617	RCAN1	4.714603122
HGLibB_31670	NEU2	4.710012689
HGLibA_13319	DLEC1	4.700855241
HGLibB_11220	CRTC2	4.696288182
HGLibA_40370	RASL12	4.691728872
HGLibA_26539	LIMK1	4.687177287
HGLibA_58236	hsa-mir-2116	4.682633408
HGLibB_24490	KDM5B	4.678097214
HGLibB_07525	CCDC109B	4.673568682
HGLibB_34714	OTUD1	4.669047791
HGLibB_42823	SCARF1	4.664534522
HGLibB_44320	SLC12A6	4.660028852
HGLibA_27071	LPCAT1	4.655530761
HGLibA_37062	PLEKHA1	4.651040229
HGLibB_07471	CC2D1B	4.637613774
HGLibB_04188	BAZ2A	4.633153268
HGLibA_32058	NKX6-2	4.619816403
HGLibB_10719	COPS7B	4.615385598
HGLibA_45618	SMARCAL1	4.610962168
HGLibB_12920	DENND2A	4.606546093
HGLibA_49124	TFAP2B	4.602137352
HGLibB_20252	GRIK1	4.597735927
HGLibB_27665	LYN	4.593341798
HGLibA_38344	PRB3	4.588954944
HGLibA_28896	MEP1A	4.584575346
HGLibB_09570	CHRNA6	4.580202986
HGLibA_23542	IQSEC1	4.571479896
HGLibA_48647	TBX15	4.56278552
HGLibA_61771	hsa-mir-521-2	4.558449052
HGLibB_41363	RNASE7	4.554119706
HGLibB_07835	CCDC68	4.549797461
HGLibB_11026	CPXM2	4.545482299
HGLibA_06889	CACNA1F	4.53687315

HGLibA_38270	PRAF2	4.532579124
HGLibA_07943	CCER1	4.528292107
HGLibA_06163	C2orf68	4.52401208
HGLibA_22078	HRASLS2	4.519739023
HGLibA_12743	DDX54	4.515472919
HGLibA_08391	CD44	4.511213749
HGLibA_20721	GZMB	4.506961495
HGLibB_49711	TMCC1	4.502716139
HGLibA_33250	OCIAD2	4.494246047
HGLibB_23854	IYD	4.490021275
HGLibB_17175	FCGR3B	4.485803328
HGLibB_44411	SLC17A3	4.481592189
HGLibA_24088	KAZALD1	4.477387839
HGLibA_22581	IER5	4.473190262
HGLibA_49700	TM4SF20	4.468999438
HGLibB_12365	DBF4B	4.464815352
HGLibB_38809	PRRC1	4.460637984
HGLibA_24073	KATNA1	4.456467318
HGLibB_07119	CAND2	4.452303335
HGLibB_40654	RCN2	4.43985132
HGLibB_31144	NAT8B	4.435713901
HGLibA_59365	hsa-mir-3713	4.431583079
HGLibA_09317	CGGBP1	4.427458839
HGLibA_31567	NDUFS8	4.419230032
HGLibA_03734	ATP6V1B1	4.415125433
HGLibA_47738	SULT1A3	4.411027346
HGLibB_10576	COL5A1	4.406935756
HGLibA_49381	THOP1	4.402850645
HGLibA_39942	RAB41	4.398771998
HGLibA_47687	STYX	4.394699797
HGLibA_46243	SOX15	4.390634026
HGLibA_46701	SPINK6	4.386574669
HGLibA_29042	MFAP2	4.378475131
HGLibA_62649	hsa-mir-6125	4.374434916
HGLibA_04833	BRMS1L	4.366373517
HGLibA_40965	RFX2	4.358337382
HGLibA_59253	hsa-mir-3675	4.354328749
HGLibA_24172	KCNC4	4.350326384
HGLibA_37285	PM20D1	4.346330272
HGLibB_00819	ADAMTS2	4.342340396
HGLibB_21506	HIST2H2BE	4.338356741
HGLibA_48309	TAOK2	4.326442945
HGLibB_33449	OR10G4	4.322484017
HGLibA_29577	MNT	4.318531233
HGLibA_25803	KRTAP5-6	4.31064403

HGLibA_37182	PLIN5	4.302781216
HGLibB_23598	IRX2	4.294942667
HGLibB_14480	EEF1E1	4.291032455
HGLibA_38825	PRR23B	4.287128263
HGLibA_24748	KIAA1407	4.283230078
HGLibB_24858	KIF20A	4.279337884
HGLibA_01673	ALMS1	4.275451667
HGLibA_47469	STK19	4.267697102
HGLibA_20283	GRIK2	4.263828726
HGLibA_24336	KCNK9	4.259966266
HGLibB_18527	GALNT18	4.25610971
HGLibA_52005	TSSC4	4.252259041
HGLibA_58291	hsa-mir-221	4.248414247
HGLibA_09144	CEP55	4.244575312
HGLibB_22741	IFT80	4.240742221
HGLibB_40501	RBM42	4.236914961
HGLibB_40868	RFFL	4.233093518
HGLibB_30264	MT3	4.229277876
HGLibA_41212	RHOV	4.225468022
HGLibB_03797	ATPAF1	4.221663941
HGLibA_23379	INSIG2	4.21786562
HGLibA_18376	GABRB2	4.214073045
HGLibB_21772	HNRNPA3	4.2102862
HGLibB_30664	MYH10	4.206505073
HGLibB_50426	TMEM65	4.20272965
HGLibB_40832	REV1	4.198959916
HGLibA_06699	C9orf16	4.195195858
HGLibA_05277	C12orf61	4.191437462
HGLibB_29603	MON2	4.187684714
HGLibB_18212	FXYD5	4.183937601
HGLibA_45873	SNAI3	4.176460224
HGLibB_07467	CBY3	4.169005222
HGLibA_28127	MAP1LC3B2	4.165286078
HGLibA_60949	hsa-mir-4712	4.161572488
HGLibB_26839	LOC100862671	4.154161914
HGLibA_59921	hsa-mir-4302	4.146773395
HGLibA_20492	GSTP1	4.143087372
HGLibA_31555	NDUFS4	4.139406824
HGLibA_26044	LARP7	4.135731737
HGLibA_62165	hsa-mir-5581	4.132062097
HGLibA_22368	HTR1F	4.128397892
HGLibA_40644	RBP7	4.124739109
HGLibA_05794	C1orf173	4.121085736
HGLibA_60736	hsa-mir-4657	4.117437758
HGLibB_02209	ANPEP	4.113795164

HGLibA_34724	OTOA	4.11015794
HGLibB_49037	TEX33	4.106526074
HGLibA_60718	hsa-mir-4653	4.099278365
HGLibA_25960	LAMB2	4.088446668
HGLibB_41908	RPL36AL	4.084846684
HGLibA_35505	PCDHB6	4.08125197
HGLibB_44556	SLC23A2	4.074078301
HGLibA_62758	hsa-mir-630	4.070499321
HGLibB_38094	PPP2R1B	4.066925563
HGLibB_18091	FTH1	4.056235487
HGLibA_57225	hsa-mir-1183	4.052682488
HGLibA_60527	hsa-mir-451b	4.049134649
HGLibA_48456	TAZ	4.045591958
HGLibB_46062	SOAT2	4.042054402
HGLibA_17912	FOXN2	4.03852197
HGLibA_42012	RPLP1	4.034994649
HGLibA_27937	MAGEB17	4.031472429
HGLibB_30951	NAA38	4.027955296
HGLibB_04522	BIRC6	4.026970699
HGLibB_06701	C9orf170	4.02444324
HGLibB_38732	PRR14L	4.020936249
HGLibB_50128	TMEM204	4.01743431
HGLibA_39789	RAB11B	4.013937412
HGLibA_33355	OLFML1	4.006958694
HGLibA_10381	CNRIP1	4.003476849
HGLibA_51788	TRPV3	4
HGLibA_19372	GLYATL1	4
HGLibA_27999	MAGI2	3.996528134
HGLibB_46970	SRPR	3.99306124
HGLibA_25511	KRT5	3.989599306
HGLibA_24370	KCNQ2	3.986142321
HGLibA_40936	RFPL3	3.982690273
HGLibA_01443	AKAP7	3.979243152
HGLibA_34406	OR6C74	3.975800946
HGLibA_26146	LCE4A	3.972363644
HGLibB_16867	FAS	3.968931235
HGLibA_23252	IMPAD1	3.965503707
HGLibA_36859	PKP3	3.962081049
HGLibA_35064	PALLD	3.958663251
HGLibA_41771	ROR2	3.951842188
HGLibA_27971	MAGED2	3.948438901
HGLibA_07644	CCDC150	3.945040429
HGLibB_47915	SYNDIG1L	3.941646762
HGLibA_58333	hsa-mir-23b	3.938257888
HGLibB_16796	FAM9A	3.934873797

HGLibA_28397	MASP2	3.921385043
HGLibA_37253	PLXDC1	3.918024703
HGLibA_59656	hsa-mir-3974	3.914669082
HGLibB_07970	CCL16	3.911318168
HGLibA_44620	SLC24A3	3.907971952
HGLibB_03455	ATF7IP2	3.904630422
HGLibA_50404	TMEM44	3.901293568
HGLibB_33519	OR11A1	3.89796138
HGLibA_43694	SF3A2	3.894633847
HGLibA_46156	SOGA2	3.891310958
HGLibB_12316	DAPK3	3.884679074
HGLibB_16242	FAM171A1	3.881370058
HGLibA_32730	NRTN	3.878065645
HGLibB_40985	RGPD5	3.871470589
HGLibB_26235	LEF1	3.868179925
HGLibA_23353	INPP5E	3.864893824
HGLibB_19675	GP1BB	3.861612276
HGLibB_30687	MYH6	3.85833527
HGLibA_34472	OR7D2	3.855062796
HGLibB_47689	SULT1C2	3.851794845
HGLibA_40349	RASGRP3	3.848531407
HGLibB_05337	C14orf177	3.845272471
HGLibA_32978	NUDT4	3.842018027
HGLibA_63190	hsa-mir-6758	3.835522578
HGLibA_36439	PHLPP2	3.829044981
HGLibA_38262	PRAC1	3.822585158
HGLibB_19345	GLYAT	3.819361887
HGLibA_30258	MST1R	3.812928579
HGLibA_64334	hsa-mir-940	3.809718522
HGLibA_50176	TMEM200C	3.80651285
HGLibB_08957	CEBPE	3.803311554
HGLibB_46468	SPATA6L	3.800114625
HGLibB_51043	TPCN1	3.796922053
HGLibA_10100	CLPP	3.793733828
HGLibB_34896	PABPN1	3.79054994
HGLibB_00011	A2ML1	3.787370382
HGLibB_40674	RD3	3.784195142
HGLibA_03548	ATP10B	3.781024212
HGLibA_12067	CYP1A1	3.777857583
HGLibB_35701	PDCL	3.774695244
HGLibB_17010	FBXO16	3.771537188
HGLibA_37422	PNPLA7	3.768383404
HGLibA_36956	PLAGL2	3.765233884
HGLibB_45906	SNRPA	3.762088617
HGLibB_07944	CCK	3.758947596

HGLibA_12903	DEFB133	3.755810811
HGLibA_24793	KIAA1614	3.752678252
HGLibA_51926	TSPAN3	3.749549912
HGLibB_36343	PHF8	3.74642578
HGLibA_43556	SERPINB5	3.743305847
HGLibA_12537	DCT	3.737078546
HGLibA_02246	ANXA6	3.727768867
HGLibB_49599	TLR6	3.724673945
HGLibB_14065	DTX3L	3.72158316
HGLibA_42710	SAMSN1	3.718496503
HGLibA_39182	PSMD7	3.715413967
HGLibB_30304	MTERFD3	3.709261217
HGLibB_19032	GID8	3.703124842
HGLibA_01411	AKAP12	3.700062773
HGLibB_12785	DEFA3	3.698970004
HGLibB_26922	LOC643037	3.698970004
HGLibB_37675	POTEC	3.697004772
HGLibB_05968	C1RL	3.690900938
HGLibA_00738	ADAM15	3.681775479
HGLibA_06727	C9orf40	3.663632778
HGLibA_27456	LRRIQ1	3.660622924
HGLibB_22952	IL15RA	3.657617029
HGLibA_46110	SNX9	3.654615083
HGLibA_25303	KLRF1	3.651617078
HGLibA_12591	DDAH2	3.648623006
HGLibA_24347	KCNMB3	3.642646629
HGLibA_51891	TSPAN1	3.639664308
HGLibB_37160	PLOD2	3.636685887
HGLibA_00846	ADAMTS9	3.633711358
HGLibA_04444	BEX2	3.630740713
HGLibA_04411	BEND5	3.627773945
HGLibB_45458	SLIT3	3.624811045
HGLibA_44633	SLC25A1	3.621852005
HGLibB_48374	TAS2R9	3.618896817
HGLibA_38654	PROCA1	3.615945474
HGLibA_29498	MMP16	3.612997966
HGLibA_31804	NFIB	3.610054288
HGLibA_35964	PDXP	3.607114429
HGLibA_48117	SZT2	3.604178384
HGLibA_30443	MTMR9	3.598317699
HGLibA_52151	TTC7A	3.595393044
HGLibA_50262	TMEM232	3.589555071
HGLibA_44718	SLC25A35	3.586641737
HGLibB_28113	MAP2K6	3.583732161
HGLibB_05334	C14orf169	3.580826336

HGLibA_36649	PIK3R1	3.577924254
HGLibA_49620	TLE6	3.575025906
HGLibB_36046	PEX12	3.572131287
HGLibA_22388	HTR3D	3.570086302
HGLibA_44519	SLC1A5	3.569240387
HGLibB_45735	SMO	3.5663532
HGLibA_41182	RHOC	3.563469718
HGLibA_31869	NGEF	3.560589933
HGLibA_59510	hsa-mir-3910-2	3.557713838
HGLibA_49290	THAP10	3.554841425
HGLibA_21878	HOOK2	3.551972687
HGLibA_62235	hsa-mir-5680	3.549107616
HGLibA_45204	SLC4A10	3.546246206
HGLibA_39324	PTGES	3.543388448
HGLibA_35309	PAX6	3.540534335
HGLibA_56476	ZNF660	3.522878745
HGLibA_53536	VASN	3.522878745
HGLibA_24336	KCNK9	3.397940009
HGLibB_33678	OR2A12	3.397940009
HGLibB_23307	INPP4B	3.304693478
HGLibB_45133	SLC46A2	3.301029996
HGLibB_13795	DPCR1	3.301029996
HGLibB_05249	C12orf49	3.22184875
HGLibB_04041	B9D1	3.15490196
HGLibB_55914	ZNF431	3.15490196
HGLibB_55334	ZMYM5	3.143210711
HGLibA_54484	XAGE3	3.113588245
HGLibA_47807	SUPT20HL1	3.096910013
HGLibA_46408	SPANXN3	3.096910013
HGLibA_47241	ST3GAL2	3.045757491
HGLibB_29935	MRPL4	3.045757491
HGLibA_30988	NAA30	2.958607315
HGLibB_34103	OR52B4	2.920818754
HGLibB_24093	KCNA10	2.886056648
HGLibA_57472	hsa-mir-1262	2.857372801
HGLibB_29771	MRAS	2.853871964
HGLibB_14096	DUS2L	2.853871964
HGLibB_55769	ZNF34	2.82627193
HGLibA_54087	WDR41	2.823908741
HGLibB_40899	RFTN1	2.795880017
HGLibA_58018	hsa-mir-18a	2.795880017
HGLibA_24344	KCNMB2	2.769551079
HGLibA_23388	INSL5	2.769551079
HGLibB_36343	PHF8	2.721246399
HGLibB_19675	GP1BB	2.721246399

HGLibB_46362	SPARC	2.698970004
HGLibA_50186	TMEM203	2.698970004
HGLibA_52245	TUBA1B	2.677780705
HGLibA_12152	CYP39A1	2.677780705
HGLibB_55012	ZDHHC14	2.657577319
HGLibB_29521	MNAT1	2.657577319
HGLibB_35896	PDS5A	2.638272164
HGLibB_21744	HNF1A	2.638272164
HGLibB_44357	SLC15A3	2.619788758
HGLibA_42421	RTP1	2.619788758
HGLibB_44400	SLC16A8	2.602059991
HGLibB_30349	MTHFS	2.602059991
HGLibB_11026	CPXM2	2.585026652
HGLibA_16387	FAM198A	2.585026652
HGLibB_05474	C16orf72	2.568636236
HGLibB_20675	GYPC	2.568636236
HGLibA_26606	LIPE	2.567450784
HGLibB_56930	ZSCAN20	2.552841969
HGLibA_43578	SERPINE1	2.552841969
HGLibA_35155	PAPSS2	2.537602002
HGLibA_26547	LIMS1	2.522878745
HGLibB_15337	EPPIN-WFDC6	2.521733719
HGLibA_51552	TRIM58	2.508852704
HGLibA_24990	KIR3DL1	2.508638306
HGLibA_37855	PPBP	2.508638306
HGLibB_19366	GMCL1	2.508459022
HGLibA_45002	SLC35F1	2.495425843
HGLibB_13754	DOK4	2.494850022
HGLibA_16742	FAM83B	2.494850022
HGLibA_15463	ERG	2.482294864
HGLibB_09401	CHD9	2.48148606
HGLibA_21339	HIPK1	2.48148606
HGLibA_55477	ZNF138	2.455931956
HGLibA_09811	CLCA4	2.455931956
HGLibA_08686	CDH6	2.45037151
HGLibB_07970	CCL16	2.443697499
HGLibA_54388	WNT8A	2.443697499
HGLibA_47379	STARD9	2.431798276
HGLibB_07747	CCDC30	2.420216403
HGLibA_36485	PI16	2.418749141
HGLibA_61949	hsa-mir-548az	2.408935393
HGLibA_45881	SNAP29	2.408935393
HGLibB_40117	RALGPS1	2.397940009
HGLibA_33836	OR2M3	2.387216143
HGLibB_54999	ZDHHC1	2.37675071

HGLibA_55095	ZDHHC2	2.37675071
HGLibA_49610	TLE2	2.366531544
HGLibB_09994	CLEC6A	2.366531544
HGLibA_31779	NFATC2IP	2.358169082
HGLibB_39811	RAB28	2.356547324
HGLibB_37251	PMEPA1	2.356547324
HGLibB_33794	OR2M4	2.352182952
HGLibA_31818	NFKB2	2.346787486
HGLibA_49650	TLR3	2.346787486
HGLibA_25684	KRTAP19-8	2.34530858
HGLibB_33878	OR4A15	2.337242168
HGLibA_45366	SLC7A4	2.337242168
HGLibA_41026	RGPD1	2.327902142
HGLibA_56766	ZNF791	2.318758763
HGLibA_52907	UGT2A2	2.318758763
HGLibA_27165	LRFN5	2.30980392
HGLibB_34638	OSGEPL1	2.30980392
HGLibA_30369	MTG1	2.292429824
HGLibA_49610	TLE2	2.283996656
HGLibA_04968	BTN3A1	2.283996656
HGLibA_13936	DPYSL5	2.27572413
HGLibA_07629	CCDC146	2.27572413
HGLibA_56469	ZNF655	2.26760624
HGLibA_36531	PIDD	2.26760624
HGLibA_56214	ZNF551	2.259637311
HGLibB_40832	REV1	2.259637311
HGLibB_15753	EXOSC2	2.251811973
HGLibB_42863	SCGB1D2	2.244125144
HGLibA_32730	NRTN	2.244125144
HGLibA_57005	ZSCAN22	2.236572006
HGLibA_57845	hsa-mir-145	2.236572006
HGLibA_57475	hsa-mir-1263	2.229147988
HGLibB_34896	PABPN1	2.22184875
HGLibB_19063	GIMAP7	2.22184875
HGLibA_58076	hsa-mir-193b	2.214670165
HGLibA_35076	PALM3	2.214670165
HGLibB_45143	SLC47A2	2.200659451
HGLibB_12041	CYP11B1	2.200659451
HGLibB_41536	RNF183	2.193820026
HGLibB_08932	CEACAM3	2.193820026
HGLibA_30090	MRPS31	2.180456064
HGLibB_01937	ANK3	2.173925197
HGLibA_61922	hsa-mir-548as	2.173925197
HGLibA_18539	GALNT13	2.167491087
HGLibA_55673	ZNF253	2.161150909

HGLibA_53875	VSX1	2.161150909
HGLibA_61350	hsa-mir-487b	2.15490196
HGLibA_09882	CLDN22	2.15490196
HGLibA_45893	SNAPC2	2.148741651
HGLibA_11633	CTIF	2.148741651
HGLibA_60385	hsa-mir-4492	2.142667504
HGLibA_09921	CLEC10A	2.142667504
HGLibB_40065	RAE1	2.13076828
HGLibB_24881	KIF26A	2.13076828
HGLibB_19310	GLT6D1	2.124938737
HGLibB_16123	FAM13B	2.124938737
HGLibA_24426	KCTD14	2.122530171
HGLibB_43384	2-Sep	2.119186408
HGLibB_19951	GPR179	2.119186408
HGLibB_18057	FSD1L	2.113509275
HGLibB_55432	ZNF148	2.107905397
HGLibB_31814	NFYB	2.107905397
HGLibB_47819	SV2B	2.102372909
HGLibA_59689	hsa-mir-412	2.102372909
HGLibA_34032	OR4K2	2.096910013
HGLibA_43286	SELE	2.096910013
HGLibA_05768	C1orf131	2.091514981
HGLibB_49563	TLK1	2.086186148
HGLibA_50034	TMEM164	2.086186148
HGLibA_55843	ZNF343	2.075720714
HGLibA_54058	WDR27	2.075720714
HGLibA_50496	TMEM68	2.070581074
HGLibA_34390	OR6C4	2.070581074
HGLibB_26178	LDB2	2.065501549
HGLibA_43766	SFTPA1	2.065501549
HGLibA_34390	OR6C4	2.060480747
HGLibA_07086	CAMK4	2.060480747
HGLibB_21555	HK2	2.055517328
HGLibA_08152	CCR3	2.055517328
HGLibB_50695	TNFRSF13C	2.050609993
HGLibB_27577	LTV1	2.050609993
HGLibA_20079	GPR75	2.040958608
HGLibA_17233	FCRL4	2.040958608
HGLibA_00831	ADAMTS4	2.036212173
HGLibA_21857	HNRPLL	2.036212173
HGLibB_23854	IYD	2.031517051
HGLibA_22669	IFNA16	2.026872146
HGLibB_34629	OSCP1	2.022276395
HGLibA_61088	hsa-mir-4748	2.022276395
HGLibA_32600	NR2C2AP	2.017728767

HGLibA_36715	PIP5K1A	2.013228266
HGLibA_32569	NR0B1	2.013228266
HGLibB_23219	IMPACT	2.008773924
HGLibA_37808	PPA2	2.008773924
HGLibB_09723	CISD3	2.004364805
HGLibB_06828	CAB39	2.004364805
HGLibB_30111	MS4A4A	2
HGLibB_48866	TEAD1	2
HGLibA_30380	MTHFD1L	1.995678626
HGLibA_30968	N6AMT2	1.991399828
HGLibA_33903	OR2W1	1.987162775
HGLibA_38081	PPP1R26	1.985897997
HGLibA_30202	MSH6	1.985433262
HGLibB_02813	ARHGEF12	1.982966661
HGLibA_40370	RASL12	1.982966661
HGLibB_23044	IL20RA	1.978810701
HGLibA_26414	LGR6	1.978810701
HGLibB_30264	MT3	1.974694135
HGLibA_41936	RPL31	1.974694135
HGLibB_19782	GPIHBP1	1.970616222
HGLibA_47036	SRPX2	1.970616222
HGLibB_16693	FAM73A	1.966576245
HGLibB_12187	CYP4V2	1.962573502
HGLibB_42947	SCN7A	1.958607315
HGLibB_41122	RHOBTB1	1.958607315
HGLibA_11764	CUBN	1.954677021
HGLibB_32732	NSMF	1.954677021
HGLibB_24423	KCTD5	1.950781977
HGLibA_59348	hsa-mir-3690-2	1.946921557
HGLibA_27756	LYRM5	1.946921557
HGLibB_21949	HOXC8	1.943095149
HGLibB_40400	RBFA	1.943095149
HGLibB_31281	NCKIPSD	1.939210023
HGLibB_21990	HPCAL1	1.935542011
HGLibA_06408	C5orf54	1.935542011
HGLibB_15278	EPHA5	1.931814138
HGLibA_34125	OR51M1	1.931814138
HGLibB_22073	HRH4	1.928117993
HGLibA_38992	PRX	1.928117993
HGLibA_10212	CMTM2	1.924453039
HGLibB_28477	MBOAT1	1.924453039
HGLibA_49823	TMED7-TICAM2	1.920818754
HGLibB_18750	GCG	1.91721463
HGLibA_63742	hsa-mir-6889	1.91721463
HGLibA_34501	OR8B4	1.913640169

HGLibB_31483	NDUFB6	1.910094889
HGLibA_21053	HDHD2	1.910094889
HGLibA_45147	SLC43A1	1.906578315
HGLibB_08484	CD99_Y	1.906578315
HGLibB_02466	APOBEC1	1.90517543
HGLibA_26908	LOC283403	1.903089987
HGLibB_27641	LY75-CD302	1.903089987
HGLibA_54810	ZBTB1	1.899629455
HGLibB_46048	SNX7	1.896196279
HGLibA_41182	RHOC	1.896196279
HGLibB_39146	PSME3	1.89279003
HGLibA_31064	NAGA	1.89279003
HGLibB_09443	CHID1	1.88941029
HGLibA_50063	TMEM173	1.88941029
HGLibB_53315	USP47	1.886056648
HGLibB_08170	CCRL2	1.886056648
HGLibA_57371	hsa-mir-1244-3	1.882728704
HGLibB_56779	ZNF837	1.879426069
HGLibA_52986	UNC119	1.879426069
HGLibB_23243	INCA1	1.876148359
HGLibB_03778	ATP8B1	1.876148359
HGLibB_06076	C22orf43	1.872895202
HGLibB_35697	PDCD7	1.872895202
HGLibB_39841	RAB36	1.869666232
HGLibB_38826	PRRG3	1.866461092
HGLibA_11950	CXorf58	1.863279433
HGLibB_40268	RASD1	1.860120914
HGLibA_24699	KIAA1147	1.860120914
HGLibB_00931	ADCY9	1.8569852
HGLibB_33817	OR2T2	1.8569852
HGLibA_30006	MRPL49	1.853871964
HGLibB_30908	N4BP1	1.847711656
HGLibB_20134	GPSM3	1.847711656
HGLibB_55181	ZFPL1	1.844663963
HGLibA_54369	WNT4	1.844663963
HGLibB_35836	PDIA5	1.841637508
HGLibB_15159	ENPP6	1.841637508
HGLibB_23043	IL20	1.838631998
HGLibA_39805	RAB11FIP5	1.838631998
HGLibB_55151	ZFP42	1.835647144
HGLibA_55020	ZCCHC17	1.835647144
HGLibA_48093	SYT9	1.832682665
HGLibA_62018	hsa-mir-548i-2	1.832682665
HGLibA_55282	ZFYVE21	1.829738285
HGLibB_13404	DMRT3	1.826813732

HGLibA_21211	HEXIM1	1.826813732
HGLibA_39242	PSTPIP2	1.823908741
HGLibA_23743	ITGA7	1.823908741
HGLibB_08251	CD180	1.821023053
HGLibA_28709	ME1	1.821023053
HGLibA_28497	MBIP	1.818156412
HGLibB_45943	SNTB1	1.812479279
HGLibA_33514	OR10H4	1.812479279
HGLibA_42976	SCN1B	1.809668302
HGLibA_41301	RINL	1.809668302
HGLibB_39743	RAB11FIP1	1.806875402
HGLibA_01866	ANAPC10	1.806875402
HGLibB_56221	ZNF574	1.804100348
HGLibB_36969	PLCL2	1.804100348
HGLibA_56543	ZNF688	1.801342913
HGLibB_02677	ARFIP2	1.801342913
HGLibA_25684	KRTAP19-8	1.798602876
HGLibA_63449	hsa-mir-6818	1.795880017
HGLibA_48940	TECPR1	1.795880017
HGLibB_18545	GALNT7	1.790484985
HGLibA_34406	OR6C74	1.790484985
HGLibB_29028	MFN1	1.785156152
HGLibB_47285	STAP1	1.785156152
HGLibA_18902	GDPD5	1.782516056
HGLibA_15711	EVX2	1.779891912
HGLibA_07035	CALML4	1.779891912
HGLibA_39075	PSMA3	1.777283529
HGLibA_28125	MAP1LC3B	1.777283529
HGLibA_12614	DDIT3	1.774690718
HGLibB_55612	ZNF256	1.774690718
HGLibA_31770	NFAT5	1.772113295
HGLibA_54502	XDH	1.769551079
HGLibA_19355	GLTSCR1L	1.769551079
HGLibB_36046	PEX12	1.76700389
HGLibB_24170	KCNG3	1.76700389
HGLibA_35653	PCOLCE	1.764471553
HGLibB_55684	ZNF286A	1.761953897
HGLibA_36241	PGK1	1.761953897
HGLibA_50337	TMEM256	1.759450752
HGLibA_06403	C5orf52	1.756961951
HGLibA_34731	OTOL1	1.754487332
HGLibA_18379	GABRB3	1.754487332
HGLibA_17921	FOXO1	1.752026734
HGLibA_62747	hsa-mir-627	1.752026734
HGLibA_55316	ZHX2	1.749579998

HGLibB_36749	PKD1L3	1.747146969
HGLibA_64334	hsa-mir-940	1.744727495
HGLibB_23119	IL36G	1.742321425
HGLibB_17010	FBXO16	1.742321425
HGLibB_54869	ZC2HC1A	1.739928612
HGLibB_46022	SNX29	1.739928612
HGLibB_39824	RAB31	1.73754891
HGLibA_18685	GATAD2A	1.73754891
HGLibB_24563	KIAA0146	1.735182177
HGLibA_23372	INSC	1.735182177
HGLibA_30423	MTMR2	1.730487056
HGLibA_11935	CXorf49B	1.730487056
HGLibA_00733	ADAM12	1.728158393
HGLibB_19014	GHR	1.725842151
HGLibA_18815	GCNT3	1.725842151
HGLibB_16273	FAM177A1	1.723538196
HGLibA_32550	NPY1R	1.723538196
HGLibB_28590	MCMBP	1.721246399
HGLibA_19873	GPR123	1.721246399
HGLibB_36041	PEX11B	1.718966633
HGLibA_03572	ATP13A3	1.718966633
HGLibB_30111	MS4A4A	1.716698771
HGLibA_36816	PKHD1L1	1.716698771
HGLibB_21667	HMGB3	1.714442691
HGLibA_03886	AUTS2	1.714442691
HGLibA_06589	C7orf73	1.709965389
HGLibA_04008	B3GNTL1	1.709965389
HGLibB_40107	RALGAPA2	1.707743929
HGLibA_54078	WDR37	1.707743929
HGLibB_32878	NUDCD1	1.705533774
HGLibB_00223	ABCG5	1.705533774
HGLibA_50578	TMF1	1.70333481
HGLibA_19804	GPHN	1.70333481
HGLibB_54792	ZBTB32	1.701146924
HGLibA_55285	ZFYVE26	1.701146924
HGLibB_03110	ARRDC1	1.698970004
HGLibA_59350	hsa-mir-3690-2	1.698970004
HGLibA_44090	SHROOM2	1.692503962
HGLibA_42196	RPSA	1.692503962
HGLibA_57040	ZSWIM2	1.690369833
HGLibA_52692	UBE4B	1.690369833
HGLibB_19250	GLIS2	1.688246139
HGLibB_18009	FRMPD1	1.688246139
HGLibB_34426	OR7D2	1.68613278
HGLibB_19345	GLYAT	1.68613278

HGLibB_49488	TINAG	1.684029655
HGLibB_18348	GABRB1	1.684029655
HGLibB_19668	GOT2	1.681936665
HGLibA_07909	CCDC88B	1.681936665
HGLibB_21734	HMX2	1.679853714
HGLibB_20221	GRHL1	1.679853714
HGLibB_43891	SH3D21	1.677780705
HGLibA_60367	hsa-mir-4487	1.677780705
HGLibB_16920	FBLN1	1.675717545
HGLibA_16581	FAM3C	1.675717545
HGLibB_41782	RPGR	1.673664139
HGLibB_31781	NFKBIB	1.673664139
HGLibA_55841	ZNF341	1.671620397
HGLibA_44374	SLC12A7	1.671620397
HGLibA_58796	hsa-mir-3179-3	1.669586227
HGLibA_58983	hsa-mir-329-2	1.669586227
HGLibA_12360	DAZ4	1.66756154
HGLibB_13744	DOHH	1.665546249
HGLibA_35955	PDX1	1.665546249
HGLibA_52229	TTR	1.663540266
HGLibA_03561	ATP11C	1.663540266
HGLibA_61646	hsa-mir-5189	1.661543506
HGLibA_18014	FRMD4A	1.661543506
HGLibB_39982	RABL3	1.659555885
HGLibA_13493	DNAH6	1.659555885
HGLibA_53416	USP9X	1.657577319
HGLibA_40859	RERG	1.657577319
HGLibA_39909	RAB3B	1.655607726
HGLibA_24592	KIAA0141	1.655607726
HGLibB_46122	SORBS2	1.653647026
HGLibB_08083	CCNF	1.653647026
HGLibB_42961	SCNN1A	1.651695137
HGLibA_01731	ALS2CR8	1.651695137
HGLibB_39525	PTX4	1.649751982
HGLibA_63022	hsa-mir-6715b	1.649751982
HGLibA_18389	GABRG1	1.647817482
HGLibA_50071	TMEM176A	1.645891561
HGLibB_01643	ALKBH1	1.645891561
HGLibA_35919	PDLIM5	1.643974143
HGLibA_50455	TMEM57	1.642065153
HGLibA_29621	MOG	1.640164518
HGLibA_53794	VPS45	1.638272164
HGLibA_25861	KRTDAP	1.638272164
HGLibA_23705	ITFG1	1.63638802
HGLibB_57050	NonTargetingContrc	1.634512015

HGLibB_13063	DGKQ	1.634512015
HGLibA_38124	PPP1R8	1.632644079
HGLibB_15276	EPHA5	1.628932138
HGLibA_05732	C1orf101	1.628932138
HGLibB_04010	B4GALNT1	1.627087997
HGLibA_40194	RANBP10	1.627087997
HGLibA_44123	SIGLEC1	1.625251654
HGLibA_33188	NYX	1.625251654
HGLibB_32681	NRSN1	1.623423043
HGLibA_41269	RIMBP3C	1.623423043
HGLibB_55141	ZFP36L1	1.621602099
HGLibB_14454	EDN3	1.621602099
HGLibA_34341	OR5M8	1.619788758
HGLibA_08871	CDRT1	1.619788758
HGLibB_44704	SLC25A47	1.617982957
HGLibA_62318	hsa-mir-5696	1.617982957
HGLibA_25382	KPTN	1.616184634
HGLibA_05097	C10orf82	1.616184634
HGLibB_27047	LPHN1	1.614393726
HGLibB_55130	ZFP3	1.614393726
HGLibB_03431	ATF2	1.612610174
HGLibB_45315	SLC7A4	1.610833916
HGLibB_12946	DENND6A	1.610833916
HGLibB_09476	CHMP3	1.609064893
HGLibB_13859	DPP8	1.607303047
HGLibA_13958	DRD1	1.607303047
HGLibA_44300	SLA	1.605548319
HGLibA_33134	NXF3	1.605548319
HGLibB_41982	RPP30	1.603800653
HGLibB_30001	MRPS15	1.603800653
HGLibA_57295	hsa-mir-1224	1.602059991
HGLibA_11375	CSN2	1.602059991
HGLibB_56635	ZNF77	1.600326279
HGLibA_53226	USP17L15	1.598599459
HGLibB_03108	ARRB2	1.596879479
HGLibA_31064	NAGA	1.596879479
HGLibB_46013	SNX24	1.595166283
HGLibA_25361	KPNA3	1.595166283
HGLibB_20145	GPX2	1.59345982
HGLibB_17374	FGF21	1.59345982
HGLibA_58792	hsa-mir-3179-2	1.591760035
HGLibA_50437	TMEM53	1.591760035
HGLibB_43923	SH3RF3	1.590066877
HGLibB_07505	CCDC102B	1.590066877
HGLibB_19647	GORAB	1.588380294

HGLibA_39182	PSMD7	1.588380294
HGLibA_24624	KIAA0319L	1.586700236
HGLibA_24201	KCNG2	1.586700236
HGLibB_09570	CHRNA6	1.583359493
HGLibA_58337	hsa-mir-23c	1.583359493
HGLibA_33643	OR1A2	1.581698709
HGLibA_63154	hsa-mir-675	1.580044252
HGLibA_33606	OR13C5	1.580044252
HGLibB_33395	OPN5	1.578396073
HGLibA_42239	RRAS2	1.578396073
HGLibB_25755	KRTAP5-2	1.576754126
HGLibA_28073	MAN2A2	1.576754126
HGLibB_48456	TBC1D25	1.575118363
HGLibA_34032	OR4K2	1.575118363
HGLibB_01120	AFM	1.573488739
HGLibA_56893	ZNF880	1.573488739
HGLibA_21139	HEPACAM	1.57024772
HGLibB_25921	LAMB2	1.568636236
HGLibA_43008	SCN9A	1.568636236
HGLibA_62920	hsa-mir-6512	1.567030709
HGLibA_17599	FKTN	1.567030709
HGLibB_00618	ACTL6A	1.565431096
HGLibB_13872	DPPA4	1.565431096
HGLibB_56901	ZPLD1	1.563837353
HGLibA_52229	TTR	1.562249437
HGLibB_39863	RAB3C	1.560667306
HGLibB_00236	ABHD11	1.560667306
HGLibB_16050	FAM122C	1.559090918
HGLibA_54678	YIPF4	1.559090918
HGLibA_48437	TAT	1.557520231
HGLibA_25372	KPNA7	1.557520231
HGLibB_28268	MAPKBP1	1.555955204
HGLibB_43457	SERPINA11	1.555955204
HGLibB_09723	CISD3	1.554395797
HGLibB_38477	PRKAR2A	1.552841969
HGLibB_34123	OR52H1	1.552841969
HGLibA_35530	PCDHGA2	1.55129368
HGLibA_27767	LYSMD2	1.55129368
HGLibA_49247	TGIF2LX	1.549750892
HGLibA_18610	GAP43	1.549750892
HGLibA_46264	SOX4	1.548213564
HGLibA_15567	ESF1	1.548213564
HGLibB_55110	ZFHX4	1.54668166
HGLibB_18348	GABRB1	1.54668166
HGLibA_04553	BLNK	1.54515514

HGLibB_31781	NFKBIB	1.543633967
HGLibB_10881	CPA4	1.543633967
HGLibB_48666	TCEAL8	1.542118103
HGLibA_55890	ZNF383	1.542118103
HGLibB_25693	KRTAP25-1	1.540607512
HGLibA_37625	POLR3F	1.540607512
HGLibA_57184	hsa-mir-106a	1.539102157
HGLibA_39565	PTTG1IP	1.539102157
HGLibB_03927	AZGP1	1.537602002
HGLibA_29825	MREG	1.537602002
HGLibB_26683	LMOD2	1.536107011
HGLibA_38222	PPP6R1	1.536107011
HGLibB_30849	MYOM2	1.534617149
HGLibA_29907	MRPL15	1.534617149
HGLibA_60564	hsa-mir-4526	1.53165267
HGLibA_59728	hsa-mir-4255	1.53165267
HGLibA_60811	hsa-mir-4674	1.530177984
HGLibA_49626	TLK2	1.530177984
HGLibB_54778	ZBTB22	1.528708289
HGLibA_55156	ZFAND4	1.528708289
HGLibA_33557	OR10X1	1.527243551
HGLibA_02449	APOA1	1.527243551
HGLibB_01227	AGPAT5	1.525783736
HGLibA_32159	NME3	1.525783736
HGLibB_41382	RNASEH2C	1.522878745
HGLibA_50046	TMEM168	1.521433504
HGLibA_51913	TSPAN17	1.518557371
HGLibA_47420	STEAP1	1.518557371
HGLibB_16050	FAM122C	1.515700161
HGLibB_02706	ARHGAP12	1.514278574
HGLibA_60796	hsa-mir-4670	1.512861625
HGLibA_34029	OR4K17	1.512861625
HGLibB_46723	SPPL2C	1.511449283
HGLibA_37881	PPFIA4	1.511449283
HGLibA_47173	SSTR1	1.510041521
HGLibA_15845	F11	1.510041521
HGLibA_60171	hsa-mir-4442	1.508638306
HGLibA_25653	KRTAP15-1	1.507239611
HGLibB_45133	SLC46A2	1.501689446
HGLibB_01726	ALS2CR12	1.501689446
HGLibB_55812	ZNF366	1.500312917
HGLibA_53578	VCPIP1	1.500312917
HGLibA_50470	TMEM62	1.498940738
HGLibA_15170	ENPP2	1.498940738
HGLibB_12464	DCD	1.49757288

HGLibA_29577	MNT	1.49757288
HGLibB_47874	SYCP2L	1.496209317
HGLibA_22895	IGSF5	1.496209317
HGLibB_46468	SPATA6L	1.493494968
HGLibB_40413	RBKS	1.493494968
HGLibA_54692	YJEFN3	1.492144128
HGLibB_20040	GPR63	1.492144128
HGLibA_19223	GLB1L2	1.48945499
HGLibA_56777	ZNF799	1.4867824
HGLibA_53082	UPK3BL	1.4867824
HGLibA_57744	hsa-mir-1307	1.485452247
HGLibB_50639	TNC	1.485452247
HGLibA_46701	SPINK6	1.484126156
HGLibB_35733	PDE3A	1.482804102
HGLibA_64319	hsa-mir-936	1.482804102
HGLibB_56194	ZNF566	1.48148606
HGLibA_60385	hsa-mir-4492	1.48148606
HGLibB_05249	C12orf49	1.480172006
HGLibB_26803	LOC100289561	1.480172006
HGLibA_37202	PLLP	1.477555766
HGLibB_34736	OTX1	1.476253533
HGLibA_47205	SSX2IP	1.476253533
HGLibA_54250	WFDC10B	1.474955193
HGLibA_33963	OR4C6	1.474955193
HGLibB_02466	APOBEC1	1.473660723
HGLibA_61801	hsa-mir-527	1.473660723
HGLibA_61426	hsa-mir-5006	1.472370099
HGLibA_26453	LHX3	1.472370099
HGLibA_05245	C12orf43	1.4710833
HGLibB_12760	DECR1	1.4710833
HGLibA_22253	HSP90AB1	1.469800302
HGLibB_36929	PLBD2	1.468521083
HGLibA_43622	SESN2	1.468521083
HGLibA_25179	KLHL30	1.467245621
HGLibB_36979	PLCXD2	1.46470588
HGLibB_27832	MAFA	1.46470588
HGLibB_41221	RIMKLB	1.462180905
HGLibA_40524	RBM27	1.460923901
HGLibB_15467	ERICH1	1.459670525
HGLibA_60171	hsa-mir-4442	1.459670525
HGLibB_21990	HPCAL1	1.458420756
HGLibB_08352	CD36	1.457174573
HGLibA_31710	NEU1	1.455931956
HGLibA_24919	KIF26B	1.455931956
HGLibB_48266	TAPBPL	1.454692884

HGLibA_46750	SPOCK2	1.454692884
HGLibB_28113	MAP2K6	1.452225295
HGLibA_31754	NEXN	1.452225295
HGLibB_42116	RPS6KA5	1.450996738
HGLibA_47850	SUSD3	1.450996738
HGLibB_41676	RNPEP	1.449771647
HGLibA_39639	PWWP2A	1.449771647
HGLibB_17910	FOXP2	1.448550002
HGLibA_31971	NIPSNAP1	1.448550002
HGLibB_08643	CDH17	1.447331784
HGLibB_26509	LIMS1	1.447331784
HGLibA_02104	ANKRD45	1.446116973
HGLibA_19740	GPATCH2L	1.444905551
HGLibA_02916	ARIH2	1.443697499
HGLibB_43019	SCUBE2	1.442492798
HGLibA_20929	HBG2	1.442492798
HGLibA_25509	KRT5	1.441291429
HGLibA_20053	GPR55	1.441291429
HGLibB_43615	SETDB1	1.440093375
HGLibA_55664	ZNF25	1.440093375
HGLibB_56594	ZNF740	1.438898616
HGLibA_53311	USP25	1.438898616
HGLibA_50262	TMEM232	1.437707136
HGLibA_27426	LRRC73	1.437707136
HGLibB_56039	ZNF500	1.436518915
HGLibA_12416	DCAF11	1.436518915
HGLibA_56063	ZNF474	1.435333936
HGLibB_40124	RALY	1.435333936
HGLibA_56868	ZNF85	1.434152181
HGLibA_46685	SPINK13	1.432973634
HGLibB_17868	FOXJ3	1.432973634
HGLibA_22541	IDH2	1.431798276
HGLibB_17167	FCGR2A	1.43062609
HGLibA_40905	RFC2	1.43062609
HGLibA_45387	SLC8A1	1.42945706
HGLibB_02579	AQP10	1.42945706
HGLibA_56188	ZNF540	1.428291168
HGLibB_17637	FLRT3	1.427128398
HGLibA_40333	RASGEF1B	1.427128398
HGLibA_45520	SLITRK3	1.425968732
HGLibA_23958	JMJD6	1.425968732
HGLibA_28356	MARCKS	1.424812155
HGLibA_56944	ZNRF3	1.424812155
HGLibA_52245	TUBA1B	1.42365865
HGLibA_58254	hsa-mir-215	1.4225082

HGLibA_27879	MAFF	1.4225082
HGLibB_55230	ZG16	1.42136079
HGLibA_54768	ZADH2	1.42136079
HGLibB_10022	CLIC4	1.420216403
HGLibB_00604	ACTBL2	1.420216403
HGLibB_36849	PLA2G3	1.419075024
HGLibB_24838	KIF17	1.417936637
HGLibB_22257	HSPA4L	1.415668776
HGLibB_42281	RSPH4A	1.41453927
HGLibB_14360	ECE1	1.41453927
HGLibB_44927	SLC35D2	1.413412695
HGLibB_17378	FGF23	1.413412695
HGLibB_31387	NDST2	1.412289035
HGLibA_34326	OR5L2	1.412289035
HGLibA_51926	TSPAN3	1.411168274
HGLibA_45606	SMARCA2	1.411168274
HGLibB_00490	ACOT8	1.410050399
HGLibA_49673	TLX1NB	1.410050399
HGLibA_38230	PPRC1	1.407823243
HGLibA_03244	ASB6	1.407823243
HGLibB_26640	LMBRD2	1.406713933
HGLibA_26641	LIX1	1.406713933
HGLibB_29775	MRC1	1.40560745
HGLibB_20724	H2AFB3	1.40560745
HGLibB_42661	SAP130	1.404503778
HGLibA_49114	TF	1.404503778
HGLibB_39620	PYCARD	1.403402904
HGLibA_23078	IL20RB	1.403402904
HGLibB_06993	CALCOCO1	1.402304814
HGLibA_44300	SLA	1.402304814
HGLibB_18142	FUNDC2	1.401209493
HGLibA_61965	hsa-mir-548d-2	1.401209493
HGLibA_34495	OR8B2	1.400116928
HGLibB_16883	FASTKD3	1.399027104
HGLibB_40220	RARA	1.399027104
HGLibA_02939	ARL14EP	1.397940009
HGLibB_35632	PCSK5	1.396855627
HGLibB_29119	MGLL	1.396855627
HGLibB_02677	ARFIP2	1.395773947
HGLibB_01812	AMOT	1.395773947
HGLibB_20909	HBQ1	1.394694954
HGLibA_53548	VAV1	1.394694954
HGLibB_26365	LGI4	1.393618635
HGLibA_13517	DNAJA3	1.393618635
HGLibA_52208	TTLL5	1.392544977

HGLibA_41750	ROGDI	1.392544977
HGLibA_62510	hsa-mir-604	1.391473966
HGLibA_43286	SELE	1.391473966
HGLibA_30105	MRPS5	1.390405591
HGLibA_18064	FSCB	1.390405591
HGLibA_26174	LCN15	1.389339837
HGLibA_23840	ITPK1	1.389339837
HGLibB_44688	SLC25A42	1.388276692
HGLibA_27216	LRP2BP	1.388276692
HGLibA_42951	SCLT1	1.387216143
HGLibA_06041	C21orf58	1.387216143
HGLibB_05695	C19orf70	1.386158178
HGLibA_44606	SLC23A1	1.385102784
HGLibA_04087	BAG5	1.384049948
HGLibB_36432	PHYKPL	1.382999659
HGLibB_27477	LSM1	1.382999659
HGLibB_44068	SIGIRR	1.381951903
HGLibB_28710	MED13L	1.381951903
HGLibB_02844	ARHGEF3	1.380906669
HGLibA_11389	CSNK1E	1.380906669
HGLibB_10974	CPPED1	1.379863945
HGLibA_41386	RMND5B	1.379863945
HGLibB_42661	SAP130	1.378823718
HGLibB_09518	CHPF2	1.378823718
HGLibB_29669	MPDU1	1.377785977
HGLibA_05566	C17orf72	1.377785977
HGLibA_26870	LOC100507462	1.37675071
HGLibA_23488	IQCA1	1.37675071
HGLibA_21933	HOXB2	1.375717904
HGLibA_21250	HGSNAT	1.375717904
HGLibB_37989	PPP1R14B	1.374687549
HGLibB_47836	SVOP	1.374687549
HGLibB_04211	BBS12	1.373659633
HGLibA_23362	INPP5K	1.372634143
HGLibA_18964	GFRA1	1.372634143
HGLibA_59878	hsa-mir-4292	1.37161107
HGLibB_33080	NWD1	1.37161107
HGLibA_48019	SYNPO	1.370590401
HGLibB_54070	WDR6	1.369572125
HGLibA_55776	ZNF304	1.369572125
HGLibB_02896	ARID3C	1.368556231
HGLibA_41714	RNLS	1.368556231
HGLibA_45427	SLC9A8	1.367542708
HGLibA_40965	RFX2	1.367542708
HGLibB_23955	JPH4	1.366531544

HGLibA_14339	EAPP	1.366531544
HGLibB_33724	OR2B3	1.36552273
HGLibA_05760	C1orf122	1.36552273
HGLibB_56510	ZNF701	1.364516253
HGLibA_02745	ARHGAP27	1.364516253
HGLibA_52148	TTC5	1.363512104
HGLibA_04015	B4GALNT2	1.36251027
HGLibB_02327	AP3M1	1.361510743
HGLibA_57225	hsa-mir-1183	1.361510743
HGLibB_08504	CDC14B	1.360513511
HGLibA_26180	LCN6	1.360513511
HGLibB_37883	PPIG	1.359518563
HGLibA_38124	PPP1R8	1.359518563
HGLibA_01029	ADPRHL1	1.358525889
HGLibB_47809	SUV420H1	1.356547324
HGLibA_46110	SNX9	1.356547324
HGLibA_36927	PLA2R1	1.355561411
HGLibB_07859	CCDC74B	1.354577731
HGLibB_39811	RAB28	1.353596274
HGLibB_02789	ARHGAP9	1.35261703
HGLibB_00987	ADK	1.35261703
HGLibB_50437	TMEM69	1.351639989
HGLibA_41386	RMND5B	1.350665141
HGLibB_36041	PEX11B	1.350665141
HGLibB_01165	AGBL1	1.349692477
HGLibA_48492	TBC1D19	1.349692477
HGLibA_28557	MCAM	1.348721986
HGLibB_35778	PDE7B	1.348721986
HGLibA_09202	CERS2	1.347753659
HGLibA_55482	ZNF140	1.347753659
HGLibB_54790	ZBTB3	1.346787486
HGLibA_24184	KCNE1	1.345823458
HGLibB_11311	CSF1R	1.345823458
HGLibB_04223	BBS7	1.344861565
HGLibB_39579	PVRL3	1.344861565
HGLibA_42437	RUFY1	1.343901798
HGLibA_49673	TLX1NB	1.343901798
HGLibB_20601	GTSF1	1.342944147
HGLibA_56411	ZNF622	1.341035157
HGLibB_01850	AMY2B	1.341035157
HGLibA_26465	LHX8	1.3400838
HGLibB_43653	SF3B2	1.3400838
HGLibB_27047	LPHN1	1.339134522
HGLibA_54966	ZC3H15	1.339134522
HGLibA_44259	SIX6	1.338187314

HGLibB_55505	ZNF202	1.338187314
HGLibA_53911	VWA5B2	1.337242168
HGLibB_41337	RNASE1	1.337242168
HGLibA_50764	TNFRSF17	1.336299075
HGLibA_46252	SOX2	1.336299075
HGLibA_38412	PRDX3	1.335358024
HGLibB_48709	TCF19	1.335358024
HGLibA_05621	C18orf63	1.334419009
HGLibA_37795	POU6F1	1.334419009
HGLibA_34883	P2RY14	1.333482019
HGLibA_31555	NDUFS4	1.333482019
HGLibA_36859	PKP3	1.332547047
HGLibB_55752	ZNF333	1.331614083
HGLibB_28478	MBOAT2	1.330683119
HGLibA_47497	STK36	1.329754147
HGLibA_42659	SAMD1	1.329754147
HGLibA_32536	NPTX2	1.328827157
HGLibA_51441	TRIM25	1.328827157
HGLibA_10036	CLIC6	1.326979093
HGLibB_10162	CLYBL	1.326979093
HGLibA_62578	hsa-mir-608	1.326058001
HGLibB_09901	CLDN8	1.326058001
HGLibA_46685	SPINK13	1.324221658
HGLibB_37627	POMZP3	1.324221658
HGLibA_50276	TMEM237	1.32330639
HGLibA_46352	SPAG16	1.32330639
HGLibA_28273	MAPK4	1.322393047
HGLibB_49882	TMEM133	1.322393047
HGLibB_41583	RNF217	1.320572103
HGLibB_45271	SLC6A3	1.320572103
HGLibA_32646	NRARP	1.319664487
HGLibA_20156	GPSM1	1.319664487
HGLibA_43852	SGPP2	1.318758763
HGLibB_47644	SUCLG2	1.317854924
HGLibA_36449	PHOX2A	1.316952962
HGLibA_29825	MREG	1.316052869
HGLibB_23579	IRGM	1.316052869
HGLibA_62918	hsa-mir-6511b-2	1.315154638
HGLibB_49735	TMCO6	1.315154638
HGLibA_34146	OR52B2	1.313363731
HGLibA_47321	STAG1	1.313363731
HGLibA_18359	GABRA2	1.312471039
HGLibA_19401	GMEB2	1.312471039
HGLibB_27380	LRRC71	1.311580178
HGLibA_30296	MT1M	1.310691141

HGLibA_52974	ULK4	1.310691141
HGLibA_18104	FSTL3	1.30980392
HGLibA_55682	ZNF257	1.30980392
HGLibB_24700	KIAA1279	1.308918508
HGLibA_60005	hsa-mir-4320	1.308034897
HGLibA_17016	FBXL6	1.308034897
HGLibA_50880	TNNI3K	1.307153081
HGLibA_29329	MKKS	1.306273051
HGLibB_30388	MTMR4	1.306273051
HGLibB_06725	C9orf41	1.305394801
HGLibB_20483	GSX2	1.305394801
HGLibA_58207	hsa-mir-21	1.304518324
HGLibB_40232	RARRES2	1.304518324
HGLibB_32678	NRROS	1.303643611
HGLibB_07886	CCDC85A	1.303643611
HGLibA_46352	SPAG16	1.302770657
HGLibB_17910	FOXP2	1.302770657
HGLibB_02743	ARHGAP27	1.301029996
HGLibA_11060	CRADD	1.300162274
HGLibB_00076	AASDH	1.300162274
HGLibB_23939	JOSD1	1.299296283
HGLibA_30852	MYO5B	1.298432015
HGLibB_50429	TMEM66	1.298432015
HGLibA_04775	BRD3	1.295849483
HGLibB_19324	GLTPD2	1.295849483
HGLibA_23377	INSIG1	1.294992041
HGLibA_30962	N4BP3	1.294992041
HGLibB_22670	IFNB1	1.294136288
HGLibA_36577	PIGP	1.293282218
HGLibA_49247	TGIF2LX	1.293282218
HGLibB_16867	FAS	1.292429824
HGLibA_48626	TBPL1	1.2915791
HGLibA_26670	LMBR1	1.290730039
HGLibA_57560	hsa-mir-1276	1.290730039
HGLibA_18287	FZD9	1.289882635
HGLibB_36871	PLA2G5	1.289882635
HGLibA_20109	GPR89B	1.289036881
HGLibA_29546	MMRN1	1.289036881
HGLibB_24708	KIAA1328	1.288192771
HGLibA_24831	KIAA1967	1.286509457
HGLibA_40905	RFC2	1.286509457
HGLibB_38477	PRKAR2A	1.284832642
HGLibA_09211	CERS5	1.283996656
HGLibB_34145	OR52M1	1.283996656
HGLibA_63508	hsa-mir-6833	1.283162277

HGLibB_48410	TBC1D10C	1.283162277
HGLibA_43330	SEMA3E	1.282329497
HGLibB_41703	ROM1	1.282329497
HGLibB_23598	IRX2	1.281498311
HGLibA_60892	hsa-mir-4696	1.281498311
HGLibA_38028	PPP1R13B	1.280668713
HGLibB_32799	NTHL1	1.280668713
HGLibA_21661	HLX	1.279840697
HGLibB_23670	ITCH	1.279840697
HGLibB_06029	C20orf96	1.279014256
HGLibA_50389	TMEM40	1.279014256
HGLibA_22863	IGJ	1.278189385
HGLibA_44647	SLC25A14	1.278189385
HGLibA_22633	IFIT3	1.277366077
HGLibB_24307	KCNMB1	1.277366077
HGLibB_56487	ZNF692	1.276544328
HGLibA_53341	USP34	1.27572413
HGLibB_26587	LIPK	1.27572413
HGLibB_22608	IFITM1	1.274905479
HGLibA_57896	hsa-mir-151a	1.274905479
HGLibB_21075	HECW1	1.274088368
HGLibB_02354	AP5M1	1.273272791
HGLibA_51657	TRMT1	1.273272791
HGLibA_45675	SMCP	1.272458743
HGLibB_19709	GPATCH2	1.272458743
HGLibA_23829	ITM2A	1.271646218
HGLibB_06046	C21orf91	1.271646218
HGLibA_64282	hsa-mir-922	1.27083521
HGLibA_55152	ZFAND3	1.270025714
HGLibA_47242	ST3GAL3	1.270025714
HGLibA_22833	IGFBP7	1.269217724
HGLibB_11097	CREBBP	1.269217724
HGLibA_34199	OR52N4	1.268411235
HGLibB_24076	KBTBD6	1.268411235
HGLibB_18142	FUNDC2	1.26760624
HGLibA_61468	hsa-mir-503	1.26760624
HGLibA_31989	NKAIN1	1.266802735
HGLibB_46369	SPATA12	1.266802735
HGLibB_33589	OR14I1	1.266000713
HGLibB_56301	ZNF606	1.266000713
HGLibA_31760	NF2	1.26520017
HGLibB_17951	FRAS1	1.26520017
HGLibA_29810	MRAP2	1.2644011
HGLibA_50170	TMEM200A	1.2644011
HGLibA_55378	ZMAT5	1.263603498

HGLibA_22809	IGF2R	1.262012674
HGLibA_08167	CCR8	1.261219442
HGLibA_64165	hsa-mir-8081	1.261219442
HGLibA_40807	REG4	1.260427656
HGLibB_50140	TMEM208	1.260427656
HGLibA_27049	LPA	1.259637311
HGLibA_24867	KIF16B	1.259637311
HGLibA_09867	CLDN17	1.258848401
HGLibA_51815	TSC2	1.258848401
HGLibA_05035	C10orf113	1.258060922
HGLibA_31871	NGF	1.257274869
HGLibA_17049	FBXO22	1.256490235
HGLibB_30769	MYO18A	1.256490235
HGLibA_30728	MYH4	1.255707017
HGLibB_37627	POMZP3	1.255707017
HGLibA_60488	hsa-mir-4511	1.254925208
HGLibA_54167	WDR72	1.254144805
HGLibB_51868	TSPAN33	1.254144805
HGLibB_43457	SERPINA11	1.252588192
HGLibA_55244	ZFP91	1.251811973
HGLibA_32691	NRIP1	1.251811973
HGLibB_45723	SMLR1	1.251037139
HGLibB_11084	CREB3L2	1.250263684
HGLibA_34531	OR8H3	1.249491605
HGLibA_59675	hsa-mir-409	1.249491605
HGLibA_17608	FLG	1.248720896
HGLibB_19912	GPR153	1.248720896
HGLibA_52692	UBE4B	1.247951552
HGLibA_11766	CUEDC1	1.247183569
HGLibA_40005	RABEPK	1.247183569
HGLibA_37266	PLXNA4	1.246416941
HGLibB_48619	TBX6	1.246416941
HGLibA_48030	SYNRG	1.245651664
HGLibB_37193	PLSCR5	1.245651664
HGLibA_43438	3-Sep	1.244887734
HGLibB_56230	ZNF577	1.244887734
HGLibA_53558	VAX1	1.244125144
HGLibA_56786	ZNF800	1.244125144
HGLibA_07742	CCDC27	1.243363892
HGLibA_35076	PALM3	1.243363892
HGLibB_38935	PRTN3	1.242603971
HGLibB_16715	FAM81B	1.241845378
HGLibA_37184	PLK1	1.241845378
HGLibB_38921	PRSS58	1.241088108
HGLibB_31670	NEU2	1.240332155

HGLibB_04951	BTK	1.240332155
HGLibA_25372	KPNA7	1.239577517
HGLibB_30222	MST4	1.239577517
HGLibA_02291	AP1M1	1.238824187
HGLibA_53801	VPS4B	1.238824187
HGLibA_23602	IRF8	1.238072162
HGLibB_02953	ARL17B	1.238072162
HGLibA_41211	RHOV	1.237321436
HGLibA_19923	GPR149	1.236572006
HGLibB_14001	DSG1	1.236572006
HGLibB_28449	MBD5	1.235823868
HGLibA_08781	CDK5RAP1	1.235077015
HGLibA_35823	PDE7A	1.235077015
HGLibA_34041	OR4M1	1.234331445
HGLibA_25941	LAMA1	1.234331445
HGLibA_56431	ZNF629	1.233587153
HGLibA_50404	TMEM44	1.232844134
HGLibB_35081	PAPD7	1.232844134
HGLibB_19835	GPR116	1.232102384
HGLibB_42947	SCN7A	1.232102384
HGLibB_50569	TMPRSS13	1.231361899
HGLibA_49641	TLR1	1.230622674
HGLibB_38094	PPP2R1B	1.230622674
HGLibA_10066	CLLU1OS	1.229884705
HGLibB_10340	CNOT7	1.229884705
HGLibB_10022	CLIC4	1.229147988
HGLibA_49112	TEX9	1.229147988
HGLibA_31881	NGLY1	1.228412519
HGLibB_23119	IL36G	1.228412519
HGLibA_33218	OAZ3	1.227678293
HGLibA_53456	UTP6	1.227678293
HGLibB_19707	GPATCH1	1.226945307
HGLibA_20088	GPR82	1.226213555
HGLibB_47020	SRSF4	1.226213555
HGLibA_25555	KRT80	1.225483034
HGLibA_60428	hsa-mir-4500	1.225483034
HGLibA_25657	KRTAP16-1	1.22475374
HGLibA_50963	TOMM34	1.22475374
HGLibB_20504	GTF2E1	1.224025669
HGLibA_49213	TGFB2	1.223298816
HGLibB_42961	SCNN1A	1.223298816
HGLibB_33164	OASL	1.222573178
HGLibB_25559	KRTAP10-10	1.22184875
HGLibB_55260	ZIC3	1.22184875
HGLibA_54678	YIPF4	1.220403509

HGLibB_19533	GNMT	1.220403509
HGLibA_27643	LUZP4	1.219682688
HGLibA_46294	SP140L	1.219682688
HGLibA_43914	SH3BGR	1.218963061
HGLibA_61659	hsa-mir-518b	1.218963061
HGLibA_53248	USP17L21	1.218244625
HGLibA_61302	hsa-mir-4799	1.218244625
HGLibA_34125	OR51M1	1.216096421
HGLibB_19870	GPR137	1.216096421
HGLibA_18016	FRMD4B	1.215382707
HGLibB_56013	ZNF485	1.215382707
HGLibA_39238	PSTPIP1	1.214670165
HGLibB_37793	PPARG	1.214670165
HGLibA_12091	CYP26C1	1.21395879
HGLibA_36491	PI4K2A	1.21395879
HGLibA_33676	OR1J2	1.213248578
HGLibA_16226	FAM162B	1.213248578
HGLibB_44411	SLC17A3	1.211831629
HGLibA_46961	SRF	1.211124884
HGLibB_00604	ACTBL2	1.211124884
HGLibA_12095	CYP27A1	1.210419288
HGLibB_50347	TMEM45A	1.210419288
HGLibA_25068	KLF3	1.209714836
HGLibA_56285	ZNF573	1.209714836
HGLibA_53167	USF1	1.209011525
HGLibB_49101	TFE3	1.209011525
HGLibA_29127	MGAM	1.208309351
HGLibB_00595	ACTA1	1.208309351
HGLibB_45042	SLC39A11	1.207608311
HGLibA_53132	URAD	1.2069084
HGLibB_43009	SCRT2	1.2069084
HGLibA_60343	hsa-mir-4481	1.205511953
HGLibB_50618	TMTC4	1.205511953
HGLibB_31892	NIN	1.20481541
HGLibA_49746	TMBIM6	1.20481541
HGLibA_43384	SENP1	1.204119983
HGLibA_54555	XPNPEP2	1.203425667
HGLibA_38119	PPP1R42	1.202732459
HGLibA_38282	PRAMEF10	1.202040356
HGLibA_32550	NPY1R	1.200659451
HGLibB_24523	KHDC1	1.200659451
HGLibA_62713	hsa-mir-618	1.199970641
HGLibA_51674	TRMT12	1.199970641
HGLibA_33115	NUTMF	1.199282922
HGLibB_17357	FGF17	1.199282922

HGLibA_26657	LMAN1	1.19859629
HGLibB_44927	SLC35D2	1.19859629
HGLibB_41741	RP2	1.197910742
HGLibA_27190	LRIT3	1.197910742
HGLibA_23173	IL4R	1.197226275
HGLibA_62276	hsa-mir-569	1.197226275
HGLibA_31978	NIPSNAP3B	1.196542884
HGLibA_35068	PALM	1.196542884
HGLibA_34656	OSBPL3	1.195860568
HGLibB_05159	C11orf58	1.195860568
HGLibB_50187	TMEM225	1.195179321
HGLibB_07290	CASP1	1.194499142
HGLibB_48310	TAS2R13	1.194499142
HGLibA_06679	C9orf135	1.193820026
HGLibA_17670	FLT3LG	1.193820026
HGLibA_64058	hsa-mir-8055	1.19314197
HGLibA_37981	PPM1H	1.192464972
HGLibA_04573	BLOC1S6	1.192464972
HGLibA_04431	BET1	1.191789027
HGLibA_23580	IRF2BP2	1.191789027
HGLibA_16434	FAM208B	1.191114133
HGLibA_51278	TRAK2	1.191114133
HGLibA_50873	TNNI2	1.190440285
HGLibB_11270	CRYGN	1.190440285
HGLibB_13142	DHRSX_Y	1.189095719
HGLibB_55264	ZIC4	1.188424994
HGLibA_54346	WNT10A	1.187755303
HGLibA_56790	ZNF804B	1.187755303
HGLibA_36947	PLAC9	1.187086643
HGLibB_55849	ZNF396	1.187086643
HGLibB_36841	PLA2G2D	1.186419011
HGLibA_07728	CCDC19	1.185752404
HGLibB_14772	EIF3K	1.185752404
HGLibA_44259	SIX6	1.185086819
HGLibB_00789	ADAMTS1	1.185086819
HGLibB_36819	PLA2G10	1.184422252
HGLibA_40397	RASSF9	1.1837587
HGLibA_56744	ZNF783	1.1837587
HGLibA_53211	USP16	1.183096161
HGLibA_55191	ZFP2	1.183096161
HGLibA_54761	YY1AP1	1.18243463
HGLibB_55134	ZFP30	1.18243463
HGLibA_55063	ZDBF2	1.181774106
HGLibB_42733	SAV1	1.181774106
HGLibA_31643	NEK11	1.181114585

HGLibB_50365	TMEM50B	1.181114585
HGLibA_03793	ATP9A	1.180456064
HGLibB_17868	FOXJ3	1.180456064
HGLibB_08924	CEACAM20	1.179798541
HGLibA_19458	GNAI3	1.179798541
HGLibB_16883	FASTKD3	1.179142011
HGLibB_09917	CLEC11A	1.178486472
HGLibB_41646	RNF7	1.178486472
HGLibB_36321	PHF21A	1.177831921
HGLibA_51700	TRMT61B	1.176525771
HGLibA_26565	LIN37	1.176525771
HGLibA_20783	H3F3C	1.175874166
HGLibA_56452	ZNF646	1.175874166
HGLibA_53160	USB1	1.174573882
HGLibA_60108	hsa-mir-4430	1.174573882
HGLibA_59221	hsa-mir-3667	1.173925197
HGLibA_45730	SMIM14	1.173925197
HGLibA_37285	PM20D1	1.17327748
HGLibB_36709	PITPNM3	1.17327748
HGLibB_36569	PIH1D3	1.172630727
HGLibA_29546	MMRN1	1.172630727
HGLibA_06448	C6orf141	1.171984936
HGLibA_56766	ZNF791	1.171984936
HGLibA_53160	USB1	1.171340103
HGLibA_56794	ZNF805	1.171340103
HGLibA_53047	UNK	1.170696227
HGLibB_17399	FGF9	1.170696227
HGLibA_48456	TAZ	1.170053304
HGLibA_55383	ZMIZ2	1.170053304
HGLibA_44958	SLC35A5	1.169411331
HGLibA_24201	KCNG2	1.168770306
HGLibB_39841	RAB36	1.167491087
HGLibB_38487	PRKCB	1.166852888
HGLibB_17460	FHL2	1.166215625
HGLibA_32746	NSD1	1.166215625
HGLibA_01832	AMPH	1.165579296
HGLibB_09275	CFL2	1.165579296
HGLibA_57522	hsa-mir-1271	1.164943898
HGLibA_34497	OR8B2	1.164943898
HGLibB_06029	C20orf96	1.164309429
HGLibA_15107	ENAM	1.163675884
HGLibA_49356	THG1L	1.163675884
HGLibB_39982	RABL3	1.163043263
HGLibA_11512	CT45A6	1.162411562
HGLibB_23955	JPH4	1.162411562

HGLibB_04859	BSCL2	1.161780778
HGLibB_10340	CNOT7	1.161780778
HGLibA_30429	MTMR4	1.161150909
HGLibB_33724	OR2B3	1.161150909
HGLibA_42262	RRNAD1	1.160521953
HGLibA_46294	SP140L	1.160521953
HGLibA_03810	ATR	1.159893906
HGLibB_04041	B9D1	1.159893906
HGLibB_02844	ARHGEF3	1.159266765
HGLibB_28268	MAPKBP1	1.159266765
HGLibB_05382	C15orf38	1.15864053
HGLibA_61385	hsa-mir-496	1.15864053
HGLibB_43365	SEPP1	1.158015195
HGLibB_27204	LRRC1	1.15739076
HGLibB_36611	PIK3R4	1.15739076
HGLibB_23654	IST1	1.156767222
HGLibA_07796	CCDC51	1.156144577
HGLibA_39760	QTRT1	1.156144577
HGLibA_47760	SULT2B1	1.155522824
HGLibA_34199	OR52N4	1.15490196
HGLibA_13247	DIP2A	1.15490196
HGLibA_08356	CD34	1.154281982
HGLibB_16135	FAM150A	1.154281982
HGLibA_31855	NFYA	1.153044675
HGLibB_16597	FAM47E-STBD1	1.153044675
HGLibA_56786	ZNF800	1.151810883
HGLibB_02257	ANXA8L2	1.151195299
HGLibA_47002	SRP19	1.150580586
HGLibB_56891	ZP4	1.150580586
HGLibA_52974	ULK4	1.149966742
HGLibA_55316	ZHX2	1.149966742
HGLibA_54358	WNT2	1.149353765
HGLibA_47036	SRPX2	1.149353765
HGLibA_29164	MGMT	1.148741651
HGLibB_01643	ALKBH1	1.148741651
HGLibA_04046	B9D2	1.147520006
HGLibB_49963	TMEM161A	1.147520006
HGLibA_63042	hsa-mir-6720	1.14691047
HGLibA_31710	NEU1	1.14691047
HGLibA_12346	DAW1	1.146301788
HGLibA_38119	PPP1R42	1.146301788
HGLibB_43644	SF3A3	1.145693958
HGLibA_50470	TMEM62	1.145086978
HGLibB_22101	HS3ST3B1	1.145086978
HGLibB_03360	ASPRV1	1.144480844

HGLibA_31890	NHLH1	1.143875556
HGLibA_35372	PCBP3	1.143875556
HGLibA_23820	ITK	1.14327111
HGLibB_49207	TGM5	1.14327111
HGLibA_19261	GLIPR1L1	1.142667504
HGLibB_16693	FAM73A	1.142667504
HGLibA_26657	LMAN1	1.141462802
HGLibB_16163	FAM156A	1.141462802
HGLibB_56855	ZNHIT1	1.140861703
HGLibB_52260	TUFM	1.140261434
HGLibB_03689	ATP5SL	1.140261434
HGLibA_37422	PNPLA7	1.139661993
HGLibB_41583	RNF217	1.139661993
HGLibA_07522	CCDC106	1.139063379
HGLibA_55691	ZNF260	1.139063379
HGLibA_54214	WDR90	1.138465589
HGLibB_02432	APLN	1.138465589
HGLibA_49021	TES	1.137868621
HGLibA_10421	CNTNAP2	1.137868621
HGLibB_15816	EZR	1.137272472
HGLibA_49112	TEX9	1.136082623
HGLibB_22895	IKBKG	1.136082623
HGLibA_48665	TBX22	1.135488919
HGLibA_57099	hsa-let-7a-1	1.135488919
HGLibA_19145	GJA4	1.134896025
HGLibA_29288	MIOX	1.134896025
HGLibA_11664	CTNS	1.13430394
HGLibB_36496	PIGA	1.13430394
HGLibA_50816	TNFSF15	1.133712661
HGLibB_30559	MVP	1.133712661
HGLibB_20591	GTPBP6_Y	1.133122186
HGLibA_50186	TMEM203	1.133122186
HGLibA_03907	AVPR1B	1.132532512
HGLibB_44667	SLC25A36	1.132532512
HGLibA_05714	C19orf81	1.131943638
HGLibB_11539	CT47B1	1.131943638
HGLibA_49714	TM7SF2	1.131355562
HGLibB_34860	P4HA3	1.131355562
HGLibA_56522	ZNF679	1.13076828
HGLibA_30105	MRPS5	1.13076828
HGLibA_34152	OR52B6	1.130181792
HGLibA_51811	TSC1	1.129596095
HGLibA_03972	B3GAT1	1.129011186
HGLibB_55239	ZGPAT	1.129011186
HGLibA_24793	KIAA1614	1.128427064

HGLibA_47932	SYCP2	1.128427064
HGLibA_26784	LOC100130370	1.127843727
HGLibB_55164	ZFP69	1.127843727
HGLibA_54502	XDH	1.127261173
HGLibB_41024	RGS19	1.127261173
HGLibB_55253	ZIC1	1.126679398
HGLibB_11079	CREB3	1.126098402
HGLibB_39824	RAB31	1.126098402
HGLibA_13800	DOPEY1	1.125518182
HGLibB_35425	PCDHB10	1.125518182
HGLibA_49173	TFF2	1.124938737
HGLibB_39525	PTX4	1.124938737
HGLibB_16308	FAM184B	1.124360063
HGLibA_32646	NRARP	1.124360063
HGLibB_35947	PDZK1IP1	1.123782159
HGLibA_49188	TFPI2	1.122628654
HGLibB_26859	LOC154872	1.122628654
HGLibA_38486	PRIM2	1.122053048
HGLibA_06475	C6orf222	1.122053048
HGLibA_55512	ZNF160	1.121478204
HGLibA_53950	WAS	1.12090412
HGLibB_24801	KIAA1984	1.12090412
HGLibA_63926	hsa-mir-766	1.120330794
HGLibB_30687	MYH6	1.120330794
HGLibB_55580	ZNF235	1.119758224
HGLibA_54274	WFDC8	1.119186408
HGLibB_22708	IFRD2	1.119186408
HGLibB_17669	FMNL1	1.118615343
HGLibB_42940	SCN4A	1.118615343
HGLibA_04278	BCKDHA	1.117475462
HGLibB_02794	ARHGDIA	1.117475462
HGLibA_43022	SCNN1G	1.116906641
HGLibA_15307	EPHA8	1.116906641
HGLibA_08918	CDYL	1.116338565
HGLibA_58900	hsa-mir-3199-2	1.116338565
HGLibB_22769	IGF2BP1	1.11577123
HGLibA_38990	PRUNE2	1.115204636
HGLibA_51674	TRMT12	1.115204636
HGLibA_16675	FAM65C	1.11463878
HGLibB_12834	DEFB112	1.11463878
HGLibB_12761	DECR1	1.113509275
HGLibB_24076	KBTBD6	1.113509275
HGLibA_47054	SRRM5	1.112945622
HGLibB_32517	NQO1	1.112945622
HGLibA_09367	CHCHD3	1.1123827

HGLibB_37420	POFUT1	1.1123827
HGLibB_00677	ACTRT1	1.111820506
HGLibA_63591	hsa-mir-6855	1.111820506
HGLibA_52296	TUBD1	1.111259039
HGLibA_36482	PI15	1.111259039
HGLibA_26081	LBR	1.110698297
HGLibA_41521	RNF14	1.110698297
HGLibA_30301	MT2A	1.110138279
HGLibB_51599	TRMT10B	1.110138279
HGLibB_33047	NUPR1L	1.109578981
HGLibB_07629	CCDC148	1.109578981
HGLibA_26174	LCN15	1.109020403
HGLibB_46317	SPAG9	1.109020403
HGLibB_22187	HSDL2	1.108462542
HGLibA_55979	ZNF430	1.108462542
HGLibB_24306	KCNMA1	1.107905397
HGLibA_39725	QKI	1.107348966
HGLibB_12399	DCAF10	1.107348966
HGLibA_29495	MMP15	1.106793247
HGLibB_03841	ATXN3	1.106793247
HGLibA_52208	TTLL5	1.105683937
HGLibB_05806	C1orf189	1.105683937
HGLibA_11747	CTTNBP2NL	1.105130343
HGLibA_08209	CCT6B	1.104577454
HGLibB_14096	DUS2L	1.104577454
HGLibA_00126	ABCA8	1.104025268
HGLibA_55481	ZNF14	1.104025268
HGLibA_54344	WNT1	1.103473783
HGLibA_29042	MFAP2	1.103473783
HGLibB_52612	UBE3A	1.102922997
HGLibA_21116	HELQ	1.102372909
HGLibA_25830	KRTAP9-1	1.102372909
HGLibB_03052	ARMCX5-GPRASP2	1.101823517
HGLibA_26606	LIPE	1.101274818
HGLibB_37355	PNPLA1	1.101274818
HGLibB_23593	IRS4	1.100726813
HGLibA_58984	hsa-mir-329-2	1.100179498
HGLibA_57343	hsa-mir-1238	1.100179498
HGLibA_46394	SPANXD	1.099632871
HGLibA_56072	ZNF480	1.099632871
HGLibA_56487	ZNF665	1.099086932
HGLibA_53477	UVSSA	1.098541679
HGLibB_55048	ZDHHC3	1.098541679
HGLibA_55020	ZCCHC17	1.097997109
HGLibB_11832	CXCL10	1.097997109

HGLibB_14509	EFCAB14	1.097453221
HGLibB_32456	NPPA	1.096910013
HGLibB_00291	ABI1	1.096367484
HGLibA_56188	ZNF540	1.096367484
HGLibA_53242	USP17L2	1.095284455
HGLibB_39620	PYCARD	1.095284455
HGLibA_13942	DR1	1.094743951
HGLibA_27190	LRIT3	1.094743951
HGLibA_17698	FMNL3	1.09420412
HGLibA_34531	OR8H3	1.09420412
HGLibA_02553	APOOL	1.093664958
HGLibB_31679	NEURL	1.093664958
HGLibA_47677	STXBP5L	1.093126465
HGLibB_23654	IST1	1.093126465
HGLibA_60240	hsa-mir-4458	1.092588639
HGLibB_55013	ZDHHC15	1.092588639
HGLibA_55133	ZDHHC9	1.091514981
HGLibA_31334	NCOA1	1.091514981
HGLibB_39248	PTER	1.090979146
HGLibA_38672	PROKR1	1.089909454
HGLibB_38526	PRKDC	1.089909454
HGLibA_12278	DACH2	1.089375595
HGLibB_45213	SLC5A2	1.089375595
HGLibA_34283	OR5D18	1.088842391
HGLibA_45792	SMO	1.088842391
HGLibA_18644	GAS2	1.088309841
HGLibA_61043	hsa-mir-4736	1.088309841
HGLibA_17412	FGF5	1.087777943
HGLibB_34984	PAIP2	1.087777943
HGLibA_50009	TMEM155	1.087246696
HGLibB_28784	MEF2A	1.087246696
HGLibA_58796	hsa-mir-3179-3	1.086716098
HGLibA_50659	TMSB15A	1.086716098
HGLibA_39824	RAB18	1.086186148
HGLibB_34332	OR6B2	1.086186148
HGLibA_42129	RPS27L	1.085656843
HGLibB_15493	ERN2	1.085656843
HGLibA_62165	hsa-mir-5581	1.085128182
HGLibB_55306	ZMAT3	1.085128182
HGLibA_54214	WDR90	1.084600165
HGLibA_47002	SRP19	1.084600165
HGLibA_11268	CRYBB3	1.084072788
HGLibB_32448	NPM3	1.084072788
HGLibA_29850	MRGPRX2	1.083546051
HGLibA_55475	ZNF136	1.083546051

HGLibA_54087	WDR41	1.083019953
HGLibB_45143	SLC47A2	1.083019953
HGLibA_16617	FAM47E	1.08249449
HGLibA_47269	ST6GALNAC3	1.08249449
HGLibA_52262	TUBA4A	1.081969663
HGLibA_24225	KCNH6	1.081445469
HGLibB_20822	HAPLN4	1.081445469
HGLibA_22559	IDNK	1.080921908
HGLibB_15174	ENTPD1	1.080921908
HGLibB_12834	DEFB112	1.080398976
HGLibA_29261	MIF	1.080398976
HGLibB_21508	HIST2H2BF	1.079876674
HGLibB_13349	DLST	1.079354999
HGLibB_10593	COL6A5	1.079354999
HGLibA_44633	SLC25A1	1.078833949
HGLibB_40158	RANBP6	1.078833949
HGLibB_22340	HTR2A	1.078313525
HGLibA_39789	RAB11B	1.078313525
HGLibB_46132	SORCS3	1.077793723
HGLibA_23708	ITFG2	1.077274542
HGLibB_26874	LOC283710	1.077274542
HGLibB_02789	ARHGAP9	1.076755981
HGLibB_22819	IGFLR1	1.075720714
HGLibA_31971	NIPSNAP1	1.075204004
HGLibB_35764	PDE6C	1.075204004
HGLibA_61350	hsa-mir-487b	1.074687909
HGLibB_27868	MAGEA2B	1.074687909
HGLibA_59048	hsa-mir-3529	1.074172425
HGLibB_27168	LRP1B	1.074172425
HGLibA_49536	TIMP1	1.073657553
HGLibA_60195	hsa-mir-4447	1.073657553
HGLibA_58631	hsa-mir-3143	1.073143291
HGLibA_50483	TMEM64	1.073143291
HGLibA_22697	IFNAR2	1.072629637
HGLibB_29116	MGEA5	1.072629637
HGLibA_51830	TSEN15	1.071604148
HGLibB_46484	SPATC1L	1.071604148
HGLibA_49581	TK1	1.07109231
HGLibB_08815	CDKN1A	1.07109231
HGLibA_09361	CHCHD10	1.070581074
HGLibA_57181	hsa-mir-105-2	1.070581074
HGLibA_32805	NT5C1B	1.07007044
HGLibA_30490	MTRNR2L8	1.07007044
HGLibA_19413	GML	1.069050969
HGLibB_46451	SPATA32	1.069050969

HGLibA_44535	SLC22A1	1.068542129
HGLibA_30886	MYOG	1.068542129
HGLibB_15304	EPHX2	1.068033885
HGLibA_31760	NF2	1.067526235
HGLibB_12289	DAK	1.067526235
HGLibA_35530	PCDHGA2	1.067019178
HGLibB_04388	BDP1	1.067019178
HGLibA_60367	hsa-mir-4487	1.066006836
HGLibB_02267	AOC2	1.066006836
HGLibA_41463	RNF11	1.065501549
HGLibA_57902	hsa-mir-152	1.065501549
HGLibA_32638	NR5A2	1.064996849
HGLibB_12438	DCAF6	1.064996849
HGLibA_44524	SLC1A7	1.064492734
HGLibA_30249	MSS51	1.063989204
HGLibB_32782	NT5DC3	1.063989204
HGLibB_21481	HIST1H4I	1.063486258
HGLibB_37355	PNPLA1	1.063486258
HGLibA_24370	KCNQ2	1.062983893
HGLibA_23287	ING2	1.062482108
HGLibA_32978	NUDT4	1.062482108
HGLibA_18792	GCKR	1.061980903
HGLibA_12807	DEFA6	1.061980903
HGLibA_08805	CDKL1	1.061480275
HGLibB_56447	ZNF676	1.061480275
HGLibA_59135	hsa-mir-3646	1.060480747
HGLibB_01219	AGPAT2	1.060480747
HGLibA_50273	TMEM236	1.059483515
HGLibA_38130	PPP1R9B	1.059483515
HGLibA_07055	CALY	1.058985756
HGLibA_47321	STAG1	1.058985756
HGLibA_39760	QTRT1	1.058488567
HGLibA_39198	PSME4	1.057991947
HGLibA_12087	CYP26B1	1.057495894
HGLibB_46355	SPANXN4	1.057495894
HGLibB_39436	PTPRB	1.057000407
HGLibA_48693	TCAP	1.057000407
HGLibA_35042	PAK2	1.056505484
HGLibB_26944	LOC650293	1.056505484
HGLibA_40969	RFX3	1.056011125
HGLibB_26320	LGALS16	1.056011125
HGLibB_04790	BRDT	1.055517328
HGLibA_60980	hsa-mir-4721	1.055517328
HGLibA_39820	RAB17	1.055024092
HGLibA_31978	NIPSNAP3B	1.055024092

HGLibA_17964	FPR1	1.054531415
HGLibA_34581	OR9K2	1.054531415
HGLibA_45893	SNAPC2	1.054039296
HGLibA_05809	C1orf189	1.053547735
HGLibA_56285	ZNF573	1.053547735
HGLibB_05971	C1S	1.053056729
HGLibA_60431	hsa-mir-4501	1.052566278
HGLibA_49804	TMED2	1.05207638
HGLibA_08705	CDHR3	1.051587034
HGLibB_37607	POMGNT1	1.051587034
HGLibA_61198	hsa-mir-4773-2	1.051098239
HGLibB_23629	ISG20L2	1.051098239
HGLibA_58103	hsa-mir-197	1.050609993
HGLibA_56072	ZNF480	1.050609993
HGLibA_53923	VWC2	1.050122296
HGLibB_05471	C16orf71	1.050122296
HGLibA_50071	TMEM176A	1.049635146
HGLibB_50361	TMEM5	1.049635146
HGLibA_43556	SERPINB5	1.049148541
HGLibB_50897	TOMM22	1.049148541
HGLibA_25982	LAMP3	1.048662481
HGLibB_03022	ARMC5	1.048662481
HGLibA_15898	FA2H	1.048176965
HGLibB_40010	RAD18	1.048176965
HGLibB_42686	SAR1B	1.04769199
HGLibA_35505	PCDHB6	1.047207557
HGLibA_26077	LBH	1.047207557
HGLibA_01328	AIDA	1.046240308
HGLibB_27472	LSAMP	1.046240308
HGLibB_23884	JAK3	1.045757491
HGLibA_55349	ZKSCAN2	1.045757491
HGLibA_54344	WNT1	1.045275209
HGLibA_16050	FAM118B	1.045275209
HGLibB_49881	TMEM132E	1.044793462
HGLibA_23727	ITGA2B	1.04431225
HGLibA_59051	hsa-mir-3591	1.04431225
HGLibA_46968	SRGAP1	1.04383157
HGLibA_12172	CYP4A11	1.04383157
HGLibB_47669	SULF1	1.043351421
HGLibB_34007	OR4N5	1.042871802
HGLibB_01931	ANK1	1.042392713
HGLibA_23678	ISM2	1.042392713
HGLibA_56885	ZNF865	1.041914151
HGLibA_52365	TVP23C-CDRT4	1.041436117
HGLibA_45259	SLC5A11	1.041436117

HGLibA_42631	SAE1	1.040958608
HGLibB_26030	LATS2	1.040958608
HGLibA_27020	LONRF1	1.040481623
HGLibB_23178	ILDR1	1.040481623
HGLibA_28276	MAPK6	1.040005162
HGLibA_36622	PIK3C2A	1.040005162
HGLibA_30908	MYOZ3	1.039529222
HGLibB_24251	KCNJ6	1.039529222
HGLibA_26044	LARP7	1.039053804
HGLibB_44833	SLC2A6	1.039053804
HGLibA_11392	CSNK1G1	1.038578906
HGLibA_44886	SLC2A6	1.037630664
HGLibB_22490	ICT1	1.037157319
HGLibA_30081	MRPS27	1.036684489
HGLibB_19668	GOT2	1.036684489
HGLibA_25803	KRTAP5-6	1.036212173
HGLibB_16988	FBXL5	1.036212173
HGLibA_62920	hsa-mir-6512	1.03574037
HGLibB_03377	ASTN1	1.03574037
HGLibA_49267	TGM6	1.035269079
HGLibB_38102	PPP2R2C	1.035269079
HGLibA_11218	CRTAC1	1.034798299
HGLibB_24124	KCNB2	1.034798299
HGLibA_24646	KIAA0586	1.034328029
HGLibB_08388	CD47	1.034328029
HGLibA_56063	ZNF474	1.033858267
HGLibB_43306	SEMA5A	1.033858267
HGLibB_12694	DDX4	1.033389013
HGLibB_53916	WBP4	1.033389013
HGLibA_55504	ZNF155	1.032920266
HGLibB_22254	HSPA4	1.032920266
HGLibA_56653	ZNF736	1.032452024
HGLibB_18092	FTH1	1.032452024
HGLibA_49267	TGM6	1.031517051
HGLibB_46062	SOAT2	1.031517051
HGLibA_10215	CMTM3	1.031050319
HGLibB_31587	NEIL1	1.031050319
HGLibB_22932	IL12B	1.030584088
HGLibA_19439	GNA12	1.030584088
HGLibB_48057	SZT2	1.030118356
HGLibB_44517	SLC22A20	1.029188389
HGLibB_10719	COPS7B	1.029188389
HGLibA_50775	TNFRSF1B	1.028724151
HGLibA_24748	KIAA1407	1.028260409
HGLibB_44512	SLC22A18AS	1.028260409

HGLibB_26460	LILRA4	1.027797162
HGLibA_43020	SCNN1D	1.027797162
HGLibA_00459	ACN9	1.027334408
HGLibA_29168	MGP	1.027334408
HGLibA_08108	CCNJ	1.026872146
HGLibA_52151	TTC7A	1.026872146
HGLibA_25584	KRTAP1-4	1.026410377
HGLibA_58488	hsa-mir-30d	1.026410377
HGLibA_12858	DEFB115	1.025949097
HGLibB_24523	KHDC1	1.025949097
HGLibB_41147	RHOQ	1.025488307
HGLibA_61213	hsa-mir-4776-2	1.025028006
HGLibB_50214	TMEM236	1.025028006
HGLibA_17490	FHL5	1.024568191
HGLibB_56081	ZNF518A	1.024568191
HGLibA_46133	SOCS4	1.024108864
HGLibB_19705	GPAT2	1.024108864
HGLibA_33313	OGFOD3	1.023650021
HGLibB_20706	H1F0	1.023650021
HGLibB_48180	TAF5	1.023191663
HGLibB_27832	MAFA	1.022733788
HGLibA_49951	TMEM136	1.022733788
HGLibA_46153	SOGA1	1.022276395
HGLibA_35967	PDYN	1.022276395
HGLibB_54879	ZC3H10	1.021819483
HGLibB_51697	TRPM4	1.021363052
HGLibB_10875	CPA2	1.021363052
HGLibA_03433	ATF2	1.020907099
HGLibA_45046	SLC37A2	1.020907099
HGLibA_06259	C3orf67	1.020451625
HGLibB_02685	ARG2	1.020451625
HGLibA_62545	hsa-mir-6073	1.019996628
HGLibB_26594	LIPN	1.019996628
HGLibA_18692	GATC	1.019542108
HGLibB_31679	NEURL	1.019542108
HGLibA_02650	ARF3	1.019088062
HGLibA_00283	ABHD6	1.019088062
HGLibA_55355	ZKSCAN4	1.018634491
HGLibA_54659	YEATS4	1.018181393
HGLibA_34850	P2RX2	1.018181393
HGLibA_07427	CBS	1.017728767
HGLibA_59980	hsa-mir-4315-2	1.017728767
HGLibB_15641	ETV5	1.017276612
HGLibA_38990	PRUNE2	1.016824928
HGLibA_62971	hsa-mir-659	1.016824928

HGLibA_59666	hsa-mir-3977	1.016373713
HGLibA_45420	SLC9A6	1.016373713
HGLibA_10093	CLOCK	1.015922966
HGLibA_45370	SLC7A5	1.015472687
HGLibA_16195	FAM157B	1.015022874
HGLibB_19835	GPR116	1.015022874
HGLibB_08772	CDK5R2	1.014573526
HGLibB_07034	CALML6	1.014573526
HGLibB_37694	POTEJ	1.014124643
HGLibA_51836	TSEN34	1.013676223
HGLibB_53768	VRTN	1.013676223
HGLibA_37461	PODXL2	1.013228266
HGLibB_46145	SOS1	1.013228266
HGLibA_40455	RBFOX1	1.01278077
HGLibB_56421	ZNF665	1.01278077
HGLibA_53120	UQCRFS1	1.012333735
HGLibA_15791	EXOSC7	1.012333735
HGLibA_49657	TLR6	1.01188716
HGLibA_44572	SLC22A23	1.011441043
HGLibB_40892	RFPL4B	1.011441043
HGLibA_50798	TNFSF11	1.010995384
HGLibA_52510	UAP1L1	1.010995384
HGLibA_16418	FAM203A	1.010550182
HGLibB_41638	RNF5	1.010550182
HGLibA_31822	NFKBIA	1.010105436
HGLibB_12464	DCD	1.010105436
HGLibA_45475	SLCO5A1	1.009661145
HGLibA_18558	GALNT3	1.009217308
HGLibB_38869	PRSS23	1.009217308
HGLibB_29773	MRAS	1.008773924
HGLibB_03209	ASB12	1.008330993
HGLibB_32732	NSMF	1.008330993
HGLibB_00412	ACCS	1.007888512
HGLibB_16125	FAM13C	1.007446482
HGLibA_46798	SPRN	1.007004902
HGLibB_11124	CRH	1.007004902
HGLibA_55843	ZNF343	1.00656377
HGLibA_53704	VN1R4	1.006123085
HGLibB_34727	OTUD6B	1.006123085
HGLibB_09708	CIR1	1.005682847
HGLibA_36173	PFN1	1.005682847
HGLibA_29735	MPL	1.005243055
HGLibA_45412	SLC9A3R2	1.005243055
HGLibA_36962	PLAU	1.004803708
HGLibB_13458	DNAH12	1.004803708

HGLibA_60735	hsa-mir-4657	1.004364805
HGLibB_03972	B3GAT2	1.004364805
HGLibA_40230	RAP1GAP2	1.003926346
HGLibB_54832	ZBTB46	1.003926346
HGLibA_55258	ZFR	1.003488328
HGLibB_42675	SAP30L	1.003488328
HGLibB_00900	ADCK5	1.003050752
HGLibB_21248	HIAT1	1.002176919
HGLibA_36924	PLA2G7	1.000869459
HGLibB_43622	SETX	1.000869459
HGLibA_35411	PCDH20	1.000434512
HGLibB_16920	FBLN1	1.000434512
HGLibA_38257	PQLC3	1
HGLibA_50859	TNMD	1
HGLibA_31265	NCAPD3	0.999565923
HGLibA_59796	hsa-mir-4273	0.999565923
HGLibA_48142	TAB1	0.999132278
HGLibB_27719	LYRM9	0.999132278
HGLibA_51916	TSPAN18	0.998699067
HGLibB_17341	FGF10	0.998699067
HGLibA_61360	hsa-mir-490	0.998266287
HGLibA_61057	hsa-mir-4740	0.998266287
HGLibA_35798	PDE4DIP	0.997833938
HGLibB_40225	RARB	0.997833938
HGLibA_33545	OR10S1	0.997402019
HGLibB_19189	GLA	0.997402019
HGLibB_25573	KRTAP10-4	0.996539468
HGLibA_51458	TRIM31	0.996108834
HGLibB_01451	AKIP1	0.996108834
HGLibA_41722	RNMTL1	0.995678626
HGLibB_10530	COL22A1	0.995678626
HGLibA_28834	MEF2BNB-MEF2B	0.995248844
HGLibB_41066	RGSL1	0.995248844
HGLibB_01120	AFM	0.994819487
HGLibB_40931	RFX8	0.994390555
HGLibB_02992	ARL6IP6	0.993962045
HGLibA_59051	hsa-mir-3591	0.993962045
HGLibA_18234	FXYD4	0.993533958
HGLibB_12401	DCAF11	0.993533958
HGLibA_12576	DCUN1D5	0.993106292
HGLibA_18574	GALNT9	0.993106292
HGLibB_27272	LRRC31	0.992679047
HGLibA_51058	TP53BP2	0.992252222
HGLibA_56251	ZNF563	0.992252222
HGLibA_53214	USP17L10	0.991825816

HGLibA_57616	hsa-mir-1288	0.991825816
HGLibA_41900	RPL22L1	0.991399828
HGLibA_64133	hsa-mir-8073	0.991399828
HGLibA_29673	MORN3	0.990974258
HGLibA_32746	NSD1	0.990974258
HGLibB_21081	HEG1	0.990549104
HGLibA_50063	TMEM173	0.990124366
HGLibB_28222	MAPK14	0.990124366
HGLibA_43160	SDR16C5	0.989700043
HGLibB_42822	SCARF1	0.989700043
HGLibA_15701	EVPL	0.989276135
HGLibB_16532	FAM25C	0.989276135
HGLibA_42196	RPSA	0.988852639
HGLibA_09496	CHMP5	0.988006885
HGLibA_59559	hsa-mir-3921	0.988006885
HGLibA_25838	KRTAP9-4	0.987584625
HGLibB_39333	PTK2B	0.987584625
HGLibA_47687	STYX	0.987162775
HGLibB_27424	LRRK1	0.987162775
HGLibA_08462	CD83	0.986741335
HGLibB_38826	PRRG3	0.986741335
HGLibB_25151	KLHL32	0.986320303
HGLibB_29935	MRPL4	0.986320303
HGLibA_41096	RGS5	0.985899678
HGLibA_60919	hsa-mir-4705	0.985899678
HGLibA_57560	hsa-mir-1276	0.985479461
HGLibA_20713	GYS2	0.985479461
HGLibA_12462	DCAF8L2	0.98505965
HGLibB_56970	ZSWIM1	0.98505965
HGLibA_52854	UFSP1	0.984640245
HGLibA_55496	ZNF146	0.984640245
HGLibA_49951	TMEM136	0.984221244
HGLibA_17225	FCRL1	0.983802646
HGLibA_54685	YIPF6	0.983802646
HGLibB_37163	PLOD3	0.982966661
HGLibB_04903	BTBD17	0.98254927
HGLibA_47134	SSH2	0.98254927
HGLibA_38714	PRPF19	0.982132281
HGLibB_41964	RPN1	0.982132281
HGLibA_58428	hsa-mir-301a	0.981715692
HGLibB_39062	PSMB5	0.981715692
HGLibA_26090	LCA5	0.981299501
HGLibA_39773	R3HDM1	0.981299501
HGLibB_06702	C9orf170	0.98088371
HGLibA_09208	CERS4	0.980468315

HGLibB_43537	SERPINF2	0.980468315
HGLibA_40039	RABL6	0.980053318
HGLibA_35060	PALD1	0.980053318
HGLibA_54768	ZADH2	0.979638717
HGLibB_30001	MRPS15	0.978810701
HGLibB_11124	CRH	0.978397284
HGLibB_41903	RPL36A	0.978397284
HGLibB_18954	GGA1	0.97798426
HGLibA_50659	TMSB15A	0.977571629
HGLibB_24174	KCNG4	0.977571629
HGLibA_26196	LCORL	0.977159389
HGLibB_37672	POTEB2	0.977159389
HGLibA_12078	CYP21A2	0.97674754
HGLibB_12475	DCHS1	0.97674754
HGLibA_38149	PPP2R2B	0.976336082
HGLibB_41522	RNF170	0.976336082
HGLibA_23191	IL7	0.975925013
HGLibA_55203	ZFP36	0.975925013
HGLibA_54588	XRCC3	0.975514332
HGLibA_47815	SUPT3H	0.975514332
HGLibA_25347	KNSTRN	0.97510404
HGLibB_20252	GRIK1	0.97510404
HGLibB_08725	CDK12	0.974694135
HGLibB_29062	MFSD4	0.974694135
HGLibB_26025	LAT2	0.974284616
HGLibB_39847	RAB38	0.974284616
HGLibA_22750	IFT172	0.973875483
HGLibA_00775	ADAM33	0.973875483
HGLibB_35701	PDCL	0.973466735
HGLibA_04680	BOLA1	0.973058372
HGLibB_38732	PRR14L	0.973058372
HGLibA_55699	ZNF266	0.972650392
HGLibB_21228	HHATL	0.972650392
HGLibA_59174	hsa-mir-3657	0.972242795
HGLibA_53567	VCAM1	0.972242795
HGLibB_03289	ASGR2	0.971835581
HGLibA_47241	ST3GAL2	0.971428747
HGLibA_38084	PPP1R27	0.971428747
HGLibA_46087	SNX31	0.970616222
HGLibA_45220	SLC4A4	0.970210529
HGLibB_17708	FN3KRP	0.970210529
HGLibA_53477	UVSSA	0.969805215
HGLibA_34146	OR52B2	0.969805215
HGLibA_12197	CYP4F8	0.969400278
HGLibB_40797	REPS1	0.969400278

HGLibA_08629	CDH1	0.968995719
HGLibA_38333	PRAMEF9	0.968995719
HGLibA_23829	ITM2A	0.968591536
HGLibB_15928	FAF2	0.968591536
HGLibA_45335	SLC6A6	0.967784297
HGLibB_14818	EIF4G1	0.967784297
HGLibA_23430	INTS7	0.966576245
HGLibB_04298	BCL2A1	0.966576245
HGLibA_58496	hsa-mir-31	0.966174306
HGLibA_25867	KSR2	0.965772739
HGLibA_63844	hsa-mir-7154	0.965772739
HGLibA_62811	hsa-mir-642b	0.965371543
HGLibA_56233	ZNF558	0.965371543
HGLibA_53736	VPS13C	0.964970718
HGLibB_32290	NOSIP	0.964970718
HGLibA_50423	TMEM50A	0.964570262
HGLibB_20173	GRAMD1C	0.964570262
HGLibB_05321	C14orf132	0.964170175
HGLibA_59921	hsa-mir-4302	0.963770456
HGLibA_56157	ZNF521	0.963770456
HGLibB_19250	GLIS2	0.963371105
HGLibB_35081	PAPD7	0.96297212
HGLibA_07261	CARS2	0.962573502
HGLibB_48875	TEAD4	0.962573502
HGLibA_40925	RFNG	0.962175249
HGLibA_63396	hsa-mir-6805	0.962175249
HGLibA_34777	OTUD7A	0.961777362
HGLibB_29486	MMP27	0.961777362
HGLibA_36679	PIM2	0.961379838
HGLibA_51792	TRPV4	0.961379838
HGLibA_24154	KCNAB3	0.960982678
HGLibB_56466	ZNF683	0.960982678
HGLibA_53047	UNK	0.960585881
HGLibA_59254	hsa-mir-3675	0.960585881
HGLibA_01013	ADORA2A	0.960189446
HGLibB_08991	CELA3A	0.960189446
HGLibB_31142	NAT8B	0.959793372
HGLibB_12690	DDX3Y	0.95939766
HGLibB_06046	C21orf91	0.95939766
HGLibB_40480	RBM3	0.959002308
HGLibA_16504	FAM21A	0.958607315
HGLibB_28089	MAP1LC3C	0.958607315
HGLibA_14348	EBF1	0.958212681
HGLibB_00819	ADAMTS2	0.958212681
HGLibB_28814	MEGF8	0.957818405

HGLibB_08121	CCNT2	0.957424488
HGLibB_19820	GPR110	0.957424488
HGLibA_28560	MCAT	0.957030927
HGLibA_27986	MAGEF1	0.956637722
HGLibA_49940	TMEM132E	0.956637722
HGLibA_18081	FSD1L	0.955852379
HGLibB_02503	APOC2	0.955852379
HGLibB_23670	ITCH	0.95546024
HGLibA_10992	CPS1	0.955068454
HGLibB_24227	KCNJ14	0.955068454
HGLibA_61174	hsa-mir-4769	0.954677021
HGLibB_23720	ITGAE	0.954677021
HGLibA_12632	DDR1	0.954285941
HGLibB_42881	SCGB3A2	0.954285941
HGLibA_45474	SLCO5A1	0.953895213
HGLibB_44999	SLC37A4	0.953895213
HGLibA_60527	hsa-mir-451b	0.953504836
HGLibA_50157	TMEM194B	0.953114809
HGLibA_43541	SERPINB12	0.952335805
HGLibA_58976	hsa-mir-328	0.951946827
HGLibA_52626	UBE2L3	0.951946827
HGLibA_27225	LRP5	0.951558196
HGLibB_48640	TCEA2	0.951558196
HGLibA_20463	GSTA5	0.951169913
HGLibA_01215	AGO4	0.951169913
HGLibB_53229	USP2	0.950781977
HGLibA_12169	CYP46A1	0.950394387
HGLibA_28557	MCAM	0.950394387
HGLibA_13016	DEXI	0.950007143
HGLibB_27899	MAGEB18	0.950007143
HGLibA_14192	DUSP8	0.949620244
HGLibB_01837	AMTN	0.949620244
HGLibA_28356	MARCKS	0.948847478
HGLibB_21340	HIST1H1E	0.948847478
HGLibA_47914	SYCE1	0.948461609
HGLibA_25941	LAMA1	0.948461609
HGLibB_54943	ZCCHC12	0.948076084
HGLibA_22715	IFNK	0.9476909
HGLibB_03753	ATP6V1F	0.9476909
HGLibA_36819	PKIA	0.947306058
HGLibB_55003	ZDHHC11	0.947306058
HGLibA_55175	ZFHX3	0.946921557
HGLibA_50915	TNRC6B	0.946921557
HGLibA_34093	OR51D1	0.946537395
HGLibA_60753	hsa-mir-4661	0.946537395

HGLibA_50083	TMEM178B	0.946153573
HGLibA_59993	hsa-mir-4318	0.946153573
HGLibA_13648	DNAL4	0.94577009
HGLibB_50203	TMEM233	0.94577009
HGLibA_40088	RAD51B	0.945386945
HGLibB_27787	MAB21L3	0.945386945
HGLibA_49646	TLR2	0.945004138
HGLibB_31626	NEK9	0.945004138
HGLibA_26146	LCE4A	0.944621669
HGLibB_35998	PEMT	0.944621669
HGLibB_15879	FAAH	0.944239535
HGLibB_45723	SMLR1	0.944239535
HGLibB_33779	OR2L3	0.943857738
HGLibB_39595	PXDC1	0.943857738
HGLibB_09585	CHRNB3	0.943476276
HGLibB_19310	GLT6D1	0.943095149
HGLibB_05829	C1orf226	0.942714356
HGLibA_19996	GPR20	0.942714356
HGLibA_50886	TNNT2	0.942333896
HGLibA_23107	IL25	0.94195377
HGLibB_09817	CLCN3	0.94195377
HGLibA_04412	BEND6	0.941573976
HGLibB_40931	RFX8	0.941573976
HGLibB_19870	GPR137	0.941194513
HGLibB_23380	INTS12	0.941194513
HGLibB_15324	EPN3	0.940815382
HGLibB_20601	GTSF1	0.940815382
HGLibA_04782	BRD7	0.940436582
HGLibB_16209	FAM163B	0.940436582
HGLibA_46243	SOX15	0.940058112
HGLibB_34007	OR4N5	0.940058112
HGLibA_13123	DHRS12	0.939679971
HGLibB_26710	LNX2	0.939679971
HGLibA_30184	MSC	0.93930216
HGLibB_07712	CCDC179	0.93930216
HGLibB_13918	DR1	0.938924676
HGLibB_55056	ZDHHC6	0.938547521
HGLibB_32201	NOD1	0.938170693
HGLibA_57122	hsa-let-7e	0.938170693
HGLibA_42937	SCGN	0.937418016
HGLibA_49428	THYN1	0.937418016
HGLibB_55985	ZNF470	0.936291441
HGLibB_04101	BAGE5	0.935916564
HGLibA_40134	RAG2	0.935916564
HGLibA_13013	DET1	0.935542011

HGLibA_56811	ZNF816	0.935542011
HGLibA_12798	DEFA3	0.93516778
HGLibB_07993	CCL23	0.934793872
HGLibB_51750	TSC2	0.934420285
HGLibB_21712	HMHB1	0.93404702
HGLibA_08113	CCNK	0.933674075
HGLibB_36148	PGAM1	0.933674075
HGLibA_59557	hsa-mir-3920	0.932929144
HGLibB_05403	C15orf52	0.932929144
HGLibA_50709	TNFAIP2	0.932557157
HGLibB_25066	KLHDC4	0.932557157
HGLibA_23008	IL17RB	0.932185489
HGLibB_25822	KRTCAP3	0.932185489
HGLibA_29828	MRFAP1	0.931814138
HGLibA_56231	ZNF557	0.931814138
HGLibA_53803	VPS51	0.931443105
HGLibA_62489	hsa-mir-599	0.931443105
HGLibB_23112	IL34	0.931072388
HGLibA_52589	UBE2D3	0.930701988
HGLibB_19984	GPR27	0.930701988
HGLibB_35632	PCSK5	0.930331903
HGLibA_10096	CLP1	0.929962133
HGLibB_41683	ROBO2	0.929962133
HGLibA_40698	RCL1	0.929592678
HGLibB_49379	TIAM1	0.929592678
HGLibA_19250	GLI2	0.929223537
HGLibB_17221	FCRLA	0.929223537
HGLibA_52510	UAP1L1	0.92885471
HGLibB_08923	CEACAM19	0.92885471
HGLibA_33591	OR12D3	0.928486195
HGLibA_25234	KLK10	0.928117993
HGLibB_19219	GLG1	0.928117993
HGLibA_33702	OR1M1	0.927750102
HGLibA_55367	ZMAT1	0.927750102
HGLibB_24787	KIAA1804	0.927382523
HGLibA_49568	TJAP1	0.927015255
HGLibB_18463	GAGE7	0.927015255
HGLibB_07835	CCDC68	0.926648298
HGLibB_10500	COL14A1	0.92628165
HGLibA_47827	SUPT7L	0.925549281
HGLibA_27055	LPAR2	0.925183559
HGLibB_06088	C2CD3	0.925183559
HGLibA_40692	RCE1	0.924818145
HGLibB_51160	TRA2A	0.924818145
HGLibA_64082	hsa-mir-8061	0.924088239

HGLibB_55827	ZNF384	0.924088239
HGLibA_53950	WAS	0.923723745
HGLibB_25353	KRBA2	0.923723745
HGLibA_58064	hsa-mir-1915	0.923359556
HGLibA_49444	TICAM1	0.922995673
HGLibA_40333	RASGEF1B	0.922995673
HGLibA_08712	CDIP1	0.922632095
HGLibB_41418	RNF112	0.922632095
HGLibA_28998	METTL25	0.92226882
HGLibB_19534	GNPAT	0.92190585
HGLibA_33376	OLR1	0.921543182
HGLibA_32277	NOL8	0.920818754
HGLibA_00400	ACBD4	0.920818754
HGLibA_38576	PRKDC	0.920456993
HGLibA_42701	SAMD9L	0.920095532
HGLibA_13206	DHX58	0.919373513
HGLibB_37793	PPARG	0.919373513
HGLibA_61370	hsa-mir-492	0.919012953
HGLibA_13499	DNAH8	0.918652692
HGLibB_33618	OR1E2	0.918652692
HGLibA_54894	ZBTB45	0.91829273
HGLibB_06134	C2orf48	0.91829273
HGLibA_35955	PDX1	0.917933066
HGLibA_55913	ZNF395	0.917933066
HGLibA_53536	VASN	0.917573699
HGLibA_22558	IDI2	0.917573699
HGLibA_09631	CHST2	0.91721463
HGLibA_63381	hsa-mir-6801	0.91721463
HGLibA_24298	KCNK10	0.916855857
HGLibA_47462	STK17A	0.916855857
HGLibA_17748	FNDC3A	0.91649738
HGLibB_26392	LHFPL1	0.91649738
HGLibA_52709	UBL4B	0.916139199
HGLibA_25129	KLHL12	0.916139199
HGLibA_51957	TSPO	0.915781313
HGLibA_34331	OR5M10	0.915423722
HGLibA_27669	LY6G6D	0.915423722
HGLibA_24076	KATNAL1	0.915066425
HGLibA_55881	ZNF367	0.915066425
HGLibA_53956	WASF2	0.914709422
HGLibA_38257	PQLC3	0.914709422
HGLibA_29090	MFSD12	0.914352712
HGLibB_55410	ZNF138	0.914352712
HGLibA_04711	BPIFA1	0.913996294
HGLibA_45178	SLC45A3	0.913996294

HGLibA_31100	NANOS2	0.913640169
HGLibB_18373	GABRP	0.913284336
HGLibA_01712	ALPP	0.912928794
HGLibA_33313	OGFOD3	0.912928794
HGLibA_09296	CGA	0.912573543
HGLibA_58233	hsa-mir-2115	0.912573543
HGLibA_25470	KRT3	0.912218582
HGLibA_59518	hsa-mir-3912	0.912218582
HGLibA_39697	PYGO2	0.911863911
HGLibB_47074	SSH1	0.911863911
HGLibA_56114	ZNF502	0.91150953
HGLibB_17310	FFAR4	0.91150953
HGLibA_55481	ZNF14	0.911155437
HGLibB_26218	LDOC1	0.911155437
HGLibB_17484	FICD	0.910801633
HGLibB_30219	MST1R	0.910801633
HGLibA_45140	SLC41A1	0.910448117
HGLibA_63184	hsa-mir-6757	0.910448117
HGLibA_40395	RASSF8	0.910094889
HGLibB_49847	TMEM125	0.910094889
HGLibA_01438	AKAP6	0.909741947
HGLibA_43726	SFN	0.909741947
HGLibA_23775	ITGB1BP2	0.909389292
HGLibA_57746	hsa-mir-130a	0.909389292
HGLibA_44184	SIN3A	0.909036923
HGLibB_33878	OR4A15	0.909036923
HGLibB_44688	SLC25A42	0.90868484
HGLibB_12659	DDX21	0.908333042
